# Supplementary material for: Pregnancy Risk, Infant Surveillance, and Measurement Alliance (PRISMA) Maternal and Newborn Health Study: protocol for a multisite, prospective, open cohort study of pregnancy and postpartum health outcomes in South Asia and sub-Saharan Africa
Source: BMJ Open. 2026 Jan 20;16(1):e104512. doi: 10.1136/bmjopen-2025-104512 (PMC12820853; doi:10.1136/bmjopen-2025-104512)
Supplement: Supplementary data [file bmjopen-16-1-s001.pdf]

SUPPLEMENT 1. OUTCOME DEFINITIONS

Primary Outcomes

1. Maternal mortality: Death from any cause related to or aggravated by pregnancy or its management (excluding accidental or incidental causes) during pregnancy and childbirth or within 42 days of termination of pregnancy, irrespective of the duration and site of the pregnancy.

2. Composite severe maternal outcomes: Composite outcome comprising maternal deaths, late maternal deaths, and maternal near-misses, each defined as the following:

    a. *Maternal death*: Death from any cause related to or aggravated by pregnancy or its management (excluding accidental or incidental causes) during pregnancy and childbirth or within 42 days of termination of pregnancy, irrespective of the duration and site of the pregnancy.

    b. *Late maternal death*: Death from any cause related to or aggravated by pregnancy or its management (excluding accidental or incidental causes), irrespective of the duration and site of the pregnancy, from 42 days postpartum up to one year.

    c. *Maternal near-miss*: Woman who nearly died but survived a life-threatening condition that occurred during pregnancy, childbirth or within 42 days of termination of pregnancy. Consideration as a near-miss case is irrespective of gestational age and includes women who have abortions or stillbirths. Criteria as follows:

        i. Organ dysfunction
        ii. Laparotomy (excluding cesarean section, including hysterectomy)
        iii. Referral to intensive care unit
        iv. Blood transfusion (any units) combined with severe anemia, severe postpartum hemorrhage, antepartum hemorrhage, or placental abruption
        v. Severe postpartum hemorrhage (≥1000 mL estimated blood loss or procedure including hysterectomy and blood transfusion)
        vi. Preeclampsia with severe features/eclampsia
        vii. Uterine rupture
        viii. Sepsis

3. Maternal anemia: Low hemoglobin levels throughout pregnancy and labor and delivery, classified as mild (10-10.9 g/dL), moderate (7-9.9 g/dL), or severe (<7 g/dL). Low hemoglobin levels in the postpartum period, classified as mild (11-11.9 g/dL), moderate (8-10.9 g/dL), or severe (<8 g/dL).

4. Stillbirth: Delivery of a fetus showing no signs of life, as indicated by absence of breathing, heartbeat, pulsation of the umbilical cord, or definite movements of voluntary muscles. Defined as death prior to delivery of a fetus at ≥20 weeks of gestation (or

>350g weight, if gestational age is unavailable). Additionally, we will analyze time-specific definitions:

a. Early stillbirth: 20-27 weeks,
b. Late stillbirth: 28-36 weeks
c. Term stillbirth: ≥37 weeks
d. WHO-defined stillbirth: ≥28 weeks.

5. Neonatal mortality: Death of a liveborn baby during the first 28 days of life from any cause.

6. Low birthweight: Birth weight <2500 g as assessed at delivery or within 72 hours for home births. Very low birth weight if <1500 g.

7. Preterm birth: Delivery prior to 37 completed weeks of gestation of a liveborn infant. Further classified as: extremely preterm (<28 weeks), very preterm (28-32 weeks), and moderate to late preterm (32-37 weeks). Gestational age at birth will be determined by the best obstetric estimate: last menstrual period, ultrasound, and the American College of Obstetricians and Gynecologists algorithm. Preterm delivery is separately defined as delivery prior to 37 completed weeks of gestation (live or stillbirth).

8. Small for gestational age: Birth weight of an infant of lower than the sex-specific 3rd and 10th percentile for each week of gestational age per INTERGROWTH standards. Combined gestational age information and birthweight will be used to further categorize into preterm-small-for-gestational-age (SGA), preterm-appropriate-for-gestational-age (AGA), term-SGA, and term-AGA.

Secondary Outcomes

9. Late maternal mortality: Death from any cause related to or aggravated by pregnancy or its management (excluding accidental or incidental causes), irrespective of the duration and site of the pregnancy, from 42 days postpartum up to one year.

10. Preeclampsia: Hypertensive disorder of pregnancy defined for those without preexisting hypertension as gestational hypertension AND gestational proteinuria OR clinical diagnosis of preeclampsia OR development of severe features (even in the absence of proteinuria). Defined for those with chronic hypertension as incident gestational proteinuria OR clinical diagnosis of preeclampsia OR development of severe features. Further defined as early-onset preeclampsia if <34 weeks gestation and late-onset if ≥34 weeks. Sub definitions as follows:

a. *Chronic (preexisting) hypertension*: ≥140/90 mm Hg before pregnancy or <20th week of gestation or use of antihypertensive medication before pregnancy OR history of hypertension OR reported taking antihypertensive medications.

b. *Gestational hypertension*: A systolic blood pressure ≥140 mm Hg on two occasions at least 1 hour apart or diastolic blood pressure ≥90 mm Hg on two occasions at least 1 hour apart during pregnancy.

c. *Gestational proteinuria*: 24-hour urine collection >300 mg protein OR single voided urine protein/creatinine ratio ≥0.3 OR dipstick reading of 1+ or 2+ (use only if other quantitative methods are not available).

d. *Severe features* (indicated by presence of one or more of the following):

   i. *Severe gestational hypertension*: during pregnancy defined as systolic blood pressure >160 mmHg or a diastolic blood pressure >110 mmHg on two occasions at least 1 minute apart after 20 weeks gestation; or at the time of delivery defined as one systolic blood pressure >160 mmHg or a diastolic blood pressure >110 mmHg among participants without chronic hypertension >160/110 (even in absence of proteinuria)

   ii. *Hepatic abnormality*: impaired liver function not accounted for by another diagnosis and characterized by serum transaminase concentration ≥2 times the upper limit of the normal range

   iii. *Severe persistent right upper quadrant or epigastric pain*: unresponsive to medication and not accounted for by an alternative diagnosis

   iv. *New onset neurological symptoms*: such as headache (unresponsive to medication and not accounted for by alternative diagnoses) or visual symptoms

   v. *Thrombocytopenia*: <100,000 platelets/microL

   vi. *Renal insufficiency*: defined as serum creatinine >1.1 mg/dL or a doubling of the serum creatinine concentration in the absence of other renal disease

   vii. *Pulmonary edema and/or eclampsia*: clinical report of new-onset tonic-clonic, focal, or multifocal seizures in the absence of other causative conditions such as epilepsy, cerebral arterial ischemia and infarction, intracranial hemorrhage, or drug use

   viii. *Evidence of end organ dysfunction*: by laboratory criteria

   ix. *Clinical diagnosis of HELLP syndrome*: based upon the presence of ALL of the laboratory abnormalities (hemolysis, elevated liver enzymes, and low platelet count)

      1. Hemolysis (established by at least two of the following): peripheral smear with schistocytes and burr cells, serum bilirubin ≥1.2 mg/dL (20.52 umol/L), low serum haptoglobin (≤25 mg/dL) or lactate

dehydrogenase (LDH) ≥2 times the upper level of normal (based on laboratory-specific reference ranges), severe anemia (unrelated to blood loss), and

    2. Elevated blood concentrations of liver transaminases (aspartate aminotransferase (AST) or alanine aminotransferase (ALT) to ≥2 times normal concentration (based on laboratory-specific reference ranges)

    3. Low platelets or thrombocytopenia <100 x 109/L)

11. Gestational hypertension: A systolic blood pressure ≥140 mm Hg on two occasions at least 1 hour apart or diastolic blood pressure≥90 mm Hg on two occasions at least 1 hour apart or severe gestational hypertension in pregnancy (not at time of delivery) defined as systolic blood pressure > 160 mmHg or a diastolic blood pressure >110 mmHg on two occasions at least 1 minute apart after 20 weeks gestation or severe gestational hypertension at the time of delivery defined as one systolic blood pressure > 160 mmHg or a diastolic blood pressure >110 mmHg among participants without chronic hypertension. Chronic hypertension defined by a clinical diagnosis of chronic hypertension (with or without need for medication) or systolic blood pressure >140 mm Hg on two occasions at least1 hour apart or diastolic blood pressure >90 mm Hg on two occasions at least 1 hour apart before 20 weeks' gestation OR medication used OR prior clinical diagnosis. Medications for hypertension include magnesium sulfate, hydralazine, methyldopa (aldomet), atenolol, nifedipine, betamethasone, labetalol, and dexamethasone.

12. Postpartum hypertension: Systolic blood pressure ≥140 mm Hg or diastolic blood pressure ≥90 mm Hg on two occasions at least 1 hour apart in the postpartum period.

13. Preterm birth indication: Delivery prior to 37 completed weeks of gestation of a birth (live or stillbirth), classified into spontaneous preterm or provider-initiated preterm. Spontaneous preterm is defined as delivery <37 weeks that occurs either secondary to preterm labor or preterm premature rupture of membranes. Provider-initiated preterm defined as a medical or obstetric complication or other reason that the health care provider initiates delivery at <37 completed weeks gestation.

14. Preterm premature rupture of membranes: Rupture of membranes before the onset of labor occurring before <37 weeks of gestation or clinical diagnosis of premature rupture of membranes.

15. Gestational diabetes: Diabetes first diagnosed during pregnancy and characterized by insulin resistance and high blood sugar. Clinical diagnosis or diagnosis by 75g oral glucose tolerance test (OGTT) around 28 weeks based on the International Association of the Diabetes and Pregnancy Study Groups thresholds: fasting > 5.1 mmol/L or 1-hr OGTT >10.0 mmol/L or 2-hr OGTT >8.5 mmol/L.

16. Maternal infection and sepsis: Defined as organ dysfunction (per WHO near-miss definition) resulting from infection during pregnancy, delivery, and over 42 days postpartum. Categories of infection are defined based on severity, as defined by the Global Maternal Sepsis Study:

    a. *Infection-related severe maternal outcomes*: women presenting with WHO near-miss criteria to define organ system dysfunction and maternal death

    b. *Infections with complications*: women with an invasive procedure to treat the source of infection (vacuum aspiration, dilatation and curettage, wound debridement, drainage, laparotomy and lavage, other surgery), or admission to ICU, or transfer to another facility

    c. *Less severe infections*: all other infections.

17. Perinatal depression: Defined as a clinical mood disorder during pregnancy and up to 42 days postpartum. Symptoms screened using the Edinburgh Postnatal Depression Scale. Threshold for defining depression will be country specific based on local validation studies: Kenya ≥13; Ghana: ≥11; Pakistan ≥14; India (Vellore): ≥8; India (Hodal) ≥10; Zambia ≥10.

18. Fetal death: A product of human conception, irrespective of the duration of the pregnancy, which, after expulsion or extraction, does not breath or show any other evidence of life such as beating of the heart, pulsation of the umbilical cord, or definite movement of voluntary muscles, whether or not the umbilical cord has been cut or the placenta is attached.

19. Infant mortality: Death of a neonate or an infant from delivery up to one year of life.

20. Cause of neonatal mortality: Causes of neonatal death based on verbal autopsy.

21. Timing of neonatal mortality: Death of a liveborn during the first 28 days of life. Neonatal deaths are classified by timing: <24 hours (i.e. first 24 hours of life), early neonatal mortality (i.e. first 7 days), or late neonatal mortality (i.e. between 7 and 28 days).

22. Cause of stillbirth: Causes of stillbirth based on verbal autopsy.

23. Timing of stillbirth: Delivery of a fetus showing no signs of life, as indicated by absence of breathing, heartbeat, pulsation of the umbilical cord, or definite movements of voluntary muscles. Death occurring prior to delivery of a fetus at ≥20 weeks of gestation (or >350 g weight, if gestational age is unavailable). . Antepartum stillbirth, defined as heart rate not detected ≥12 hrs prior to delivery and/or signs of maceration. Intrapartum stillbirth, defined as heart rate detected <12 hrs prior to delivery and/or no signs of maceration.

24. Hyperbilirubinemia: Defined as the presence of excess bilirubin during the first week of life (assessed at birth, 3 days, and 7 days of life). Outcome based on: continuous bilirubin by age (hours), proportion >75th percentile, and proportion >95th percentile.

25. Neonatal sepsis: Defined as inflammatory response and organ dysfunction following presence of a severe infection from delivery to 28 days as suspected (by a clinician) or proven (with culture). Further classified as early onset (i.e. delivery to 72 hours) or late onset (72 hours to 28 days).

26. Possible severe bacterial infection: Presence of any of the following clinical signs or symptoms as defined by the WHO IMCI criteria, which are consistent with possible severe bacterial infection, at the time of delivery to 59 days: not able to feed at all or not feeding well, convulsions, severe chest indrawing, high body temperature (≥38°C), low body temperature (less than 35.5°C), no movement/only when stimulated, and/or fast breathing (≥60 BPM) in infants less than 7 days old.

27. Postnatal weight trajectory: Continuous weight change from birth through one month of age. Trajectory is determined by percent body weight loss by 72 hours, time to regain birth weight, and continuous body weight.

28. Infant growth: Proportion of infants more than 2 standard deviations below the WHO Children Health Reference Growth Standard for: weight for age (underweight), length for age (stunting), length for weight (wasting), and/or head circumference for age (microcephaly).

Tertiary Outcomes

29. Placental disorders: Placental abruption is defined as the complete or partial separation of the placenta from the inner wall of the uterus before delivery. Signs include vaginal bleeding (painful or painless), pain during abdominal palpitations, and/or fetal distress. Diagnosis made via ultrasound. Placenta previa is defined at the partial or complete covering of the cervix by the placenta. Signs include vaginal bleeding without pain in the second or third trimester. Diagnosis made via ultrasound.

30. Uterine rupture: Uterine rupture is defined as the spontaneous tearing of the uterus prior to delivery that may result in the fetus being expelled into the peritoneal cavity. Symptoms and signs of uterine rupture include fetal bradycardia, variable decelerations, evidence of hypovolemia, loss of fetal station (detected during cervical examination), severe or constant abdominal pain, and cessation of uterine contractions, and/or recession of the presenting part.

31. Prolonged labor: Defined as labor lasting ≥24 hours.

32. Unplanned surgery: The occurrence of medically indicated or emergent surgical procedure during labor and delivery. Hysterectomy is defined as the removal of the uterus (i.e. partial) or of the uterus and the cervix (i.e. total hysterectomy). Cesarean section is defined as a surgical procedure used to deliver a baby through incisions in the abdomen and uterus.

33. Hemorrhage:

a. *Antepartum hemorrhage*: Bleeding from or into the genital tract, occurring from ≥24 weeks gestation and prior to delivery.

b. *Postpartum hemorrhage*: Defined as estimated blood loss of 500 mL or more within 24 hours after birth or clinician diagnosis.

c. *Severe postpartum hemorrhage*: perceived abnormal bleeding (1000 ml or more) or any bleeding with hypotension or blood transfusion.

34. Perinatal birth asphyxia: Clinician-reported failure to breathe spontaneously in the first minute after delivery.
