# Supplementary material for: Pregnancy Risk, Infant Surveillance, and Measurement Alliance (PRISMA) Maternal and Newborn Health Study: protocol for a multisite, prospective, open cohort study of pregnancy and postpartum health outcomes in South Asia and sub-Saharan Africa
Source: BMJ Open. 2026 Jan 20;16(1):e104512. doi: 10.1136/bmjopen-2025-104512 (PMC12820853; doi:10.1136/bmjopen-2025-104512)
Supplement: Author's manuscript [file bmjopen-2025-104512.draft_revisions.pdf]

BMJ Open is committed to open peer review. As part of this commitment we make the peer review history of every article we publish publicly available.

When an article is published we post the peer reviewers' comments and the authors' responses online. We also post the versions of the paper that were used during peer review. These are the versions that the peer review comments apply to.

The versions of the paper that follow are the versions that were submitted during the peer review process. They are not the versions of record or the final published versions. They should not be cited or distributed as the published version of this manuscript.

BMJ Open is an open access journal and the full, final, typeset and author-corrected version of record of the manuscript is available on our site with no access controls, subscription charges or pay-per-view fees (http://bmjopen.bmj.com).

If you have any questions on BMJ Open's open peer review process please email info.bmjopen@bmj.com

BMJ Open

Protocol for the Pregnancy Risk, Infant Surveillance, and Measurement Alliance (PRISMA) Maternal and Newborn Health Study: A multi-site, prospective, open cohort study of pregnancy and postpartum health outcomes in South Asia and sub-Saharan Africa

Journal:	*BMJ Open*
Manuscript ID	bmjopen-2025-104512
Article Type:	Protocol
Date Submitted by the Author:	01-May-2025

Complete List of Authors:	Maternal and Newborn Health Study Investigators, PRISMA; The George Washington University, Department of Global Health<br>Smith, Emily; The George Washington University, Department of Global Health

Keywords:	Pregnancy, Postpartum Period, Maternal medicine < OBSTETRICS, Protocols & guidelines < HEALTH SERVICES ADMINISTRATION & MANAGEMENT, Fetal medicine < OBSTETRICS

SCHOLARONE™
Manuscripts

TITLE

Protocol for the Pregnancy Risk, Infant Surveillance, and Measurement Alliance (PRISMA) Maternal and Newborn Health Study: A multi-site, prospective, open cohort study of pregnancy and postpartum health outcomes in South Asia and sub-Saharan Africa

CORRESPONDING AUTHOR

Emily R. Smith

The George Washington University Milken Institute School of Public Health

950 New Hampshire Ave NW #2, Washington, DC 20037

emilysmith@gwu.edu

AUTHORS

PRISMA (Pregnancy Risk, Infant Surveillance, and Measurement Alliance) Maternal and Newborn Health Study Investigators

Emily R. Smith, Christopher Mores, Qing Pan, Jennifer Seager, Sasha G. Baumann, Jaime Marquis, Christopher R. Sudfeld, Zahra Hoodbhoy, Imran Nisar, Fyezah Jehan, Aneeta Hotwani, Nida Yazdani, Amna Khan, Farzana Shaheen, Kinza Farooqui, Leena Chatterjee, Arjun Dang, Manavi Dang, R Venketeshwar, Sarmila Mazumder, Neeraj Sharma, Arun Singh Jadaun, Rupa Talukdar, Blair J. Wylie, Victor Akelo, Winnie K. Mwebia, Joyce Were, Dickson Gethi, Zacchaeus Abaja, Gregory Ouma, Harun Owuor, Kephas Otieno, Anne George Cherian, Santosh Joseph Benjamin, Venkata Raghava Mohan, Balakrishnan Vijayalekshmi, Sunitha Varghese, Jasmine Sugirtha, Daniel Jebakumar, James A, Margaret P. Kasaro, M. Bridget Spelke, Wilbroad Mutale, Felistas Mbewe, Humphrey Mwape, Bethany Freeman, Bellington Vwalika, Mutale Sampa, Kwaku Poku Asante, Sam Newton, Charlotte Tawiah Agyemang, Irene Apewe Adjei, Veronica Agyemang, Dennis Adu-Gyasi, Eliezer Odei-Lartey, Stephaney Gyaase

For peer review only

ABSTRACT

Introduction: Maternal and child mortality has markedly decreased worldwide over the past few decades. Despite this success, the decline remains unequal across countries and is overall insufficient to meet the Sustainable Development Goals. South Asia and sub-Saharan Africa bear most of the burden of maternal and child morbidity and mortality. Major gaps persist in our understanding of the causes, timing, diagnostic thresholds, and risk factors for adverse outcomes in these regions. Addressing these gaps requires new ways to prevent and treat disease, from novel diagnostics to precision public health strategies, all of which rely on high-quality clinical data from diverse populations. The Pregnancy Risk, Infant Surveillance, and Measurement Alliance (PRISMA) Maternal and Newborn Health Study aims to estimate population-level prevalence of morbidities and mortality and to assess biological, clinical, and sociodemographic risk among mother-infant pairs in India, Pakistan, Kenya, Ghana, and Zambia.

Methods and analysis: This study is a prospective, open cohort study with a planned recruitment of about 6,000 women annually across six research sites in five countries. Participants are pregnant women enrolled less than 20 weeks gestation, as determined by ultrasound, identified through active house-to-house and facility-based surveillance. Robust clinical data will be collected at up to 12 study visits during antenatal care, labor and delivery, and through one year postpartum. A total of 34 outcomes will be captured. The primary analysis will estimate the burden of adverse outcomes and examine associated risk factors to inform future intervention strategies. Data will also be used to develop normative values for pregnant and postpartum women, as well as predictive models to assess pregnancy risk.

Ethics and dissemination: PRISMA received institutional and national ethical approvals. Findings will be published in peer-reviewed open-access journals and disseminated at national and international forums to inform clinical guidelines and public health practice.

For peer review only - http://bmjopen.bmj.com/site/about/guidelines.xhtml

Strengths and limitations:

- Harmonized, multidimensional data collection on social, biological, clinical, and environmental factors related to health and disease.
- Large, community-based sample of an estimated 18,000 pregnant women and 15,300 live births over the first three years of the study.
- Use of combined cutting-edge machine learning algorithms and predictive modeling approaches to better understand and predict risks.
- Morbidity and mortality estimates may not be fully generalizable to other settings.

For peer review only

For peer review only - http://bmjopen.bmj.com/site/about/guidelines.xhtml

INTRODUCTION

Globally, the maternal mortality rate has declined by more than 43% since 1990 [1]. Despite this success, progress remains unequal between regions, with low- and middle-income countries (LMICs) bearing excess burden [2]. Southern Asia and sub-Saharan Africa account for 86% of all maternal deaths and have some of the world's highest maternal mortality rates at 134 (80% uncertainty interval (UI) 118 to 155) and 545 (UI 477 to 654) per 100,000 live births, for each region respectively [3]. The World Health Organization estimates that between 2003 and 2009 the top causes of death in both regions were hemorrhage, hypertensive disorders, and sepsis [4]. These three causes were responsible for over half of all maternal deaths. Indirect causes—such as HIV, malaria, tuberculosis, iron deficiency anemia, or diabetes mellitus—accounted for another 27.5% of maternal deaths [4]. However, due to challenges in event reporting and linking hospital records to national health surveillance systems, comprehensive data on the causes of maternal deaths and near-misses are lacking [5–8].

Pregnancy-related and pre-existing conditions are a growing concern, not only given their potential to be life-threatening, but also due to the lasting effects they have on maternal and infant health and quality of life [9,10]. It is well evidenced that maternal morbidities put women at risk of future complications and impact an estimated 4% to 8% of all deliveries [11]. However, the true burden of maternal morbidities remains unclear, as these conditions frequently go undiagnosed or undocumented, most existing research focuses on a single sequela, and few large clinical cohorts have been conducted in LMICs. For example, a review of 59 studies (only four of which were conducted in Southeast Asia and sub-Saharan Africa) found that hypertensive disorders of pregnancy more than doubled the risk of ischemic heart disease and heart failure later in life [12]. Similarly, gestational diabetes and maternal anemia have been identified as risk factors for long-term cardiovascular diseases [13,14]. Another systematic review of 24 studies in high-income countries found that postpartum hemorrhage was linked to persistent physical, psychological, and

For peer review only - http://bmjopen.bmj.com/site/about/guidelines.xhtml

psychosocial problems, including mastitis, post-traumatic stress disorder, depression, and delayed mother-infant bonding [15]. The narrow clinical and geographic focus of available research limits our understanding of the full range of risks women face during and after pregnancy, and how this varies across health settings. Multidimensional research that assesses biological, clinical, demographic, and social determinants of health is likely to improve risk prediction, thereby preventing maternal morbidities and reducing their enduring repercussions.

Infant health and development also suffer the consequences of maternal morbidities [10,16]. Data from a World Health Organization Global Survey of 172,461 singleton deliveries in 22 LMICs found that preeclampsia and pre-gestational diabetes significantly increased odds of provider-initiated and spontaneous preterm birth [17]. Preeclampsia and gestational diabetes have been connected to heightened risk of life-long adverse outcomes among offspring, including increased susceptibility to hypertension, kidney disease, nutritional deficiencies, cardiometabolic events, obesity, attention deficit hyperactivity disorder, and autism spectrum disorder [18–21]. Maternal infections and poor nutrition during pregnancy have been linked to suboptimal cognitive development for children [22,23]. To provide newborns with the best start to a healthy life, improvements in antenatal care are essential to appropriately manage pre-existing conditions and screen for risk factors. Ultimately, addressing maternal morbidities not only benefits women's health but further supports the long-term well-being of their children.

While early identification of morbidities and risk for adverse outcomes in the antenatal and postnatal periods is essential, correct diagnoses and risk prediction are hindered by the lack of empirical data underlying clinical guidance and risk stratification algorithms. Often these are predominantly derived from data collected among non-pregnant adults in limited geographies, most commonly the United States and China, due to concentrated investments in healthcare research and extensive electronic health systems [24–27]. Data disparities limit the generalizability and predictive accuracy when applied to peripartum women [25]. The rise of

For peer review only

machine learning has further highlighted the issue, as generated predictive algorithms typically rely on datasets from high-income settings, restricting their applicability to LMICs [24]. High quality, representative data is required to inform clinical guidance and risk prediction tools for maternal morbidities; better data is needed to inform normative ranges for perinatal depressive symptoms, blood pressure, biomarkers for hypertensive disorders of pregnancy, and hemoglobin levels [28–30]. Addressing these evidence gaps by generating multidimensional global datasets for use in clinical benchmarking is critical to enabling accurate, equitable care for all women.

Study rationale and objectives

Robust data on pregnancy risks—including medical history, clinical symptoms and diagnoses, behavioral and lifestyle factors, social and economic characteristics, environmental exposures, and nutritional status—are essential to developing strategies to effectively manage pregnancy risks and improve outcomes in resource-constrained environments [31,32]. Population-based, multidimensional, longitudinal data of mother-infant pairs is severely lacking, particularly from LMICs. This hinders our ability to estimate the prevalence of morbidities and mortality, and to attribute their various risk factors among pregnant women and infants during the antenatal and postnatal periods [31]. We further lack the detailed, individual patient data needed to develop advanced diagnostic tools and risk prediction algorithms, as well as adequate baseline data needed to effectively monitor for adverse events in the context of introducing new devices, drugs, and vaccines. Existing datasets have significant limitations in study designs or the completeness and accuracy of data collected. Often, data are self-reported, cross-sectional, and most datasets are unable to be pooled in a harmonized way. These limitations make it difficult to achieve the robust datasets needed for analysis and for prioritizing investments in research, development, and policy or guideline changes.

We designed the Pregnancy Risk, Infant Surveillance, and Measurement Alliance (PRISMA) Maternal and Newborn Health Study in response to these critical gaps. At a high level, the study

For peer review only

For peer review only - http://bmjopen.bmj.com/site/about/guidelines.xhtml

aims to evaluate pregnancy risk factors and their associations with adverse outcomes, including stillbirth, infant mortality and morbidity, and maternal mortality and morbidity. The resulting harmonized dataset will be used to estimate population-level disease and outcome burden in two South Asian and three sub-Saharan African countries, which will be evaluated to identify any overlooked health concerns in each study area. Our data will inform subsequent research to understand priority issues and to test innovative strategies that optimize pregnancy outcomes, including defining more representative and gestational-age-specific reference values for select biomarkers. The primary objectives are as follows: (1) To provide population-based prevalence estimates of key maternal and infant health outcomes, which may inform the development of future diagnostics and interventions; (2) To improve the global understanding of key risk factors for morbidity and mortality among pregnant women, their fetuses, and mother-infant pairs for up to one year postpartum; and (3) To collect data to enable the application of novel analytical techniques to create risk prediction tools.

METHODS AND ANALYSIS

Study design

The PRISMA Maternal and Newborn Health Study is an international, multisite, prospective, open-cohort study. Leveraging population-based and facility-based pregnancy surveillance systems, we will identify, screen, and enroll pregnant participants prior to 20 weeks gestation as confirmed by ultrasound. Detailed clinical data will be collected to characterize the health status of women and their fetuses and infants, including ultrasound imaging, laboratory testing, physical examination, validated health surveys, medical history by maternal recall, and facility record abstraction. Data collection will continue during the antepartum, intrapartum, and postpartum periods up to one year following delivery. Participants will be assessed at <20, 20, 28, 32, and 36 weeks gestation, at labor and delivery, at three days postpartum, and one, four, six, 26, and 52 weeks postpartum. Liveborn infants will be similarly assessed at birth, day three of life, and one,

For peer review only - http://bmjopen.bmj.com/site/about/guidelines.xhtml

four, six, 26, and 52 weeks old. In the event of a miscarriage or stillbirth, participants will be followed up through six weeks and one year, respectively. Verbal autopsies will be conducted in the case of maternal, fetal, neonatal, or infant death to determine cause. The study protocol will be implemented uniformly across sites to ensure that all laboratory and diagnostics tests, primary health outcomes, and related risk factors are measured correctly and consistently.

Study outcomes

We aim to capture data on 34 adverse health outcomes (**Table 1**), which are defined in **Supplement 1**. Outcomes were selected based on high-priority research gaps within study contexts and determined via an interactive protocol development process with input from study investigators and other subject matter experts. Feasibility was also considered when selecting the measurement method. Clinical definitions, measurements, and constructed variables for each health outcome are standardized across study sites.

Table 1. Study outcomes by population and level

Outcome Level	Population	
	**Gravida/Puerperium**	**Fetus/Infant**
Primary	(1) Maternal mortality<br>(2) Composite severe maternal outcomes<br>(3) Maternal anemia	(4) Stillbirth<br>(5) Neonatal mortality<br>(6) Low birthweight<br>(7) Preterm birth<br>(8) Small-for-gestational-age
Secondary	(9) Late maternal mortality<br>(10) Preeclampsia<br>(11) Gestational hypertension<br>(12) Postpartum hypertension<br>(13) Medical indication for preterm delivery<br>(14) Preterm premature rupture of membranes<br>(15) Gestational diabetes<br>(16) Maternal infection and sepsis<br>(17) Perinatal depression	(18) Fetal death<br>(19) Infant mortality<br>(20) Cause of neonatal mortality<br>(21) Timing of neonatal mortality<br>(22) Cause of stillbirth<br>(23) Timing of stillbirth<br>(24) Hyperbilirubinemia<br>(25) Neonatal sepsis<br>(26) Possible severe bacterial infection<br>(27) Postnatal weight trajectory<br>(28) Infant growth
Tertiary	(29) Placental disorders<br>(30) Uterine rupture	(34) Perinatal birth asphyxia

For peer review only

For peer review only - http://bmjopen.bmj.com/site/about/guidelines.xhtml

(31) Prolonged labor
(32) Unplanned surgery
(33) Antepartum and postpartum hemorrhage

Study settings

Data collection will occur in six study areas: Kisumu and Siaya, Kenya; Kintampo, Ghana; Lusaka, Zambia; Vellore, India; Hodal, India; and Rehri Goth and Ibrahim Hyderi, Karachi, Pakistan. Protocol implementation is carried out by teams at the Kenya Medical Research Institute, Kintampo Health Research Centre, University of North Carolina Global Projects Zambia, Christian Medical College Vellore, Society for Applied Studies, and Aga Khan University. Researchers at the George Washington University are responsible for protocol harmonization and central data coordination. Partnering institutions and investigators were appointed by the Gates Foundation based on existing research infrastructure and expertise. Each country team identified and mapped geographic areas in which to conduct the study. Estimates of the study area population size are described in **Table 2**.

Table 2. Estimates of study area population size, pregnancies, and live births

Study Area	No. Households	No. Women of Reproductive Age	Total Population Size	Anticipated No. Pregnancies (Live Births)[1]
Kintampo, Ghana	34,640	45,767	177,200	3,000 (2,250)
Vellore, India	54, 435	47,443	234,624	2,700 (2,300)
Hodal, India	36,321	35,499	196,678	2,000 (1,700)
Kisumu, Kenya	44,932	47,826	171,698	3,000 (2,550)
Karachi, Pakistan	23,170	34,682	227,549	6,000 (5,100)
Lusaka, Zambia	31,165	34,942	145,263	2,250 (1,914)
**All sites**	**224,663**	**246,159**	**1,153,012**	**18,550 (15,814)**

[1]*Anticipated number of pregnancies based off expected annual enrollment over three years; expected 85% of pregnancies to result in a live birth*

Eligibility criteria

Women who meet the following criteria may be eligible for enrollment: lives within the study

For peer review only

For peer review only - http://bmjopen.bmj.com/site/about/guidelines.xhtml

catchment area with no plans to permanently relocate during pregnancy or within one year postpartum; meets minimum age requirement for consenting in country of residence (Ghana: 15 years of age; Kenya: 18 years of age or emancipated minors; Pakistan: 15 years of age or emancipated minors; Zambia: 15 years of age; India: 18 years of age); and viable intrauterine pregnancy less than 20 weeks gestation determined by ultrasound. Women who have more than one pregnancy during the study period are eligible for re-enrollment. Multiple gestations (i.e., two, three, or more fetuses) will also be eligible.

Pregnancy surveillance

Household-based and facility-based surveillance systems will be used to identify women of reproductive age with suspected pregnancies. Routine health and demographic surveillance systems, either existing or established for the purpose of this study, will be leveraged to visit homes in the study catchment area at least once every six months. Women identified at these visits will be invited to provide basic socio-demographic information as a part of the population-based surveillance and screened for study eligibility. Women that receive antenatal care at participating health facilities will also be identified and screened. Combined, these women make up the sampling frame for the study cohort.

Participant identification and enrollment

A multiphase process of pre-screening, consenting, and screening will be used to enroll participants (**Figure 1**). Identified women of reproductive age will be prescreened for potential eligibility by community fieldworkers based on the following criteria: reported symptoms or clinical signs of pregnancy; estimated gestational age per last menstrual period <25 weeks; lives within the study catchment area; and meets minimum age requirement in each study site. Any woman potentially eligible after pre-screening will be invited to provide informed consent or assent in her preferred language. Certified sonographers will then conduct an ultrasound to verify that the woman has an intrauterine pregnancy and is prior to 20 weeks gestation. Women who are beyond

For peer review only - http://bmjopen.bmj.com/site/about/guidelines.xhtml

For peer review only

the gestational age threshold or with a non-viable pregnancy (e.g., ectopic) are not eligible to participate and will be referred.

Figure 1. Overview of surveillance and identification of potentially eligible women

Study follow-up procedures

Follow-up is planned to occur through one year postpartum for participants with liveborn and stillborn infants, and through six weeks for participants with spontaneous or induced abortions. Data will be collected at up to 12 study visits scheduled according to gestational age. This includes five antenatal study visits at <20 weeks (enrollment), 20 weeks, 28 weeks, 32 weeks, and 36 weeks gestation. Data will also be collected for labor and delivery within 24 hours for facility births and 72 hours for home births. Deliveries will be observed by study staff, when possible, otherwise data will be abstracted from medical records and by maternal or provider recall. After delivery, there will be six postnatal visits at three days, one week, four weeks, six weeks, 26 weeks, and 52 weeks postpartum for both mothers and infants. For each scheduled study visit there is an accepted visit window. If the accepted window has passed and no visit occurred, the visit will be considered missed, forms submitted with any available information, and the subsequent visit scheduled. In addition to scheduled visits, any unscheduled follow-ups or hospital visits during the study period will be documented.

Harmonized data collection

Trained study staff will use harmonized measurement procedures and data collection tools developed for the purpose of this study [33]. Each tool corresponds to a scheduled or ad hoc study visit conducted either at a study facility or at home and contains information on approximately 4,000 core variables (**Table 3**). For maternal participants, the scope of data collected includes sociodemographic characteristics, health behaviors, environmental exposures, medical and obstetric history, anthropometry, vital and clinical status, current physical and mental

For peer review only - http://bmjopen.bmj.com/site/about/guidelines.xhtml

health state, medications and treatments, vaccination status, laboratory tests, ultrasounds, and diagnostic assessments. As participants may deliver outside of the study facility, data capturing clinical status and events during labor and delivery will be collected via a combination of observation, retrospective self-report or report by birth attendant, and/or abstraction from medical records. For infant participants, data collected during postnatal care visits includes anthropometry, signs and symptoms of infection, clinical and vital signs, vaccination status, medications and treatments, and cognitive development assessments.

Table 3. Type of data collected at scheduled study visits by timepoint in weeks

**Type of Data Collected**	**Scheduled Study Timepoint[1]**											
	**Antenatal (weeks)**					**Deliv.**	**Postnatal (weeks)**					
	**<20**	**20**	**28**	**32**	**36**		**<1**	**1**	**4**	**6**	**26**	**52**
**Maternal**												
Prescreening	✓											
Ultrasound	✓	O	O	✓	O							
Enrollment	✓											
Sociodemographics	✓											
Clinical status	✓	✓	✓	✓	✓	✓	✓	✓	✓	✓	✓	✓
Anthropometry	✓	✓	✓	✓	✓	⧫				✓	✓	✓
Vital signs	✓	✓	✓	✓	✓	✓	✓	✓	✓	✓	✓	✓
Lab tests	✓	✓	✓	✓	✓	O	✓	✓	✓	✓	✓	✓
Depression[2]		✓		✓						✓		
Quality of life[3]				✓					✓			
Delivery outcome						✓						
Quality of care					O	O						O

For peer review only

For peer review only - http://bmjopen.bmj.com/site/about/guidelines.xhtml

Study close-out					**		✓
**Infant**							
Birth outcome	✓						
Apgar score	✓						
Anthropometry	⧫	✓	✓	✓	✓	✓	✓
Jaundice screen[4]	⧫	✓	✓				
Vital signs[5]		✓	✓	✓	✓	✓	✓
Clinical status		✓	✓	✓	✓	✓	✓
Lab tests					✓	✓	✓
Vaccination		✓	✓	✓	✓	✓	✓
Cognitive development[6]							✓
Stimulation[7]							O
Study close-out							✓

✓Required
OOptional
⧫Only collected if delivery occurred in study facility
***Close-out occurs at 6 weeks in the case of spontaneous or induced abortion*
[1]Additional ad hoc forms are administered in the event of hospitalization, severe adverse events, protocol deviations, and death (verbal autopsy)
[2]Depressive symptoms measured by an adapted version of the Edinburgh Postnatal Depression Scale
[3]Measured by the 12-Item Short Form Health Survey (SF-12) and World Health Organization Disability Assessment Schedule (WHODAS 2.0)
[4]Measured by Mennen Medical Bili-Care transcutaneous bilirubin device and Bili-ruler visual ichometer
[5]Heart rate and oxygen saturation
[6]Measured by the Global Scale for Early Development Short Form (version 1.0)
[7]Measured by the Family Care Indicators assessment

Laboratory and diagnostic tests

Maternal and infant participants will undergo study-specific laboratory and diagnostic testing at scheduled visits (**Table 4**). Wherever possible and with consideration to participant burden, gold standard methods will be used. At all antenatal visits, hemoglobin levels will be assessed and routine urinalysis done by dipstick. In addition to these assessments, each study contact point has specific requirements.

For peer review only - http://bmjopen.bmj.com/site/about/guidelines.xhtml

During enrollment, pregnancy viability and fetal biometry will be determined via ultrasound for gestational age dating and to verify study eligibility. If eligible, blood specimens will be collected to test for Rh factor, hemoglobin A1c levels, micronutrient deficiencies, inflammatory markers, liver and renal function, soluble fms-like tyrosine kinase-1, and placental growth factor. Rapid diagnostic tests will be conducted for HIV, malaria, syphilis, and hepatitis B and C infection. If the syphilis rapid test is positive, it will be followed up with a nontreponemal test to check syphilis titers. If clinically indicated by the World Health Organization Four-Symptom Screen, a tuberculosis test will also be done [34].

At the 28-week antenatal visit, an oral glucose tolerance test will be done to screen for gestational diabetes among participants with normal hemoglobin A1c levels at enrollment. Soluble fms-like tyrosine kinase-1 and placental growth factor biomarker testing will also be repeated at this time. A repeat ultrasound to measure fetal biometry is recommended at 28 weeks and required at 32 weeks. At the 32-week antenatal visit, blood tests will also be repeated for micronutrient deficiencies, inflammatory markers, and thyroid function. If the syphilis rapid test is negative at enrollment, it will be repeated. If the rapid test is positive, it will be followed up with a nontreponemal test to check syphilis titers. At the 36-week antenatal visit, the rapid diagnostic tests for HIV, malaria, syphilis, and tuberculosis infection are repeated as clinically indicated or locally recommended.

At all postnatal visits, maternal hemoglobin levels will be assessed. At the six-week postnatal visit, an additional maternal blood specimen will be collected to test for micronutrient deficiencies and inflammation. For participants who tested positive for gestational diabetes during antenatal care, an oral glucose tolerance test will be done at the six-week visit along with a hemoglobin A1c test at the final 52-week visit.

For peer review only - http://bmjopen.bmj.com/site/about/guidelines.xhtml

Further testing among a subsample or time-limited testing of specific infections or biomarkers will be incorporated at the discretion of the PRISMA consortium to better understand novel findings or previously undetected health concerns. Beginning in November 2023, sites asynchronously rolled out vaginal swab testing for chlamydia and gonorrhea at the enrollment and 32-week antenatal care visits, which will continue for one year of data collection. Among a subsample of 1,000 participants per site, testing for differential diagnosis of febrile illnesses—including hepatitis E, Zika, chikungunya, dengue, and leptospirosis infection—will be administered at enrollment, 32 weeks gestation, and six weeks postpartum. Depending on the identified burden of disease, investigators may choose to continue testing.

Table 4. Maternal laboratory and diagnostic tests during antenatal and postnatal care

Assessment	Scheduled Study Visit										
	**Antenatal care (weeks)**					**Postnatal care (weeks)**					
	**<20**	**20**	**28**	**32**	**36**	**<1**	**1**	**4**	**6**	**26**	**52**
Hemoglobin levels	✓	✓	✓	✓	✓	✓	✓	✓	✓	✓	✓
Urinalysis	✓	✓	✓	✓	✓						
Rh factor	✓										
Hemoglobin A1c	✓										*
Oral glucose tolerance			✓[1]						*		
Micronutrient deficiencies[2]	✓			✓					✓		
Inflammation biomarkers[3]	✓			✓					✓		
Preeclampsia biomarkers[4]	✓		✓	✓							
Liver function	✓										
Renal function	✓										
Thyroid function				✓							
Syphilis infection	✓			✓	*						

For peer review only

For peer review only - http://bmjopen.bmj.com/site/about/guidelines.xhtml

Syphilis titers	*	*	
Chlamydia infection	+	+	
Gonorrhea infection	+	+	
HIV infection	✓	*	
Malaria infection	✓	*	
Tuberculosis infection	*	*	
Hepatitis B infection	✓		
Hepatitis C infection	✓		
Hepatitis E infection	+	+	+
Zika infection	+	+	+
Chikungunya infection	+	+	+
Dengue infection	+	+	+
Leptospirosis	+	+	+

✓Required
**As clinically indicated*
+Testing completed for a subset of participants at each site, following which investigators will decide whether or not to continue with these tests based on the observed burden of disease
[1]Only among women with normal HbA1c at enrollment
[2]Vitamin B12 total cobalamin, folate, vitamin A retinol binding protein, iodine thyroglobulin, iron serum transferrin receptor, and iron ferritin
[3]C-reactive protein and alpha 1-acid glycoprotein
[4]Soluble fms-like tyrosine kinase-1 and placental growth factor

Infants similarly will undergo a limited set of laboratory and diagnostic tests at scheduled time points (**Table 5**). At birth, transcutaneous bilirubin assessment is done within 72 hours (24 hours preferred). Transcutaneous bilirubin assessments will be repeated at the three-to-five-day visit (<1 week) and the one-week visit. Hemoglobin levels will be assessed by HemoCue at the six-week, six-month, and one-year visits. Tests for malaria infection will be performed at six months and one year. Infants will be tested for HIV and hepatitis B tests only in the case of maternal infection.

For peer review only

Table 5. Infant laboratory and diagnostic tests at birth and during postnatal care

Assessment	Scheduled Study Visit						
	Birth	Postnatal (weeks)					
		<1	1	4	6	26	52
Bilirubin levels[1]	✓	✓	✓				
Hemoglobin levels					✓	✓	✓
Malaria infection						✓	✓
HIV infection					*	*	*
Hepatitis B virus							*

✓Required
**In the case of maternal infection*
[1]Bilirubin measured via transcutaneous bilirubinometer and Bili-ruler visual ichometer

Quality control and assurance

All procedures will adhere to Quality Assurance and Quality Control Standard Operating Procedures that were collaboratively developed for this study. Central training was conducted for designated study staff members from each site to ensure harmonized procedures for ultrasound examinations, verbal autopsies, and assessment for integrated management of childhood illnesses. Personnel taking anthropometric measurements received study-specific training and will participate in ongoing standardization exercises at regular intervals. Calibration checks of circumference tapes, scales, stadiometers, and infantometers for anthropometric assessments will be performed monthly. An external monitoring group has been contracted to ensure that the rights and safety of study subjects are protected; to ensure the quality and integrity of study data; and to ensure the study is conducted in compliance with the approved protocol and applicable regulatory requirements.

A proportion of ultrasound images will undergo external quality assessment, and all fetal biometry measures will undergo plausibility checks. Sonographers at all sites will be certified in study-

For peer review only

For peer review only - http://bmjopen.bmj.com/site/about/guidelines.xhtml

specific procedures and undergo annual refresher training to ensure compliance. We will use Tricefy™ for secure cloud medical imaging storage and sharing of anonymized data. Anonymized images will be randomly peer-reviewed and expert-reviewed; summary quality reports will then be generated monthly.

Biospecimen collection and laboratory testing will be conducted according to Standard Operating Procedures. Laboratories implementing micronutrient measurements on site will be enrolled in the Centers for Disease Control Performance Verification Program for Serum Micronutrients. For full blood count measurement, all participating laboratories will be enrolled in the United Kingdom National External Quality Assessment Scheme for Hematology. For full blood count and all other analytes, laboratories must also be enrolled in the College of American Pathologists' external quality assurance program.

Data quality monitoring

To ensure the success of the study, a baseline phase was carried out in 2021 to pilot the performance of study tools and procedures. Performance was evaluated using both qualitative and quantitative methods. At the end of the pilot period, a harmonization process to standardize tools and data systems across study sites was implemented.

Obtaining high-quality data is central to the study; thus, data quality monitoring is a key component of standard operating procedures. Each country team will be responsible for the initial data cleaning, including running of routine range and consistency checks as well as periodic reviews of distributions and identification of outliers. Any inconsistencies within their database will be resolved in consultation with the clinical and field data collection team. A cleaned, limited dataset from all sites will be stored in a secure central data repository cloud server, shared at bi-weekly intervals. Data analysts on the coordination team will run additional data quality checks and produce both data queries and monitoring reports using pooled data on at least a bi-monthly

For peer review only - http://bmjopen.bmj.com/site/about/guidelines.xhtml

basis. Data queries will identify issues related to the following: range checks, skip patterns, consistency logic, date and time concordance, submitted records to the database, uniqueness of a record (i.e., de-duplicating), outliers, digit preference, and missingness. Monitoring reports will include details such as rate of enrollment, protocol compliance, completeness of key clinical and lab assessments, and the distribution of key health outcomes. Data management teams will share codes for data checks via GitHub repositories.

Data management systems and security

Data collection instruments were developed by each country's team. All systems are encrypted to ensure no subject information is readily accessible. Signed consent forms and study data collection forms will be stored in a secure location with access limited to authorized research staff. A limited study dataset, which will not contain any protected health information or personally identifiable information beyond complete birth and death dates, will be maintained in a secure central repository. This dataset may be shared with current and future research partners or affiliates for analysis purposes through a data sharing agreement. Any partners given permission to access the data will be required to adhere to the Gates Foundation Global Access policies, which require that any knowledge and information gained or any products developed must be made available and accessible at an affordable price to the people most in need living in LMICs.

Patient and public involvement

Each implementing institution has its own community advisory board. During initial protocol development, investigators evaluated the additional burden of study participation on patients; for this reason, invasive clinical assessments are kept to a minimum and grounded in evidence-based care guidelines. Patients' experiences were centered during the translation and validation process for questionnaires to measure postpartum depression and maternal morbidity symptoms. When selecting the primary and secondary outcomes, the prevalence and impact of each outcome within study site contexts was strongly considered. Upon completion of the study, we

For peer review only

intend to share the main findings with participants and community members via appropriate local dissemination methods. Finally, we will ensure that participants' contributions to this research are acknowledged in any subsequent reports, presentations, and publications.

ETHICS AND DISSEMINATION

Ethical and safety considerations

This protocol, the informed consent documents, and any subsequent modifications will be reviewed and approved by the relevant institutional review board (IRB) and ethics review committee (ERC) responsible for oversight of the study at each site. Approvals were received from the following IRBs and ERCs in each country: Ghana (Ghana Health Service ERC: 019/09/20; Kintampo Health Research Centre Institutional Ethics Committee: KHRCIEC/2020-17); India (Office of Research, Christian Medical College, Vellore, India: IRB14553; Ethics Review Committee, Society for Applied Studies, New Delhi: SAS/ERC/ReMAPPStudy/2022; Kenya (KEMRI Scientific and Ethics Review Unit: KEMRI/SERU/4166; Liverpool School of Tropical Medicine Ethics Committee: 23-020 Jaramogi Oginga Odinga Teaching and Referral Hospital (JOOTRH) Institutional Scientific Ethics Review Committee ISERC: ISERC/JOOTRH/549/2024); Pakistan (The Aga Khan University ERC: No. 2023-9100-26333; Pakistan National Institutes of Health—Health Research Institute, National Bioethics Committee: 4-87/NBCR-1023/23/973); Zambia (University of North Carolina Chapel Hill Office of Human Research Ethics: Study No. 14-2113; University of Zambia Biomedical Research Ethics Committee: 016-04-14); and the United States (The George Washington University IRB: NCR224396; Columbia University IRB: IRB-AAAU7504; Harvard University IRB: IRB23-1093; The University of Alabama at Birmingham IRB: IRB-300013081). All pregnant women will be provided with information about the study and the option to opt-out of data collection. For women who are not of legal age for consent, assent together with parental or other legal consent must be obtained per local standards. Study participants will be referred to higher-level or specialty care according to site-specific standard

For peer review only - http://bmjopen.bmj.com/site/about/guidelines.xhtml

operating procedures.

Dissemination plan

The data generated in the course of the study will be reviewed on an ongoing basis, according to priorities set by the investigators. Quarterly and annual reports will be shared with key stakeholders, as well as the funder (Gates Foundation). Interim findings with potential implications for informing public health policy or practice will be shared with major local and international stakeholders, including the World Health Organization, United States Centers for Disease Control, United States Agency for International Development, and Ministries of Health in each participating country. Local meetings will be organized to share the findings of this study with community members and study participants at each site. Abstracts from both country-specific and pooled global data analyses will be developed for dissemination through both local and international scientific conferences and publication in peer-reviewed journals.

AUTHORS' CONTRIBUTIONS

This study is the result of an international collaborative effort carried out by a large group of institutions and members of the PRISMA Consortium. Conceptualization: ERS, ZH, IN, SM, NS, ASJ, BJW, VA, WKM, DG, AGC, SJB, VRM, MPK, MBS, WM, KPA, SN, and CTA. Funding acquisition: ERS, CM, ZH, IN, SM, VA, AGC, SJB, MPK, MBS, WM, KPA, SN, and CTA. Methodology: ERS, CM, QP, JS, CRS, ZH, IN, FJ, SM, NS, ASJ, BJW, VA, WKM, DG, AGC, SJB, VRM, MPK, MBS, WM, BF, KPA, SN, and CTA. Project administration: SGB, JM, AH, NY, AK, FS, KF, NS, ASJ, JW, ZA, GO, HO, SV, JS, DJ, J, FM, HM, BF, BV, MS, IAA, VA, EO-L, and SG. Resources: CM, AH, LC, AD, MD, RV, KO, BV, HM, VA, and DAG. Supervision: ERS, CM, QP, JS, CRS, ZH, IN, FJ, AH, SM, RT, BJW, VA, WKM, DG, KO, AGC, SJB, VRM, MPK, MBS, WM, BF, BV, KPA, SN, and CTA. Writing—original draft: ERS, SGB, and CRS. Writing—review & editing: ERS, CM, QP, JS, SGB, JM, CRS, ZH, IN, FJ, AH, NY, AK, FS, KF, LC, AD, MD, RV,

For peer review only - http://bmjopen.bmj.com/site/about/guidelines.xhtml

SM, NS, ASJ, RT, BJW, VA, WKM, JW, DG, ZA, GO, HO, KO, AGC, SJB, VRM, BV, SV, JS, DJ, J, MPK, MBS, WM, FM, BF, BV, MS, KPA, SN, CTA, IAA, VA, DAG, EO-L, and SG.

FUNDING STATEMENT

This work is supported by the Gates Foundation grant number [INV-047400 to KPA, CTA, and SN; INV-057219 to VA; INV-043092 to SB; INV-005776 to VPT; INV-057220 to ZH; INV-016221 to MPK; K01TW012426 NIH/FIC to MBS; INV-057222 to WM; INV-041999 to ERS; INV-060797 to CM; and INV-057223 to SM].

ACKNOWLEDGEMENTS

The Maternal and Newborn Health Study is a research project of the Pregnancy Risk, Infant Surveillance, and Measurement Alliance (PRISMA) Consortium. PRISMA members are grateful to the extensive network of individuals and organizations who contributed to the design of this study, including but not limited to biostatisticians, laboratory technicians, obstetricians, community health workers, field data collectors, and research partners at the Institute for Health Metrics and Evaluation, Centers for Disease Control and Prevention, and the World Health Organization. The authors also acknowledge the efforts of study participants and community members, without whom this would not be possible.

COMPETING INTERESTS STATEMENT

The authors have no competing interests to declare.

REFERENCES

1. Alkema L, Chou D, Hogan D, Zhang S, Moller AB, Gemmill A, et al. Global, regional, and national levels and trends in maternal mortality between 1990 and 2015, with scenario-based projections to 2030: a systematic analysis by the UN Maternal Mortality Estimation Inter-Agency Group. Lancet. 2016 Jan 30;387(10017):462–74.

2. Who. Trends in maternal mortality 2000 to 2017: estimates by WHO, UNICEF, UNFPA, World Bank Group and the United Nations Population Division. World Health Organization Geneva, Switzerland; 2019.

For peer review only

456 3. WHO, UNICEF, UNFPA, World Bank Group and UNDESA/Population Division. Trends in maternal mortality 2000 to 2020 [Internet]. World Health Organization; 2023. Report No.: ISBN 978-92-4-006875-9. Available from: https://iris.who.int/bitstream/handle/10665/366225/9789240068759-eng.pdf?sequence=1

460 4. Say L, Chou D, Gemmill A, Tunçalp Ö, Moller AB, Daniels J, et al. Global causes of maternal death: a WHO systematic analysis. Lancet Glob Health. 2014 Jun;2(6):e323–33.

462 5. Musarandega R, Nyakura M, Machekano R, Pattinson R, Munjanja SP. Causes of maternal mortality in Sub-Saharan Africa: A systematic review of studies published from 2015 to 2020. J Glob Health. 2021 Oct 9;11:04048.

465 6. Hussein J. Non-communicable diseases during pregnancy in low and middle income countries. Obstet Med. 2017 Mar;10(1):26–9.

467 7. Firoz T, Pineles B, Navrange N, Grimshaw A, Oladapo O, Chou D. Non-communicable diseases and maternal health: a scoping review. BMC Pregnancy Childbirth. 2022 Oct 22;22(1):787.

470 8. O'Neil S, Taylor S, Sivasankaran A. Data equity to advance health and health equity in low- and middle-income countries: A scoping review. Digit Health. 2021 Jan 22;7:20552076211061922.

473 9. Souza JP, Day LT, Rezende-Gomes AC, Zhang J, Mori R, Baguiya A, et al. A global analysis of the determinants of maternal health and transitions in maternal mortality. Lancet Glob Health. 2024 Feb 1;12(2):e306–16.

476 10. Geller SE, Koch AR, Garland CE, MacDonald EJ, Storey F, Lawton B. A global view of severe maternal morbidity: moving beyond maternal mortality. Reprod Health. 2018 Jun 22;15(Suppl 1):98.

479 11. Lori S. Ashford, Elizabeth Ransom, Nancy Yinger. Hidden Suffering: Disabilities From Pregnancy and Childbirth in Less Developed Countries [Internet]. PRB. 2002 [cited 2024 Dec 3]. Available from: https://www.prb.org/resources/hidden-suffering-disabilities-from-pregnancy-and-childbirth-in-less-developed-countries/

483 12. Sukmanee J, Liabsuetrakul T. Risk of future cardiovascular diseases in different years postpartum after hypertensive disorders of pregnancy: A systematic review and meta-analysis. Medicine (Baltimore). 2022 Jul 29;101(30):e29646.

486 13. Azulay CE, Pariente G, Shoham-Vardi I, Kessous R, Sergienko R, Sheiner E. Maternal anemia during pregnancy and subsequent risk for cardiovascular disease. J Matern Fetal Neonatal Med. 2015 Oct 13;28(15):1762–5.

489 14. Li J, Song C, Li C, Liu P, Sun Z, Yang X. Increased risk of cardiovascular disease in women with prior gestational diabetes: A systematic review and meta-analysis. Diabetes Res Clin Pract. 2018 Jun 1;140:324–38.

492 15. Latt SM, Alderdice F, Elkington M, Awng Shar M, Kurinczuk JJ, Rowe R. Primary postpartum haemorrhage and longer-term physical, psychological, and psychosocial health outcomes for women and their partners in high income countries: A mixed-methods systematic review. PLoS One. 2023 Jun 14;18(6):e0274041.

For peer review only - http://bmjopen.bmj.com/site/about/guidelines.xhtml

16. Lawn JE, Ohuma EO, Bradley E, Idueta LS, Hazel E, Okwaraji YB, et al. Small babies, big risks: global estimates of prevalence and mortality for vulnerable newborns to accelerate change and improve counting. Lancet. 2023 May 20;401(10389):1707–19.

17. Vogel JP, Lee ACC, Souza JP. Maternal morbidity and preterm birth in 22 low- and middle-income countries: a secondary analysis of the WHO Global Survey dataset. BMC Pregnancy Childbirth. 2014 Jan 31;14(1):56.

18. Korzeniewski SJ, Sutton E, Escudero C, Roberts JM. The Global Pregnancy Collaboration (CoLab) symposium on short- and long-term outcomes in offspring whose mothers had preeclampsia: A scoping review of clinical evidence. Front Med (Lausanne). 2022 Aug 30;9:984291.

19. Turbeville HR, Sasser JM. Preeclampsia beyond pregnancy: long-term consequences for mother and child. Am J Physiol Renal Physiol. 2020 Jun 1;318(6):F1315–26.

20. Fraser A, Lawlor DA. Long-term health outcomes in offspring born to women with diabetes in pregnancy. Curr Diab Rep. 2014 Mar 25;14(5):489.

21. Feig DS, Artani A, Asaf A, Li P, Booth GL, Shah BR. Long-term neurobehavioral and metabolic outcomes in offspring of mothers with diabetes during pregnancy: A large, population-based cohort study in Ontario, Canada. Diabetes Care. 2024 Sep 1;47(9):1568–75.

22. Hao X, Lu J, Yan S, Tao F, Huang K. Maternal pre-pregnancy body mass index, gestational weight gain and children's cognitive development: A Birth Cohort study. Nutrients [Internet]. 2022 Nov 1;14. Available from: https://www.mdpi.com/2072-6643/14/21/4613

23. Kwok J, Hall HA, Murray AL, Lombardo MV, Auyeung B. Maternal infections during pregnancy and child cognitive outcomes. BMC Pregnancy Childbirth. 2022 Nov 17;22(1):848.

24. Celi LA, Cellini J, Charpignon ML, Dee EC, Dernoncourt F, Eber R, et al. Sources of bias in artificial intelligence that perpetuate healthcare disparities-A global review. PLOS Digit Health. 2022 Mar;1(3):e0000022.

25. Hasan BS, Rasheed MA, Wahid A, Kumar RK, Zuhlke L. Generating evidence from contextual clinical research in Low- to Middle Income Countries: A roadmap based on theory of change. Front Pediatr. 2021 Dec 9;9:764239.

26. Bhakuni H, Abimbola S. Epistemic injustice in academic global health. Lancet Glob Health. 2021 Oct;9(10):e1465–70.

27. Acharya KP, Pathak S. Applied research in low-income countries: Why and how? Front Res Metr Anal. 2019 Nov 14;4:3.

28. Ruhighira JJ, Mashili FL, Tungu AM, Mamuya S. Spirometry profiles among pregnant and non-pregnant African women: a cross-sectional study. BMC Womens Health. 2022 Dec 2;22(1):483.

29. Goldstein RF, Abell SK, Ranasinha S, Misso ML, Boyle JA, Harrison CL, et al. Gestational weight gain across continents and ethnicity: systematic review and meta-analysis of

For peer review only - http://bmjopen.bmj.com/site/about/guidelines.xhtml

maternal and infant outcomes in more than one million women. BMC Med. 2018 Aug 31;16(1):153.

30. Bouthoorn SH, Gaillard R, Steegers EAP, Hofman A, Jaddoe VWV, van Lenthe FJ, et al. Ethnic differences in blood pressure and hypertensive complications during pregnancy: the Generation R study. Hypertension. 2012 Jul;60(1):198–205.

31. AMANHI (Alliance for Maternal and Newborn Health Improvement), Baqui A, Ahmed P, Dasgupta SK, Begum N, Rahman M, et al. Development and validation of a simplified algorithm for neonatal gestational age assessment - protocol for the Alliance for Maternal Newborn Health Improvement (AMANHI) prospective cohort study. J Glob Health. 2017 Dec;7(2):021201.

32. Goldenberg RL, Saleem S, Ali S, Moore JL, Lokangako A, Tshefu A, et al. Maternal near miss in low-resource areas. Int J Gynaecol Obstet. 2017 Sep;138(3):347–55.

33. Smith ER, Mores C, Tawiah C, Asante KP, Mazumder S, Cherian AG, et al. PRISMA Maternal & Newborn Health Study [Internet]. Open Science Foundation; 2024 [cited 2024 Nov 15]. Available from: https://osf.io/qckyt

34. World Health Organization. WHO operational handbook on tuberculosis [Internet]. Genève, Switzerland: World Health Organization; 2021. Available from: https://iris.who.int/bitstream/handle/10665/340256/9789240022614-eng.pdf

For peer review only

For peer review only - http://bmjopen.bmj.com/site/about/guidelines.xhtml

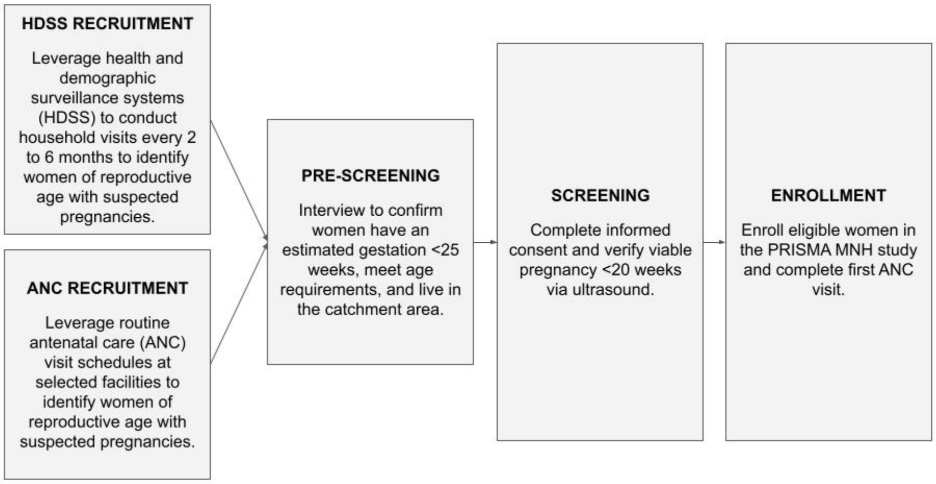

338x190mm (72 x 72 DPI)

SUPPLEMENT 1. OUTCOME DEFINITIONS

Primary Outcomes

1. Maternal mortality: Death from any cause related to or aggravated by pregnancy or its management (excluding accidental or incidental causes) during pregnancy and childbirth or within 42 days of termination of pregnancy, irrespective of the duration and site of the pregnancy.

2. Composite severe maternal outcomes: Composite outcome comprising maternal deaths, late maternal deaths, and maternal near-misses, each defined as the following:

   a. *Maternal death*: Death from any cause related to or aggravated by pregnancy or its management (excluding accidental or incidental causes) during pregnancy and childbirth or within 42 days of termination of pregnancy, irrespective of the duration and site of the pregnancy.

   b. *Late maternal death*: Death from any cause related to or aggravated by pregnancy or its management (excluding accidental or incidental causes), irrespective of the duration and site of the pregnancy, from 42 days postpartum up to one year.

   c. *Maternal near-miss*: Woman who nearly died but survived a life-threatening condition that occurred during pregnancy, childbirth or within 42 days of termination of pregnancy. Consideration as a near-miss case is irrespective of gestational age and includes women who have abortions or stillbirths. Criteria as follows:

      i. Organ dysfunction
      ii. Laparotomy (excluding cesarean section, including hysterectomy)
      iii. Referral to intensive care unit
      iv. Blood transfusion (any units) combined with severe anemia, severe postpartum hemorrhage, antepartum hemorrhage, or placental abruption
      v. Severe postpartum hemorrhage (≥1000 mL estimated blood loss or procedure including hysterectomy and blood transfusion)
      vi. Preeclampsia with severe features/eclampsia
      vii. Uterine rupture
      viii. Sepsis

3. Maternal anemia: Low hemoglobin levels throughout pregnancy and labor and delivery, classified as mild (10-10.9 g/dL), moderate (7-9.9 g/dL), or severe (<7 g/dL). Low hemoglobin levels in the postpartum period, classified as mild (11-11.9 g/dL), moderate (8-10.9 g/dL), or severe (<8 g/dL).

4. Stillbirth: Delivery of a fetus showing no signs of life, as indicated by absence of breathing, heartbeat, pulsation of the umbilical cord, or definite movements of voluntary muscles. Defined as death prior to delivery of a fetus at ≥20 weeks of gestation (or

For peer review only

>350g weight, if gestational age is unavailable). Additionally, we will analyze time-specific definitions:

a. Early stillbirth: 20-27 weeks,
b. Late stillbirth: 28-36 weeks
c. Term stillbirth: ≥37 weeks
d. WHO-defined stillbirth: ≥28 weeks.

5. Neonatal mortality: Death of a liveborn baby during the first 28 days of life from any cause.

6. Low birthweight: Birth weight <2500 g as assessed at delivery or within 72 hours for home births. Very low birth weight if <1500 g.

7. Preterm birth: Delivery prior to 37 completed weeks of gestation of a liveborn infant. Further classified as: extremely preterm (<28 weeks), very preterm (28-32 weeks), and moderate to late preterm (32-37 weeks). Gestational age at birth will be determined by the best obstetric estimate: last menstrual period, ultrasound, and the American College of Obstetricians and Gynecologists algorithm. Preterm delivery is separately defined as delivery prior to 37 completed weeks of gestation (live or stillbirth).

8. Small for gestational age: Birth weight of an infant of lower than the sex-specific 3rd and 10th percentile for each week of gestational age per INTERGROWTH standards. Combined gestational age information and birthweight will be used to further categorize into preterm-small-for-gestational-age (SGA), preterm-appropriate-for-gestational-age (AGA), term-SGA, and term-AGA.

Secondary Outcomes

9. Late maternal mortality: Death from any cause related to or aggravated by pregnancy or its management (excluding accidental or incidental causes), irrespective of the duration and site of the pregnancy, from 42 days postpartum up to one year.

10. Preeclampsia: Hypertensive disorder of pregnancy defined for those without preexisting hypertension as gestational hypertension AND gestational proteinuria OR clinical diagnosis of preeclampsia OR development of severe features (even in the absence of proteinuria). Defined for those with chronic hypertension as incident gestational proteinuria OR clinical diagnosis of preeclampsia OR development of severe features. Further defined as early-onset preeclampsia if <34 weeks gestation and late-onset if ≥34 weeks. Sub definitions as follows:

a. *Chronic (preexisting) hypertension*: ≥140/90 mm Hg before pregnancy or <20th week of gestation or use of antihypertensive medication before pregnancy OR history of hypertension OR reported taking antihypertensive medications.

For peer review only

b. *Gestational hypertension*: A systolic blood pressure ≥140 mm Hg on two occasions at least 1 hour apart or diastolic blood pressure ≥90 mm Hg on two occasions at least 1 hour apart during pregnancy.

c. *Gestational proteinuria*: 24-hour urine collection >300 mg protein OR single voided urine protein/creatinine ratio ≥0.3 OR dipstick reading of 1+ or 2+ (use only if other quantitative methods are not available).

d. *Severe features* (indicated by presence of one or more of the following):

i. *Severe gestational hypertension*: during pregnancy defined as systolic blood pressure >160 mmHg or a diastolic blood pressure >110 mmHg on two occasions at least 1 minute apart after 20 weeks gestation; or at the time of delivery defined as one systolic blood pressure >160 mmHg or a diastolic blood pressure >110 mmHg among participants without chronic hypertension >160/110 (even in absence of proteinuria)

ii. *Hepatic abnormality*: impaired liver function not accounted for by another diagnosis and characterized by serum transaminase concentration ≥2 times the upper limit of the normal range

iii. *Severe persistent right upper quadrant or epigastric pain*: unresponsive to medication and not accounted for by an alternative diagnosis

iv. *New onset neurological symptoms*: such as headache (unresponsive to medication and not accounted for by alternative diagnoses) or visual symptoms

v. *Thrombocytopenia*: <100,000 platelets/microL

vi. *Renal insufficiency*: defined as serum creatinine >1.1 mg/dL or a doubling of the serum creatinine concentration in the absence of other renal disease

vii. *Pulmonary edema and/or eclampsia*: clinical report of new-onset tonic-clonic, focal, or multifocal seizures in the absence of other causative conditions such as epilepsy, cerebral arterial ischemia and infarction, intracranial hemorrhage, or drug use

viii. *Evidence of end organ dysfunction*: by laboratory criteria

ix. *Clinical diagnosis of HELLP syndrome*: based upon the presence of ALL of the laboratory abnormalities (hemolysis, elevated liver enzymes, and low platelet count)

1. Hemolysis (established by at least two of the following): peripheral smear with schistocytes and burr cells, serum bilirubin ≥1.2 mg/dL (20.52 umol/L), low serum haptoglobin (≤25 mg/dL) or lactate dehydrogenase (LDH) ≥2 times the upper level of normal (based on

For peer review only - http://bmjopen.bmj.com/site/about/guidelines.xhtml

laboratory-specific reference ranges), severe anemia (unrelated to blood loss), and

2. Elevated blood concentrations of liver transaminases (aspartate aminotransferase (AST) or alanine aminotransferase (ALT) to ≥2 times normal concentration (based on laboratory-specific reference ranges)

3. Low platelets or thrombocytopenia <100 x 109/L)

11. Gestational hypertension: A systolic blood pressure ≥140 mm Hg on two occasions at least 1 hour apart or diastolic blood pressure≥90 mm Hg on two occasions at least 1 hour apart or severe gestational hypertension in pregnancy (not at time of delivery) defined as systolic blood pressure > 160 mmHg or a diastolic blood pressure >110 mmHg on two occasions at least 1 minute apart after 20 weeks gestation or severe gestational hypertension at the time of delivery defined as one systolic blood pressure > 160 mmHg or a diastolic blood pressure >110 mmHg among participants without chronic hypertension. Chronic hypertension defined by a clinical diagnosis of chronic hypertension (with or without need for medication) or systolic blood pressure >140 mm Hg on two occasions at least1 hour apart or diastolic blood pressure >90 mm Hg on two occasions at least 1 hour apart before 20 weeks' gestation OR medication used OR prior clinical diagnosis. Medications for hypertension include magnesium sulfate, hydralazine, methyldopa (aldomet), atenolol, nifedipine, betamethasone, labetalol, and dexamethasone.

12. Postpartum hypertension: Systolic blood pressure ≥140 mm Hg or diastolic blood pressure ≥90 mm Hg on two occasions at least 1 hour apart in the postpartum period.

13. Preterm birth indication: Delivery prior to 37 completed weeks of gestation of a birth (live or stillbirth), classified into spontaneous preterm or provider-initiated preterm. Spontaneous preterm is defined as delivery <37 weeks that occurs either secondary to preterm labor or preterm premature rupture of membranes. Provider-initiated preterm defined as a medical or obstetric complication or other reason that the health care provider initiates delivery at <37 completed weeks gestation.

14. Preterm premature rupture of membranes: Rupture of membranes before the onset of labor occurring before <37 weeks of gestation or clinical diagnosis of premature rupture of membranes.

15. Gestational diabetes: Diabetes first diagnosed during pregnancy and characterized by insulin resistance and high blood sugar. Clinical diagnosis or diagnosis by 75g oral glucose tolerance test (OGTT) around 28 weeks based on the International Association of the Diabetes and Pregnancy Study Groups thresholds: fasting > 5.1 mmol/L or 1-hr OGTT >10.0 mmol/L or 2-hr OGTT >8.5 mmol/L.

16. Maternal infection and sepsis: Defined as organ dysfunction (per WHO near-miss definition) resulting from infection during pregnancy, delivery, and over 42 days

For peer review only

postpartum. Categories of infection are defined based on severity, as defined by the Global Maternal Sepsis Study:

a. *Infection-related severe maternal outcomes*: women presenting with WHO near-miss criteria to define organ system dysfunction and maternal death

b. *Infections with complications*: women with an invasive procedure to treat the source of infection (vacuum aspiration, dilatation and curettage, wound debridement, drainage, laparotomy and lavage, other surgery), or admission to ICU, or transfer to another facility

c. *Less severe infections*: all other infections.

17. Perinatal depression: Defined as a clinical mood disorder during pregnancy and up to 42 days postpartum. Symptoms screened using the Edinburgh Postnatal Depression Scale. Threshold for defining depression will be country specific based on local validation studies: Kenya ≥13; Ghana: ≥11; Pakistan ≥14; India (Vellore): ≥8; India (Hodal) ≥10; Zambia ≥10.

18. Fetal death: A product of human conception, irrespective of the duration of the pregnancy, which, after expulsion or extraction, does not breath or show any other evidence of life such as beating of the heart, pulsation of the umbilical cord, or definite movement of voluntary muscles, whether or not the umbilical cord has been cut or the placenta is attached.

19. Infant mortality: Death of a neonate or an infant from delivery up to one year of life.

20. Cause of neonatal mortality: Causes of neonatal death based on verbal autopsy.

21. Timing of neonatal mortality: Death of a liveborn during the first 28 days of life. Neonatal deaths are classified by timing: <24 hours (i.e. first 24 hours of life), early neonatal mortality (i.e. first 7 days), or late neonatal mortality (i.e. between 7 and 28 days).

22. Cause of stillbirth: Causes of stillbirth based on verbal autopsy.

23. Timing of stillbirth: Delivery of a fetus showing no signs of life, as indicated by absence of breathing, heartbeat, pulsation of the umbilical cord, or definite movements of voluntary muscles. Death occurring prior to delivery of a fetus at ≥20 weeks of gestation (or >350 g weight, if gestational age is unavailable). . Antepartum stillbirth, defined as heart rate not detected ≥12 hrs prior to delivery and/or signs of maceration. Intrapartum stillbirth, defined as heart rate detected <12 hrs prior to delivery and/or no signs of maceration.

24. Hyperbilirubinemia: Defined as the presence of excess bilirubin during the first week of life (assessed at birth, 3 days, and 7 days of life). Outcome based on: continuous bilirubin by age (hours), proportion >75th percentile, and proportion >95th percentile.

25. Neonatal sepsis: Defined as inflammatory response and organ dysfunction following presence of a severe infection from delivery to 28 days as suspected (by a clinician) or

For peer review only

proven (with culture). Further classified as early onset (i.e. delivery to 72 hours) or late onset (72 hours to 28 days).

26. Possible severe bacterial infection: Presence of any of the following clinical signs or symptoms as defined by the WHO IMCI criteria, which are consistent with possible severe bacterial infection, at the time of delivery to 59 days: not able to feed at all or not feeding well, convulsions, severe chest indrawing, high body temperature (≥38°C), low body temperature (less than 35.5°C), no movement/only when stimulated, and/or fast breathing (≥60 BPM) in infants less than 7 days old.

27. Postnatal weight trajectory: Continuous weight change from birth through one month of age. Trajectory is determined by percent body weight loss by 72 hours, time to regain birth weight, and continuous body weight.

28. Infant growth: Proportion of infants more than 2 standard deviations below the WHO Children Health Reference Growth Standard for: weight for age (underweight), length for age (stunting), length for weight (wasting), and/or head circumference for age (microcephaly).

Tertiary Outcomes

29. Placental disorders: Placental abruption is defined as the complete or partial separation of the placenta from the inner wall of the uterus before delivery. Signs include vaginal bleeding (painful or painless), pain during abdominal palpitations, and/or fetal distress. Diagnosis made via ultrasound. Placenta previa is defined at the partial or complete covering of the cervix by the placenta. Signs include vaginal bleeding without pain in the second or third trimester. Diagnosis made via ultrasound.

30. Uterine rupture: Uterine rupture is defined as the spontaneous tearing of the uterus prior to delivery that may result in the fetus being expelled into the peritoneal cavity. Symptoms and signs of uterine rupture include fetal bradycardia, variable decelerations, evidence of hypovolemia, loss of fetal station (detected during cervical examination), severe or constant abdominal pain, and cessation of uterine contractions, and/or recession of the presenting part.

31. Prolonged labor: Defined as labor lasting ≥24 hours.

32. Unplanned surgery: The occurrence of medically indicated or emergent surgical procedure during labor and delivery. Hysterectomy is defined as the removal of the uterus (i.e. partial) or of the uterus and the cervix (i.e. total hysterectomy). Cesarean section is defined as a surgical procedure used to deliver a baby through incisions in the abdomen and uterus.

33. Hemorrhage:

    a. *Antepartum hemorrhage*: Bleeding from or into the genital tract, occurring from ≥24 weeks gestation and prior to delivery.

For peer review only

b. *Postpartum hemorrhage*: Defined as estimated blood loss of 500 mL or more within 24 hours after birth or clinician diagnosis.

c. *Severe postpartum hemorrhage*: perceived abnormal bleeding (1000 ml or more) or any bleeding with hypotension or blood transfusion.

34. Perinatal birth asphyxia: Clinician-reported failure to breathe spontaneously in the first minute after delivery.

For peer review only

BMJ Open

Protocol for the Pregnancy Risk, Infant Surveillance, and Measurement Alliance (PRISMA) Maternal and Newborn Health Study: A multi-site, prospective, open cohort study of pregnancy and postpartum health outcomes in South Asia and sub-Saharan Africa

Journal:	*BMJ Open*
Manuscript ID	bmjopen-2025-104512.R1
Article Type:	Protocol
Date Submitted by the Author:	06-Oct-2025
Complete List of Authors:	Maternal and Newborn Health Study Investigators, PRISMA; The George Washington University, Department of Global Health<br>Smith, Emily; The George Washington University, Department of Global Health
**Primary Subject Heading**:	Obstetrics and gynaecology
Secondary Subject Heading:	Paediatrics, Global health, Epidemiology, Infectious diseases, Nutrition and metabolism
Keywords:	Pregnancy, Postpartum Period, Maternal medicine < OBSTETRICS, Protocols & guidelines < HEALTH SERVICES ADMINISTRATION & MANAGEMENT, Fetal medicine < OBSTETRICS

SCHOLARONE™
Manuscripts

TITLE

Protocol for the Pregnancy Risk, Infant Surveillance, and Measurement Alliance (PRISMA) Maternal and Newborn Health Study: A multi-site, prospective, open cohort study of pregnancy and postpartum health outcomes in South Asia and sub-Saharan Africa

CORRESPONDING AUTHOR

Emily R. Smith

The George Washington University Milken Institute School of Public Health

950 New Hampshire Ave NW #2, Washington, DC 20037

emilysmith@gwu.edu

AUTHORS

Pregnancy Risk, Infant Surveillance, and Measurement Alliance (PRISMA) Investigators

For peer review only

ABSTRACT

Introduction: Maternal and child mortality has markedly decreased worldwide over the past few decades. Despite this success, the decline remains unequal across countries and is overall insufficient to meet the Sustainable Development Goals. South Asia and sub-Saharan Africa bear most of the burden of maternal and child morbidity and mortality. Major gaps persist in our understanding of the causes, timing, diagnostic thresholds, and risk factors for adverse outcomes in these regions. Addressing these gaps requires new ways to prevent and treat disease, from novel diagnostics to precision public health strategies, all of which rely on high-quality clinical data from diverse populations. The Pregnancy Risk, Infant Surveillance, and Measurement Alliance (PRISMA) Maternal and Newborn Health Study aims to estimate population-level prevalence of morbidities and mortality and to assess biological, clinical, and sociodemographic risk among mother-infant pairs in India, Pakistan, Kenya, Ghana, and Zambia.

Methods and analysis: This study is a prospective, open cohort study with a planned recruitment of about 6,000 women annually across six research sites in five countries. Participants are pregnant women enrolled less than 20 weeks gestation, as determined by ultrasound, identified through active house-to-house and facility-based surveillance. Robust clinical data will be collected at 12 scheduled study visits during antenatal care, labor and delivery, and through one year postpartum. A total of 34 outcomes will be captured. The primary analysis will estimate the burden of adverse outcomes and examine associated risk factors to inform future intervention strategies. Data will also be used to develop normative values for pregnant and postpartum women, as well as predictive models to assess pregnancy risk.

Ethics and dissemination: PRISMA received institutional and national ethical approvals. Findings will be published in peer-reviewed open-access journals and disseminated at national and international forums to inform clinical guidelines and public health practice.

For peer review only - http://bmjopen.bmj.com/site/about/guidelines.xhtml

Strengths and limitations:

- Harmonized, multidimensional data collection on social, biological, clinical, and environmental factors related to health and disease.
- Large, community-based sample of an estimated 18,000 pregnant women and 15,300 live births over the first three years of the study.
- Use of combined cutting-edge machine learning algorithms and predictive modeling approaches to better understand and predict risks.
- Morbidity and mortality estimates may not be fully generalizable to other settings.

For peer review only

INTRODUCTION

Globally, the maternal mortality rate has declined by more than 43% since 1990 [1]. Despite this success, progress remains unequal between regions, with low- and middle-income countries (LMICs) bearing excess burden [2]. Southern Asia and sub-Saharan Africa account for 86% of all maternal deaths and have some of the world's highest maternal mortality rates at 134 (80% uncertainty interval (UI) 118 to 155) and 545 (UI 477 to 654) per 100,000 live births, for each region respectively [3]. The World Health Organization estimates that between 2003 and 2009 the top causes of death in both regions were hemorrhage, hypertensive disorders, and sepsis [4]. These three causes were responsible for over half of all maternal deaths. Indirect causes—such as HIV, malaria, tuberculosis, iron deficiency anemia, or diabetes mellitus—accounted for another 27.5% of maternal deaths [4]. However, due to challenges in event reporting and linking hospital records to national health surveillance systems, comprehensive data on the causes of maternal deaths and near-misses are lacking [5–8].

Pregnancy-related and pre-existing conditions are a growing concern, not only given their potential to be life-threatening, but also due to the lasting effects they have on maternal and infant health and quality of life [9,10]. It is well evidenced that maternal morbidities put women at risk of future complications and impact an estimated 4% to 8% of all deliveries [11]. However, the true burden of maternal morbidities remains unclear, as these conditions frequently go undiagnosed or undocumented, most existing research focuses on a single sequela, and few large clinical cohorts have been conducted in LMICs. For example, a review of 59 studies (only four of which were conducted in Southeast Asia and sub-Saharan Africa) found that hypertensive disorders of pregnancy more than doubled the risk of ischemic heart disease and heart failure later in life [12]. Similarly, gestational diabetes and maternal anemia have been identified as risk factors for long-term cardiovascular diseases [13,14]. Another systematic review of 24 studies in high-income countries found that postpartum hemorrhage was linked to persistent physical, psychological, and

For peer review only - http://bmjopen.bmj.com/site/about/guidelines.xhtml

psychosocial problems, including mastitis, post-traumatic stress disorder, depression, and delayed mother-infant bonding [15]. The narrow clinical and geographic focus of available research limits our understanding of the full range of risks women face during and after pregnancy, and how this varies across health settings. Multidimensional research that assesses biological, clinical, demographic, and social determinants of health is likely to improve risk prediction, thereby preventing maternal morbidities and reducing their enduring repercussions.

Infant health and development also suffer the consequences of maternal morbidities [10,16]. Data from a World Health Organization Global Survey of 172,461 singleton deliveries in 22 LMICs found that preeclampsia and pre-gestational diabetes significantly increased odds of provider-initiated and spontaneous preterm birth [17]. Preeclampsia and gestational diabetes have been connected to heightened risk of life-long adverse outcomes among offspring, including increased susceptibility to hypertension, kidney disease, nutritional deficiencies, cardiometabolic events, obesity, attention deficit hyperactivity disorder, and autism spectrum disorder [18–21]. Maternal infections and poor nutrition during pregnancy have been linked to suboptimal cognitive development for children [22,23]. To provide newborns with the best start to a healthy life, improvements in antenatal care are essential to appropriately manage pre-existing conditions and screen for risk factors. Ultimately, addressing maternal morbidities not only benefits women's health but further supports the long-term well-being of their children.

While early identification of morbidities and risk for adverse outcomes in the antenatal and postnatal periods is essential, correct diagnoses and risk prediction are hindered by the lack of empirical data underlying clinical guidance and risk stratification algorithms. Often these are predominantly derived from data collected among non-pregnant adults in limited geographies, most commonly the United States and China, due to concentrated investments in healthcare research and extensive electronic health systems [24–27]. Data disparities limit the generalizability and predictive accuracy when applied to peripartum women [25]. The rise of

For peer review only - http://bmjopen.bmj.com/site/about/guidelines.xhtml

machine learning has further highlighted the issue, as generated predictive algorithms typically rely on datasets from high-income settings, restricting their applicability to LMICs [24]. High quality, representative data is required to inform clinical guidance and risk prediction tools for maternal morbidities; better data is needed to inform normative ranges for perinatal depressive symptoms, blood pressure, biomarkers for hypertensive disorders of pregnancy, and hemoglobin levels [28–30]. Addressing these evidence gaps by generating multidimensional global datasets for use in clinical benchmarking is critical to enabling accurate, equitable care for all women.

Study rationale and objectives

Robust data on pregnancy risks—including medical history, clinical symptoms and diagnoses, behavioral and lifestyle factors, social and economic characteristics, environmental exposures, and nutritional status—are essential to developing strategies to effectively manage pregnancy risks and improve outcomes in resource-constrained environments [31,32]. Population-based, multidimensional, longitudinal data of mother-infant pairs is severely lacking, particularly from LMICs. This hinders our ability to estimate the prevalence of morbidities and mortality, and to attribute their various risk factors among pregnant women and infants during the antenatal and postnatal periods [31]. We further lack the detailed, individual patient data needed to develop advanced diagnostic tools and risk prediction algorithms, as well as adequate baseline data needed to effectively monitor for adverse events in the context of introducing new devices, drugs, and vaccines. Existing datasets have significant limitations in study designs or the completeness and accuracy of data collected. Often, data are self-reported, cross-sectional, and most datasets are unable to be pooled in a harmonized way. These limitations make it difficult to achieve the robust datasets needed for analysis and for prioritizing investments in research, development, and policy or guideline changes.

We designed the Pregnancy Risk, Infant Surveillance, and Measurement Alliance (PRISMA) Maternal and Newborn Health Study in response to these critical gaps. At a high level, the study

For peer review only - http://bmjopen.bmj.com/site/about/guidelines.xhtml

aims to evaluate pregnancy risk factors and their associations with adverse outcomes, including stillbirth, infant mortality and morbidity, and maternal mortality and morbidity. The resulting harmonized dataset will be used to estimate population-level disease and outcome burden in two South Asian and three sub-Saharan African countries, which will be evaluated to identify any overlooked health concerns in each study area. Our data will inform subsequent research to understand priority issues and to test innovative strategies that optimize pregnancy outcomes, including defining more representative and gestational-age-specific reference values for select biomarkers. The primary objectives are as follows: (1) To provide population-based prevalence estimates of key maternal and infant health outcomes, which may inform the development of future diagnostics and interventions; (2) To improve the global understanding of key risk factors for morbidity and mortality among pregnant women, their fetuses, and mother-infant pairs for up to one year postpartum; and (3) To collect data to enable the application of novel analytical techniques to create risk prediction tools.

METHODS AND ANALYSIS

Study design

The PRISMA Maternal and Newborn Health Study is an international, multisite, prospective, open-cohort study. Leveraging population-based and facility-based pregnancy surveillance systems, we will identify, screen, and enroll pregnant participants prior to 20 weeks gestation as confirmed by ultrasound. Enrollment began in September 2022 in Pakistan, November 2022 in Kenya, December 2022 in Ghana and Kenya, June 2023 in South India, and December 2023 in North India. Detailed clinical data will be collected to characterize the health status of women and their fetuses and infants, including ultrasound imaging, laboratory testing, physical examination, validated health surveys, medical history by maternal recall, and facility record abstraction. Data collection will continue during the antepartum, intrapartum, and postpartum periods up to one year following delivery. Participants will be assessed at scheduled visits at <20, 20, 28, 32, and 36

For peer review only - http://bmjopen.bmj.com/site/about/guidelines.xhtml

weeks gestation, at labor and delivery, at three days postpartum, and one, four, six, 26, and 52 weeks postpartum. Liveborn infants will be similarly assessed at birth, day three of life, and one, four, six, 26, and 52 weeks old. Any additional visits or hospitalizations, for example due to complications, will also be documented. In the event of a miscarriage or stillbirth, participants will be followed up through six weeks and one year, respectively. Verbal autopsies will be conducted in the case of maternal, fetal, neonatal, or infant death and analyzed by InterVA computer software to ensure a harmonized approach to cause of death determination. The study protocol will be implemented uniformly across sites to ensure that all laboratory and diagnostics tests, primary health outcomes, and related risk factors are measured correctly and consistently.

Study outcomes

We aim to capture data on 34 adverse health outcomes (**Table 1**), which are defined in **Supplement 1**. Outcomes were selected based on high-priority research gaps within study contexts and determined via an interactive protocol development process with input from study investigators and other subject matter experts. Feasibility was also considered when selecting the measurement method. Clinical definitions, measurements, and constructed variables for each health outcome are standardized across study sites.

Table 1. Study outcomes by population and level

Outcome Level	Population	
	**Gravida/Puerperium**	**Fetus/Infant**
Primary	(1) Maternal mortality<br>(2) Composite severe maternal outcomes<br>(3) Maternal anemia	(4) Stillbirth<br>(5) Neonatal mortality<br>(6) Low birthweight<br>(7) Preterm birth<br>(8) Small-for-gestational-age
Secondary	(9) Late maternal mortality<br>(10) Preeclampsia<br>(11) Gestational hypertension<br>(12) Postpartum hypertension<br>(13) Medical indication for preterm delivery	(18) Fetal death<br>(19) Infant mortality<br>(20) Cause of neonatal mortality<br>(21) Timing of neonatal mortality<br>(22) Cause of stillbirth

For peer review only

	(14) Preterm premature rupture of membranes<br>(15) Gestational diabetes<br>(16) Maternal infection and sepsis<br>(17) Perinatal depression	(23) Timing of stillbirth<br>(24) Hyperbilirubinemia<br>(25) Neonatal sepsis<br>(26) Possible severe bacterial infection<br>(27) Postnatal weight trajectory<br>(28) Infant growth
Tertiary	(29) Placental disorders<br>(30) Uterine rupture<br>(31) Prolonged labor<br>(32) Unplanned surgery<br>(33) Antepartum and postpartum hemorrhage	(34) Perinatal birth asphyxia

Study settings

Data collection will occur in six study areas: Kisumu and Siaya, Kenya; Kintampo, Ghana; Lusaka, Zambia; Vellore, India; Hodal, India; and Rehri Goth and Ibrahim Hyderi, Karachi, Pakistan. Protocol implementation is jointly carried out by clinical teams at participating health facilities and study staff from partnering research institutions (i.e. the Kenya Medical Research Institute, Kintampo Health Research Centre, University of North Carolina Global Projects Zambia, Christian Medical College Vellore, Society for Applied Studies, and Aga Khan University). Clinical assessments and subsequent findings conducted by study staff will be integrated with clinical care. Researchers at the George Washington University are responsible for protocol harmonization and central data coordination. Consortium institutions and investigators were appointed by the Gates Foundation based on existing research infrastructure and expertise. Each country team identified and mapped geographic areas in which to conduct the study. Estimates of the study area population size are described in **Table 2**.

For peer review only

Table 2. Estimates of study area population size, pregnancies, and live births

Study Area	No. Households	No. Women of Reproductive Age	Total Population Size	Anticipated No. Pregnancies (Live Births)[1]
Kintampo, Ghana	34,640	45,767	177,200	3,000 (2,250)
Vellore, India	54, 435	47,443	234,624	2,700 (2,300)
Hodal, India	36,321	35,499	196,678	2,000 (1,700)

For peer review only - http://bmjopen.bmj.com/site/about/guidelines.xhtml

Kisumu, Kenya	44,932	47,826	171,698	3,000 (2,550)
Karachi, Pakistan	23,170	34,682	227,549	6,000 (5,100)
Lusaka, Zambia	31,165	34,942	145,263	2,250 (1,914)
**All sites**	**224,663**	**246,159**	**1,153,012**	**18,550 (15,814)**

[1]*Anticipated number of pregnancies based off expected annual enrollment over three years; expected 85% of pregnancies to result in a live birth*

Eligibility criteria

Women who meet the following criteria may be eligible for enrollment: lives within the study catchment area with no plans to permanently relocate during pregnancy or within one year postpartum; meets minimum age requirement for consenting in country of residence (Ghana: 15 years of age; Kenya: 18 years of age or emancipated minors; Pakistan: 15 years of age or emancipated minors; Zambia: 15 years of age; India: 18 years of age); and viable intrauterine pregnancy less than 20 weeks gestation determined by ultrasound. Women who have more than one pregnancy during the study period are eligible for re-enrollment. Multiple gestations (i.e., two, three, or more fetuses) will also be eligible.

Pregnancy surveillance

Household-based and facility-based surveillance systems will be used to identify women of reproductive age with suspected pregnancies. Routine health and demographic surveillance systems, either existing or established for the purpose of this study, will be leveraged to visit homes in the study catchment area at least once every six months. Women identified at these visits will be invited to provide basic socio-demographic information as a part of the population-based surveillance and screened for study eligibility. Women that receive antenatal care at participating health facilities will also be identified and screened. Combined, these women make up the sampling frame for the study cohort.

For peer review only

For peer review only - http://bmjopen.bmj.com/site/about/guidelines.xhtml

Participant identification and enrollment

A multiphase process of pre-screening, consenting, and screening will be used to enroll participants (**Figure 1**). Identified women of reproductive age will be prescreened for potential eligibility by community fieldworkers based on the following criteria: reported symptoms or clinical signs of pregnancy; estimated gestational age per last menstrual period <25 weeks; lives within the study catchment area; and meets minimum age requirement in each study site. Any woman potentially eligible after pre-screening will be invited to provide informed consent or assent in her preferred language. Certified sonographers will then conduct an ultrasound to verify that the woman has an intrauterine pregnancy and is prior to 20 weeks gestation. Women who are beyond the gestational age threshold or with a non-viable pregnancy (e.g., ectopic) are not eligible to participate and will be referred.

Figure 1. Overview of surveillance and identification of potentially eligible women

Study follow-up procedures

Follow-up is planned to occur through one year postpartum for participants with liveborn and stillborn infants, and through six weeks for participants with spontaneous or induced abortions. Data will be collected at 12 study visits scheduled according to gestational age. This includes five antenatal study visits at <20 weeks (enrollment), 20 weeks, 28 weeks, 32 weeks, and 36 weeks gestation. Data will also be collected for labor and delivery within 24 hours for facility births and 72 hours for home births. Deliveries will be observed by study staff, when possible, otherwise data will be abstracted from medical records and by maternal or provider recall. After delivery, there will be six postnatal visits at three days, one week, four weeks, six weeks, 26 weeks, and 52 weeks postpartum for both mothers and infants. For each scheduled study visit there is an accepted visit window. If the accepted window has passed and no visit occurred, the visit will be considered missed, forms submitted with any available information, and the subsequent visit scheduled. In addition to scheduled visits, any unscheduled follow-ups or hospital visits during

For peer review only - http://bmjopen.bmj.com/site/about/guidelines.xhtml

the study period will be documented.

Harmonized data collection

Trained study staff will use harmonized measurement procedures and data collection tools developed for the purpose of this study [33]. Each tool corresponds to a scheduled or ad hoc study visit conducted either at a study facility or at home and contains information on approximately 4,000 core variables. For maternal participants, the scope of data collected during antenatal care includes sociodemographic characteristics, health behaviors, environmental exposures, medical and obstetric history, anthropometry, vital and clinical status, current physical and mental health state, medications and treatments, vaccination status, laboratory tests, ultrasounds, and diagnostic assessments (**Table 3**).

Table 3. Type of data collected at scheduled antenatal study visits

Type of Data Collected	Scheduled Study Timepoint[1] [✓ required; O optional]				
	**<20 weeks**	**20 weeks**	**28 weeks**	**32 weeks**	**36 weeks**
Prescreening	✓				
Ultrasound exam	✓	O	O	✓	O
Study enrollment	✓				
Sociodemographics	✓				
Clinical status	✓	✓	✓	✓	✓
Anthropometry	✓	✓	✓	✓	✓
Vital signs	✓	✓	✓	✓	✓
Lab tests	✓	✓	✓	✓	✓
Depression screen[2]		✓		✓	

For peer review only - http://bmjopen.bmj.com/site/about/guidelines.xhtml

For peer review only

Quality of life[3]	✓	
Quality of care		O

[1]Additional ad hoc forms are administered in the event of hospitalization, severe adverse events, protocol deviations, and death (verbal autopsy)
[2]Depressive symptoms measured by an adapted version of the Edinburgh Postnatal Depression Scale
[3]Measured by the 12-Item Short Form Health Survey (SF-12) and World Health Organization Disability Assessment Schedule (WHODAS 2.0)

As participants may deliver outside of the study facility, data capturing clinical status and events during labor and delivery will be collected via a combination of observation, retrospective self-report or report by birth attendant, and/or abstraction from medical records. Most maternal assessments will then repeat during scheduled study visits in the postpartum period (**Table 4**). For infant participants, data collected during postnatal care visits includes anthropometry, signs and symptoms of infection, clinical and vital signs, vaccination status, medications and treatments, and cognitive development assessments (**Table 4**).

Table 4. Type of data collected at scheduled delivery and postnatal study visits

Type of Data Collected	Scheduled Study Timepoint[1] [✓ required; O optional; ⧫ only if in study facility]						
	Delivery	Postpartum					
		<1 week	1 week	4 weeks	6 weeks	26 weeks	52 weeks
**Maternal**							
Clinical status	✓	✓	✓	✓	✓	✓	✓
Anthropometry	⧫				✓	✓	✓
Vital signs	✓	✓	✓	✓	✓	✓	✓
Lab tests	O	✓	✓	✓	✓	✓	✓
Depression[2]					✓		
Quality of life[3]				✓			

For peer review only - http://bmjopen.bmj.com/site/about/guidelines.xhtml

Delivery outcome	✓						
Quality of care	O						O
Study close-out					**		✓
**Infant**							
Birth outcome	✓						
Apgar score	✓						
Anthropometry	⧫	✓	✓	✓	✓	✓	✓
Jaundice screen[4]	⧫	✓	✓				
Vital signs[5]		✓	✓	✓	✓	✓	✓
Clinical status		✓	✓	✓	✓	✓	✓
Lab tests					✓	✓	✓
Vaccination		✓	✓	✓	✓	✓	✓
Cognitive development[6]							✓
Stimulation[7]							O
Study close-out							✓

✓Required

OOptional

⧫Only collected if delivery occurred in study facility

***Close-out occurs at 6 weeks in the case of spontaneous or induced abortion*

[1]Additional ad hoc forms are administered in the event of hospitalization, severe adverse events, protocol deviations, and death (verbal autopsy)

[2]Depressive symptoms measured by an adapted version of the Edinburgh Postnatal Depression Scale

[3]Measured by the 12-Item Short Form Health Survey (SF-12) and World Health Organization Disability Assessment Schedule (WHODAS 2.0)

[4]Measured by Mennen Medical Bili-Care transcutaneous bilirubin device and Bili-ruler visual ichometer

[5]Heart rate and oxygen saturation

[6]Measured by the Global Scale for Early Development Short Form (version 1.0)

[7]Measured by the Family Care Indicators assessment

For peer review only

For peer review only - http://bmjopen.bmj.com/site/about/guidelines.xhtml

Laboratory and diagnostic tests

Maternal and infant participants will undergo study-specific laboratory and diagnostic testing at scheduled visits (**Table 5**). Wherever possible and with consideration to participant burden, gold standard methods will be used. Hemoglobin levels will be assessed at every antenatal and postnatal visit and routine urinalysis done by dipstick at all antenatal visits. In addition to these assessments, each study contact point has specific requirements. Results will be provided in real time to participants for all ultrasound exams, rapid diagnostic tests, and as available for blood tests. Where specified, specimens may be stored for later batch testing, in which case results will still be communicated with participants and subsequent referrals made as appropriate. Soluble fms-like tyrosine kinase-1 and placental growth factor biomarkers will not be used in clinical decisionmaking, as these are under investigation for clinical utility.

During the enrollment visit, pregnancy viability and fetal biometry will be determined via ultrasound for gestational age dating and to verify study eligibility. Blood tests will be done for hemoglobin A1c levels to assess for diabetes. Additional blood specimens will be collected to test for Rh factor, micronutrient deficiencies, inflammatory markers, liver and renal function, soluble fms-like tyrosine kinase-1, and placental growth factor. Rapid diagnostic tests will be conducted for HIV, malaria, syphilis, and hepatitis B and C infection. If the syphilis rapid test is positive, it will be followed up with a nontreponemal test to check syphilis titers. If clinically indicated by the World Health Organization Four-Symptom Screen, a tuberculosis test will also be done [34].

At the 28-week antenatal visit, an oral glucose tolerance test will be done to screen for gestational diabetes among participants without previously diagnosed or overt diabetes (i.e., normal hemoglobin A1c levels at enrollment). Blood specimens will be stored for soluble fms-like tyrosine kinase-1 and placental growth factor biomarker testing. A repeat ultrasound to measure fetal biometry is recommended at 28 weeks and required at 32 weeks. At the 32-week antenatal visit, blood tests will also be repeated for micronutrient deficiencies, inflammatory markers, and thyroid

For peer review only - http://bmjopen.bmj.com/site/about/guidelines.xhtml

282 function. If the syphilis rapid test is negative at enrollment, it will be repeated. If the rapid test is positive, it will be followed up with a nontreponemal test to check syphilis titers. At the 36-week antenatal visit, the rapid diagnostic tests for HIV, malaria, syphilis, and tuberculosis infection are repeated as clinically indicated or locally recommended.

286 At the six-week postnatal visit, an additional maternal blood specimen will be collected to test for micronutrient deficiencies and inflammation. For participants who tested positive for gestational diabetes during antenatal care, an oral glucose tolerance test will be done at the six-week visit along with a hemoglobin A1c test at the final 52-week visit.

290 Further testing among a subsample or time-limited testing of specific infections or biomarkers will be incorporated at the discretion of the PRISMA consortium to better understand novel findings or previously undetected health concerns. Beginning in November 2023, sites asynchronously rolled out vaginal swab testing for chlamydia and gonorrhea at the enrollment and 32-week antenatal care visits, which will continue for one year of data collection. Among a subsample of 1,000 participants per site, testing for differential diagnosis of febrile illnesses—including hepatitis E, Zika, chikungunya, dengue, and leptospirosis infection—will be administered at enrollment, 32 weeks gestation, and six weeks postpartum. Depending on the identified burden of disease, investigators may choose to continue testing.

299 ***Table 5. Maternal laboratory and diagnostic tests during antenatal and postnatal care***

Assessment	Scheduled Study Visit					
	Antenatal care (weeks)				Postnatal care (weeks)	
	<20	28	32	36	6	52
Hemoglobin levels[1]	✓	✓	✓	✓	✓	✓
Urinalysis[1]	✓	✓	✓	✓		
Rh factor	✓					

For peer review only - http://bmjopen.bmj.com/site/about/guidelines.xhtml

Hemoglobin A1c	✓					*
Oral glucose tolerance		✓[2]			*	
Micronutrient deficiencies[3]	✓		✓		✓	
Inflammation biomarkers[4]	✓		✓		✓	
Preeclampsia biomarkers[5]	✓	✓	✓			
Liver function	✓					
Renal function	✓					
Thyroid function			✓			
Syphilis infection	✓		✓	*		
Syphilis titers	*		*			
Chlamydia infection	+		+			
Gonorrhea infection	+		+			
HIV infection	✓			*		
Malaria infection	✓			*		
Tuberculosis infection	*			*		
Hepatitis B infection	✓					
Hepatitis C infection	✓					
Hepatitis E infection	+		+		+	
Zika infection	+		+		+	
Chikungunya infection	+		+		+	
Dengue infection	+		+		+	
Leptospirosis	+		+		+	

✓Required

**As clinically indicated*

+Testing completed for a subset of participants at each site, following which investigators will decide whether or not to continue with these tests based on the observed burden of disease

[1]Select visits not shown due to limited labs required: antenatal visit at 20 weeks (hemoglobin and urinalysis only) and postnatal visits at <1, 1, 4, and 26 weeks (hemoglobin only).

[2]Only among women without previously diagnosed or overt diabetes (i.e. normal HbA1c at enrollment)

For peer review only

[3]*Vitamin B12 total cobalamin, folate, vitamin A retinol binding protein, iodine thyroglobulin, iron serum transferrin receptor, and iron ferritin*
[4]*C-reactive protein and alpha 1-acid glycoprotein*
[5]*Soluble fms-like tyrosine kinase-1 and placental growth factor*

Infants similarly will undergo a limited set of laboratory and diagnostic tests at scheduled time points (**Table 6**). At birth, transcutaneous bilirubin assessment is done within 72 hours (24 hours preferred). Transcutaneous bilirubin assessments will be repeated at the three-to-five-day visit (<1 week) and the one-week visit. Hemoglobin levels will be assessed by HemoCue at the six-week, six-month, and one-year visits. Tests for malaria infection will be performed at six months and one year. Infants will be tested for HIV and hepatitis B tests only in the case of maternal infection.

Table 6. Infant laboratory and diagnostic tests at birth and during postnatal care

Assessment	Scheduled Study Visit						
	Birth	Postnatal (weeks)					
		<1	1	4	6	26	52
Bilirubin levels[1]	✓	✓	✓				
Hemoglobin levels					✓	✓	✓
Malaria infection						✓	✓
HIV infection					*	*	*
Hepatitis B virus							*

✓*Required*
**In the case of maternal infection*
[1]*Bilirubin measured via transcutaneous bilirubinometer and Bili-ruler visual ichometer*

Quality control and assurance

All procedures will adhere to Quality Assurance and Quality Control Standard Operating Procedures that were collaboratively developed for this study. Central training was conducted for designated study staff members from each site to ensure harmonized procedures for ultrasound examinations, verbal autopsies, and assessment for integrated management of childhood

For peer review only
For peer review only - http://bmjopen.bmj.com/site/about/guidelines.xhtml

illnesses. Personnel taking anthropometric measurements received study-specific training and will participate in ongoing standardization exercises at regular intervals. Calibration checks of circumference tapes, scales, stadiometers, and infantometers for anthropometric assessments will be performed monthly. An external monitoring group has been contracted to ensure that the rights and safety of study subjects are protected; to ensure the quality and integrity of study data; and to ensure the study is conducted in compliance with the approved protocol and applicable regulatory requirements.

A proportion of ultrasound images will undergo external quality assessment, and all fetal biometry measures will undergo plausibility checks. Sonographers at all sites will be certified in study-specific procedures and undergo annual refresher training to ensure compliance. We will use Tricefy™ for secure cloud medical imaging storage and sharing of anonymized data. Anonymized images will be randomly peer-reviewed and expert-reviewed; summary quality reports will then be generated monthly.

Biospecimen collection and laboratory testing will be conducted according to Standard Operating Procedures. Laboratories implementing micronutrient measurements on site will be enrolled in the Centers for Disease Control Performance Verification Program for Serum Micronutrients. For full blood count measurement, all participating laboratories will be enrolled in the United Kingdom National External Quality Assessment Scheme for Hematology. For full blood count and all other analytes, laboratories must also be enrolled in the College of American Pathologists' external quality assurance program.

Data quality monitoring

To ensure the success of the study, a baseline phase was carried out in 2021 to pilot the performance of study tools and procedures. Performance was evaluated using both qualitative

For peer review only

and quantitative methods. At the end of the pilot period, a harmonization process to standardize tools and data systems across study sites was implemented.

Obtaining high-quality data is central to the study; thus, data quality monitoring is a key component of standard operating procedures. Each country team will be responsible for the initial data cleaning, including running of routine range and consistency checks as well as periodic reviews of distributions and identification of outliers. Any inconsistencies within their database will be resolved in consultation with the clinical and field data collection team. A cleaned, limited dataset from all sites will be stored in a secure central data repository cloud server, shared at bi-weekly intervals. Data analysts on the coordination team will run additional data quality checks and produce both data queries and monitoring reports using pooled data on at least a bi-monthly basis. Data queries will identify issues related to the following: range checks, skip patterns, consistency logic, date and time concordance, submitted records to the database, uniqueness of a record (i.e., de-duplicating), outliers, digit preference, and missingness. Monitoring reports will include details such as rate of enrollment, protocol compliance, completeness of key clinical and lab assessments, and the distribution of key health outcomes. Data management teams will share codes for data checks via GitHub repositories.

Data management systems and security

Data collection instruments were developed by each country's team. All systems are encrypted to ensure no subject information is readily accessible. Signed consent forms and study data collection forms will be stored in a secure location with access limited to authorized research staff. A limited study dataset, which will not contain any protected health information or personally identifiable information beyond complete birth and death dates, will be maintained in a secure central repository. This dataset may be shared with current and future research partners or affiliates for analysis purposes through a data sharing agreement. Any partners given permission to access the data will be required to adhere to the Gates Foundation Global Access policies,

For peer review only - http://bmjopen.bmj.com/site/about/guidelines.xhtml

which require that any knowledge and information gained or any products developed must be made available and accessible at an affordable price to the people most in need living in LMICs.

Patient and public involvement

Each implementing institution has its own community advisory board. During initial protocol development, investigators evaluated the additional burden of study participation on patients; for this reason, invasive clinical assessments are kept to a minimum and grounded in evidence-based care guidelines. Patients' experiences were centered during the translation and validation process for questionnaires to measure postpartum depression and maternal morbidity symptoms. When selecting the primary and secondary outcomes, the prevalence and impact of each outcome within study site contexts was strongly considered. Upon completion of the study, we intend to share the main findings with participants and community members via appropriate local dissemination methods. Finally, we will ensure that participants' contributions to this research are acknowledged in any subsequent reports, presentations, and publications.

ETHICS AND DISSEMINATION

Ethical and safety considerations

This protocol, the informed consent documents, and any subsequent modifications will be reviewed and approved by the relevant institutional review board (IRB) and ethics review committee (ERC) responsible for oversight of the study at each site. Approvals were received from the following IRBs and ERCs in each country: Ghana (Ghana Health Service ERC: 019/09/20; Kintampo Health Research Centre Institutional Ethics Committee: KHRCIEC/2020-17); India (Office of Research, Christian Medical College, Vellore, India: IRB14553; Ethics Review Committee, Society for Applied Studies, New Delhi: SAS/ERC/ReMAPPStudy/2022; Kenya (KEMRI Scientific and Ethics Review Unit: KEMRI/SERU/4166; Liverpool School of Tropical Medicine Ethics Committee: 23-020 Jaramogi Oginga Odinga Teaching and Referral Hospital (JOOTRH) Institutional Scientific Ethics Review Committee ISERC: ISERC/JOOTRH/549/2024);

For peer review only - http://bmjopen.bmj.com/site/about/guidelines.xhtml

Pakistan (The Aga Khan University ERC: No. 2023-9100-26333; Pakistan National Institutes of Health—Health Research Institute, National Bioethics Committee: 4-87/NBCR-1023/23/973); Zambia (University of North Carolina Chapel Hill Office of Human Research Ethics: Study No. 14-2113; University of Zambia Biomedical Research Ethics Committee: 016-04-14); and the United States (The George Washington University IRB: NCR224396; Columbia University IRB: IRB-AAAU7504; Harvard University IRB: IRB23-1093; The University of Alabama at Birmingham IRB: IRB-300013081). All pregnant women will be provided with information about the study and the option to opt-out of data collection. For women who are not of legal age for consent, assent together with parental or other legal consent must be obtained per local standards. Study participants will be referred to higher-level or specialty care according to site-specific standard operating procedures.

For peer review only

Dissemination plan

The data generated in the course of the study will be reviewed on an ongoing basis, according to priorities set by the investigators. Quarterly and annual reports will be shared with key stakeholders, as well as the funder (Gates Foundation). Interim findings with potential implications for informing public health policy or practice will be shared with major local and international stakeholders, including the World Health Organization, United States Centers for Disease Control, United States Agency for International Development, and Ministries of Health in each participating country. Local meetings will be organized to share the findings of this study with community members and study participants at each site. Abstracts from both country-specific and pooled global data analyses will be developed for dissemination through both local and international scientific conferences and publication in peer-reviewed journals.

AUTHORS' CONTRIBUTIONS

This study is the result of an international collaborative effort carried out by a large group of institutions and members of the PRISMA Consortium. Conceptualization: ERS, ZH, MIN, SM, NS,

For peer review only - http://bmjopen.bmj.com/site/about/guidelines.xhtml

ASJ, BJW, VA, WKM, DG, AGC, SJB, VRM, MPK, MBS, WM, KPA, SN, CTA, and LML. Funding acquisition: ERS, CM, ZH, MIN, SM, VA, AGC, SJB, MPK, MBS, WM, KPA, SN, CTA, and LML. Methodology: ERS, CM, QP, JS, CRS, ZH, MIN, FJ, SM, NS, ASJ, BJW, VA, WKM, DG, AGC, SJB, VRM, MPK, MBS, WM, BF, KPA, SN, and CTA. Project administration: SGB, JM, AH, NY, AK, FS, KF, NS, ASJ, JW, ZA, GO, HO, SV, JS, DJ, JA, FM, HM, BF, BV, MS, IAA, VA, EO-L, and SG. Resources: CM, AH, LC, AD, MD, RV, KO, BV, HM, VA, and DAG. Supervision: ERS, CM, QP, JS, CRS, ZH, MIN, FJ, AH, SM, RT, BJW, VA, WKM, DG, KO, AGC, SJB, VRM, MPK, MBS, WM, BF, BV, KPA, SN, and CTA. Writing—original draft: ERS, SGB, and CRS. Writing—review & editing: ERS, CM, QP, JS, SGB, JM, CRS, ZH, MIN, FJ, AH, NY, AK, FS, KF, LC, AD, MD, RV, SM, NS, ASJ, RT, BJW, VA, WKM, JW, DG, ZA, GO, HO, KO, AGC, SJB, VRM, BV, SV, JS, DJ, JA, MPK, MBS, WM, FM, BF, BV, MS, KPA, SN, CTA, IAA, VA, DAG, EO-L, SG, and LML. ERS will act as the guarantor.

FUNDING STATEMENT

This work is supported by the Gates Foundation grant number [INV-047400 to KPA, CTA, and SN; INV-057219 to VA; INV-043092 to SB; INV-005776 to VPT; INV-057220 to ZH; INV-016221 to MPK; K01TW012426 NIH/FIC to MBS; INV-057222 to WM; INV-041999 to ERS; INV-060797 to CM; and INV-057223 to SM].

ACKNOWLEDGEMENTS

The Maternal and Newborn Health Study is a research project of the Pregnancy Risk, Infant Surveillance, and Measurement Alliance (PRISMA) Consortium. PRISMA members are grateful to the extensive network of individuals and organizations who contributed to the design of this study, including but not limited to biostatisticians, laboratory technicians, obstetricians, community health workers, field data collectors, and research partners at the Institute for Health Metrics and Evaluation, Centers for Disease Control and Prevention, and the World Health Organization. The

For peer review only

For peer review only - http://bmjopen.bmj.com/site/about/guidelines.xhtml

authors also acknowledge the efforts of study participants and community members, without whom this would not be possible.

COMPETING INTERESTS STATEMENT

The authors have no competing interests to declare.

COLLABORATOR STATEMENT

The Pregnancy Risk, Infant Surveillance, and Measurement Alliance (PRISMA) Investigators include: Emily R. Smith, Christopher Mores, Qing Pan, Jennifer Seager, Sasha G. Baumann, Jaime Marquis, Christopher R. Sudfeld, Zahra Hoodbhoy, Muhammad Imran Nisar, Fyezah Jehan, Aneeta Hotwani, Nida Yazdani, Amna Khan, Farzana Shaheen, Kinza Farooqui, Leena Chatterjee, Arjun Dang, Manavi Dang, R Venketeshwar, Sarmila Mazumder, Neeraj Sharma, Arun Singh Jadaun, Rupa Talukdar, Blair J. Wylie, Victor Akelo, Winnie K. Mwebia, Joyce Were, Dickson Gethi, Zacchaeus Abaja, Gregory Ouma, Harun Owuor, Kephas Otieno, Anne George Cherian, Santosh Joseph Benjamin, Venkata Raghava Mohan, Balakrishnan Vijayalekshmi, Sunitha Varghese, Jasmine Sugirtha, Daniel Jebakumar, James A, Margaret P. Kasaro, M. Bridget Spelke, Wilbroad Mutale, Felistas Mbewe, Humphrey Mwape, Bethany Freeman, Bellington Vwalika, Mutale Sampa, Kwaku Poku Asante, Sam Newton, Charlotte Tawiah Agyemang, Irene Apewe Adjei, Veronica Agyemang, Dennis Adu-Gyasi, Eliezer Odei-Lartey, Stephaney Gyaase, and Laura M. Lamberti.

For peer review only

For peer review only - http://bmjopen.bmj.com/site/about/guidelines.xhtml

REFERENCES

1. Alkema L, Chou D, Hogan D, Zhang S, Moller AB, Gemmill A, et al. Global, regional, and national levels and trends in maternal mortality between 1990 and 2015, with scenario-based projections to 2030: a systematic analysis by the UN Maternal Mortality Estimation Inter-Agency Group. Lancet. 2016 Jan 30;387(10017):462–74.

2. Who. Trends in maternal mortality 2000 to 2017: estimates by WHO, UNICEF, UNFPA, World Bank Group and the United Nations Population Division. World Health Organization Geneva, Switzerland; 2019.

3. WHO, UNICEF, UNFPA, World Bank Group and UNDESA/Population Division. Trends in maternal mortality 2000 to 2020 [Internet]. World Health Organization; 2023. Report No.: ISBN 978-92-4-006875-9. Available from: https://iris.who.int/bitstream/handle/10665/366225/9789240068759-eng.pdf?sequence=1

4. Say L, Chou D, Gemmill A, Tunçalp Ö, Moller AB, Daniels J, et al. Global causes of maternal death: a WHO systematic analysis. Lancet Glob Health. 2014 Jun;2(6):e323–33.

5. Musarandega R, Nyakura M, Machekano R, Pattinson R, Munjanja SP. Causes of maternal mortality in Sub-Saharan Africa: A systematic review of studies published from 2015 to 2020. J Glob Health. 2021 Oct 9;11:04048.

6. Hussein J. Non-communicable diseases during pregnancy in low and middle income countries. Obstet Med. 2017 Mar;10(1):26–9.

7. Firoz T, Pineles B, Navrange N, Grimshaw A, Oladapo O, Chou D. Non-communicable diseases and maternal health: a scoping review. BMC Pregnancy Childbirth. 2022 Oct 22;22(1):787.

8. O'Neil S, Taylor S, Sivasankaran A. Data equity to advance health and health equity in low- and middle-income countries: A scoping review. Digit Health. 2021 Jan 22;7:20552076211061922.

9. Souza JP, Day LT, Rezende-Gomes AC, Zhang J, Mori R, Baguiya A, et al. A global analysis of the determinants of maternal health and transitions in maternal mortality. Lancet Glob Health. 2024 Feb 1;12(2):e306–16.

10. Geller SE, Koch AR, Garland CE, MacDonald EJ, Storey F, Lawton B. A global view of severe maternal morbidity: moving beyond maternal mortality. Reprod Health. 2018 Jun 22;15(Suppl 1):98.

11. Lori S. Ashford, Elizabeth Ransom, Nancy Yinger. Hidden Suffering: Disabilities From Pregnancy and Childbirth in Less Developed Countries [Internet]. PRB. 2002 [cited 2024 Dec 3]. Available from: https://www.prb.org/resources/hidden-suffering-disabilities-from-pregnancy-and-childbirth-in-less-developed-countries/

12. Sukmanee J, Liabsuetrakul T. Risk of future cardiovascular diseases in different years postpartum after hypertensive disorders of pregnancy: A systematic review and meta-analysis. Medicine (Baltimore). 2022 Jul 29;101(30):e29646.

For peer review only - http://bmjopen.bmj.com/site/about/guidelines.xhtml

For peer review only

13. Azulay CE, Pariente G, Shoham-Vardi I, Kessous R, Sergienko R, Sheiner E. Maternal anemia during pregnancy and subsequent risk for cardiovascular disease. J Matern Fetal Neonatal Med. 2015 Oct 13;28(15):1762–5.

14. Li J, Song C, Li C, Liu P, Sun Z, Yang X. Increased risk of cardiovascular disease in women with prior gestational diabetes: A systematic review and meta-analysis. Diabetes Res Clin Pract. 2018 Jun 1;140:324–38.

15. Latt SM, Alderdice F, Elkington M, Awng Shar M, Kurinczuk JJ, Rowe R. Primary postpartum haemorrhage and longer-term physical, psychological, and psychosocial health outcomes for women and their partners in high income countries: A mixed-methods systematic review. PLoS One. 2023 Jun 14;18(6):e0274041.

16. Lawn JE, Ohuma EO, Bradley E, Idueta LS, Hazel E, Okwaraji YB, et al. Small babies, big risks: global estimates of prevalence and mortality for vulnerable newborns to accelerate change and improve counting. Lancet. 2023 May 20;401(10389):1707–19.

17. Vogel JP, Lee ACC, Souza JP. Maternal morbidity and preterm birth in 22 low- and middle-income countries: a secondary analysis of the WHO Global Survey dataset. BMC Pregnancy Childbirth. 2014 Jan 31;14(1):56.

18. Korzeniewski SJ, Sutton E, Escudero C, Roberts JM. The Global Pregnancy Collaboration (CoLab) symposium on short- and long-term outcomes in offspring whose mothers had preeclampsia: A scoping review of clinical evidence. Front Med (Lausanne). 2022 Aug 30;9:984291.

19. Turbeville HR, Sasser JM. Preeclampsia beyond pregnancy: long-term consequences for mother and child. Am J Physiol Renal Physiol. 2020 Jun 1;318(6):F1315–26.

20. Fraser A, Lawlor DA. Long-term health outcomes in offspring born to women with diabetes in pregnancy. Curr Diab Rep. 2014 Mar 25;14(5):489.

21. Feig DS, Artani A, Asaf A, Li P, Booth GL, Shah BR. Long-term neurobehavioral and metabolic outcomes in offspring of mothers with diabetes during pregnancy: A large, population-based cohort study in Ontario, Canada. Diabetes Care. 2024 Sep 1;47(9):1568–75.

22. Hao X, Lu J, Yan S, Tao F, Huang K. Maternal pre-pregnancy body mass index, gestational weight gain and children's cognitive development: A Birth Cohort study. Nutrients [Internet]. 2022 Nov 1;14. Available from: https://www.mdpi.com/2072-6643/14/21/4613

23. Kwok J, Hall HA, Murray AL, Lombardo MV, Auyeung B. Maternal infections during pregnancy and child cognitive outcomes. BMC Pregnancy Childbirth. 2022 Nov 17;22(1):848.

24. Celi LA, Cellini J, Charpignon ML, Dee EC, Dernoncourt F, Eber R, et al. Sources of bias in artificial intelligence that perpetuate healthcare disparities-A global review. PLOS Digit Health. 2022 Mar;1(3):e0000022.

25. Hasan BS, Rasheed MA, Wahid A, Kumar RK, Zuhlke L. Generating evidence from contextual clinical research in Low- to Middle Income Countries: A roadmap based on theory of change. Front Pediatr. 2021 Dec 9;9:764239.

For peer review only - http://bmjopen.bmj.com/site/about/guidelines.xhtml

26. Bhakuni H, Abimbola S. Epistemic injustice in academic global health. Lancet Glob Health. 2021 Oct;9(10):e1465–70.

27. Acharya KP, Pathak S. Applied research in low-income countries: Why and how? Front Res Metr Anal. 2019 Nov 14;4:3.

28. Ruhighira JJ, Mashili FL, Tungu AM, Mamuya S. Spirometry profiles among pregnant and non-pregnant African women: a cross-sectional study. BMC Womens Health. 2022 Dec 2;22(1):483.

29. Goldstein RF, Abell SK, Ranasinha S, Misso ML, Boyle JA, Harrison CL, et al. Gestational weight gain across continents and ethnicity: systematic review and meta-analysis of maternal and infant outcomes in more than one million women. BMC Med. 2018 Aug 31;16(1):153.

30. Bouthoorn SH, Gaillard R, Steegers EAP, Hofman A, Jaddoe VWV, van Lenthe FJ, et al. Ethnic differences in blood pressure and hypertensive complications during pregnancy: the Generation R study. Hypertension. 2012 Jul;60(1):198–205.

31. AMANHI (Alliance for Maternal and Newborn Health Improvement), Baqui A, Ahmed P, Dasgupta SK, Begum N, Rahman M, et al. Development and validation of a simplified algorithm for neonatal gestational age assessment - protocol for the Alliance for Maternal Newborn Health Improvement (AMANHI) prospective cohort study. J Glob Health. 2017 Dec;7(2):021201.

32. Goldenberg RL, Saleem S, Ali S, Moore JL, Lokangako A, Tshefu A, et al. Maternal near miss in low-resource areas. Int J Gynaecol Obstet. 2017 Sep;138(3):347–55.

33. Smith ER, Mores C, Tawiah C, Asante KP, Mazumder S, Cherian AG, et al. PRISMA Maternal & Newborn Health Study [Internet]. Open Science Foundation; 2024 [cited 2024 Nov 15]. Available from: https://osf.io/qckyt

34. World Health Organization. WHO operational handbook on tuberculosis [Internet]. Genève, Switzerland: World Health Organization; 2021. Available from: https://iris.who.int/bitstream/handle/10665/340256/9789240022614-eng.pdf

For peer review only - http://bmjopen.bmj.com/site/about/guidelines.xhtml

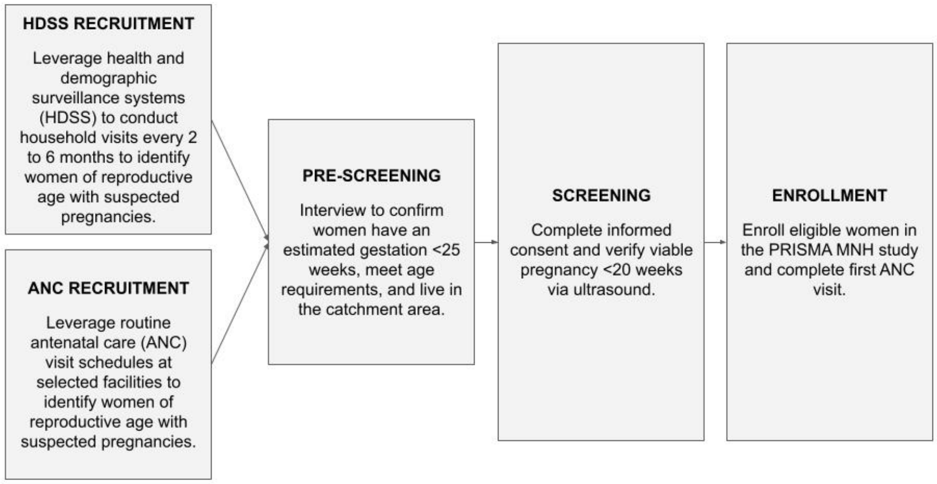

338x190mm (72 x 72 DPI)

SUPPLEMENT 1. OUTCOME DEFINITIONS

Primary Outcomes

1. Maternal mortality: Death from any cause related to or aggravated by pregnancy or its management (excluding accidental or incidental causes) during pregnancy and childbirth or within 42 days of termination of pregnancy, irrespective of the duration and site of the pregnancy.

2. Composite severe maternal outcomes: Composite outcome comprising maternal deaths, late maternal deaths, and maternal near-misses, each defined as the following:

    a. *Maternal death*: Death from any cause related to or aggravated by pregnancy or its management (excluding accidental or incidental causes) during pregnancy and childbirth or within 42 days of termination of pregnancy, irrespective of the duration and site of the pregnancy.

    b. *Late maternal death*: Death from any cause related to or aggravated by pregnancy or its management (excluding accidental or incidental causes), irrespective of the duration and site of the pregnancy, from 42 days postpartum up to one year.

    c. *Maternal near-miss*: Woman who nearly died but survived a life-threatening condition that occurred during pregnancy, childbirth or within 42 days of termination of pregnancy. Consideration as a near-miss case is irrespective of gestational age and includes women who have abortions or stillbirths. Criteria as follows:

        i. Organ dysfunction
        ii. Laparotomy (excluding cesarean section, including hysterectomy)
        iii. Referral to intensive care unit
        iv. Blood transfusion (any units) combined with severe anemia, severe postpartum hemorrhage, antepartum hemorrhage, or placental abruption
        v. Severe postpartum hemorrhage (≥1000 mL estimated blood loss or procedure including hysterectomy and blood transfusion)
        vi. Preeclampsia with severe features/eclampsia
        vii. Uterine rupture
        viii. Sepsis

3. Maternal anemia: Low hemoglobin levels throughout pregnancy and labor and delivery, classified as mild (10-10.9 g/dL), moderate (7-9.9 g/dL), or severe (<7 g/dL). Low hemoglobin levels in the postpartum period, classified as mild (11-11.9 g/dL), moderate (8-10.9 g/dL), or severe (<8 g/dL).

4. Stillbirth: Delivery of a fetus showing no signs of life, as indicated by absence of breathing, heartbeat, pulsation of the umbilical cord, or definite movements of voluntary muscles. Defined as death prior to delivery of a fetus at ≥20 weeks of gestation (or

For peer review only

>350g weight, if gestational age is unavailable). Additionally, we will analyze time-specific definitions:

a. Early stillbirth: 20-27 weeks,
b. Late stillbirth: 28-36 weeks
c. Term stillbirth: ≥37 weeks
d. WHO-defined stillbirth: ≥28 weeks.

5. Neonatal mortality: Death of a liveborn baby during the first 28 days of life from any cause.

6. Low birthweight: Birth weight <2500 g as assessed at delivery or within 72 hours for home births. Very low birth weight if <1500 g.

7. Preterm birth: Delivery prior to 37 completed weeks of gestation of a liveborn infant. Further classified as: extremely preterm (<28 weeks), very preterm (28-32 weeks), and moderate to late preterm (32-37 weeks). Gestational age at birth will be determined by the best obstetric estimate: last menstrual period, ultrasound, and the American College of Obstetricians and Gynecologists algorithm. Preterm delivery is separately defined as delivery prior to 37 completed weeks of gestation (live or stillbirth).

8. Small for gestational age: Birth weight of an infant of lower than the sex-specific 3rd and 10th percentile for each week of gestational age per INTERGROWTH standards. Combined gestational age information and birthweight will be used to further categorize into preterm-small-for-gestational-age (SGA), preterm-appropriate-for-gestational-age (AGA), term-SGA, and term-AGA.

Secondary Outcomes

9. Late maternal mortality: Death from any cause related to or aggravated by pregnancy or its management (excluding accidental or incidental causes), irrespective of the duration and site of the pregnancy, from 42 days postpartum up to one year.

10. Preeclampsia: Hypertensive disorder of pregnancy defined for those without preexisting hypertension as gestational hypertension AND gestational proteinuria OR clinical diagnosis of preeclampsia OR development of severe features (even in the absence of proteinuria). Defined for those with chronic hypertension as incident gestational proteinuria OR clinical diagnosis of preeclampsia OR development of severe features. Further defined as early-onset preeclampsia if <34 weeks gestation and late-onset if ≥34 weeks. Sub definitions as follows:

a. *Chronic (preexisting) hypertension*: ≥140/90 mm Hg before pregnancy or <20th week of gestation or use of antihypertensive medication before pregnancy OR history of hypertension OR reported taking antihypertensive medications.

For peer review only

b. *Gestational hypertension*: A systolic blood pressure ≥140 mm Hg on two occasions at least 1 hour apart or diastolic blood pressure ≥90 mm Hg on two occasions at least 1 hour apart during pregnancy.

c. *Gestational proteinuria*: 24-hour urine collection >300 mg protein OR single voided urine protein/creatinine ratio ≥0.3 OR dipstick reading of 1+ or 2+ (use only if other quantitative methods are not available).

d. *Severe features* (indicated by presence of one or more of the following):

i. *Severe gestational hypertension*: during pregnancy defined as systolic blood pressure >160 mmHg or a diastolic blood pressure >110 mmHg on two occasions at least 1 minute apart after 20 weeks gestation; or at the time of delivery defined as one systolic blood pressure >160 mmHg or a diastolic blood pressure >110 mmHg among participants without chronic hypertension >160/110 (even in absence of proteinuria)

ii. *Hepatic abnormality*: impaired liver function not accounted for by another diagnosis and characterized by serum transaminase concentration ≥2 times the upper limit of the normal range

iii. *Severe persistent right upper quadrant or epigastric pain*: unresponsive to medication and not accounted for by an alternative diagnosis

iv. *New onset neurological symptoms*: such as headache (unresponsive to medication and not accounted for by alternative diagnoses) or visual symptoms

v. *Thrombocytopenia*: <100,000 platelets/microL

vi. *Renal insufficiency*: defined as serum creatinine >1.1 mg/dL or a doubling of the serum creatinine concentration in the absence of other renal disease

vii. *Pulmonary edema and/or eclampsia*: clinical report of new-onset tonic-clonic, focal, or multifocal seizures in the absence of other causative conditions such as epilepsy, cerebral arterial ischemia and infarction, intracranial hemorrhage, or drug use

viii. *Evidence of end organ dysfunction*: by laboratory criteria

ix. *Clinical diagnosis of HELLP syndrome*: based upon the presence of ALL of the laboratory abnormalities (hemolysis, elevated liver enzymes, and low platelet count)

1. Hemolysis (established by at least two of the following): peripheral smear with schistocytes and burr cells, serum bilirubin ≥1.2 mg/dL (20.52 umol/L), low serum haptoglobin (≤25 mg/dL) or lactate dehydrogenase (LDH) ≥2 times the upper level of normal (based on

For peer review only

laboratory-specific reference ranges), severe anemia (unrelated to blood loss), and

2. Elevated blood concentrations of liver transaminases (aspartate aminotransferase (AST) or alanine aminotransferase (ALT) to ≥2 times normal concentration (based on laboratory-specific reference ranges)

3. Low platelets or thrombocytopenia <100 x 109/L)

11. Gestational hypertension: A systolic blood pressure ≥140 mm Hg on two occasions at least 1 hour apart or diastolic blood pressure≥90 mm Hg on two occasions at least 1 hour apart or severe gestational hypertension in pregnancy (not at time of delivery) defined as systolic blood pressure > 160 mmHg or a diastolic blood pressure >110 mmHg on two occasions at least 1 minute apart after 20 weeks gestation or severe gestational hypertension at the time of delivery defined as one systolic blood pressure > 160 mmHg or a diastolic blood pressure >110 mmHg among participants without chronic hypertension. Chronic hypertension defined by a clinical diagnosis of chronic hypertension (with or without need for medication) or systolic blood pressure >140 mm Hg on two occasions at least1 hour apart or diastolic blood pressure >90 mm Hg on two occasions at least 1 hour apart before 20 weeks' gestation OR medication used OR prior clinical diagnosis. Medications for hypertension include magnesium sulfate, hydralazine, methyldopa (aldomet), atenolol, nifedipine, betamethasone, labetalol, and dexamethasone.

12. Postpartum hypertension: Systolic blood pressure ≥140 mm Hg or diastolic blood pressure ≥90 mm Hg on two occasions at least 1 hour apart in the postpartum period.

13. Preterm birth indication: Delivery prior to 37 completed weeks of gestation of a birth (live or stillbirth), classified into spontaneous preterm or provider-initiated preterm. Spontaneous preterm is defined as delivery <37 weeks that occurs either secondary to preterm labor or preterm premature rupture of membranes. Provider-initiated preterm defined as a medical or obstetric complication or other reason that the health care provider initiates delivery at <37 completed weeks gestation.

14. Preterm premature rupture of membranes: Rupture of membranes before the onset of labor occurring before <37 weeks of gestation or clinical diagnosis of premature rupture of membranes.

15. Gestational diabetes: Diabetes first diagnosed during pregnancy and characterized by insulin resistance and high blood sugar. Clinical diagnosis or diagnosis by 75g oral glucose tolerance test (OGTT) around 28 weeks based on the International Association of the Diabetes and Pregnancy Study Groups thresholds: fasting > 5.1 mmol/L or 1-hr OGTT >10.0 mmol/L or 2-hr OGTT >8.5 mmol/L.

16. Maternal infection and sepsis: Defined as organ dysfunction (per WHO near-miss definition) resulting from infection during pregnancy, delivery, and over 42 days

For peer review only - http://bmjopen.bmj.com/site/about/guidelines.xhtml

postpartum. Categories of infection are defined based on severity, as defined by the Global Maternal Sepsis Study:

a. *Infection-related severe maternal outcomes*: women presenting with WHO near-miss criteria to define organ system dysfunction and maternal death

b. *Infections with complications*: women with an invasive procedure to treat the source of infection (vacuum aspiration, dilatation and curettage, wound debridement, drainage, laparotomy and lavage, other surgery), or admission to ICU, or transfer to another facility

c. *Less severe infections*: all other infections.

17. Perinatal depression: Defined as a clinical mood disorder during pregnancy and up to 42 days postpartum. Symptoms screened using the Edinburgh Postnatal Depression Scale. Threshold for defining depression will be country specific based on local validation studies: Kenya ≥13; Ghana: ≥11; Pakistan ≥14; India (Vellore): ≥8; India (Hodal) ≥10; Zambia ≥10.

18. Fetal death: A product of human conception, irrespective of the duration of the pregnancy, which, after expulsion or extraction, does not breath or show any other evidence of life such as beating of the heart, pulsation of the umbilical cord, or definite movement of voluntary muscles, whether or not the umbilical cord has been cut or the placenta is attached.

19. Infant mortality: Death of a neonate or an infant from delivery up to one year of life.

20. Cause of neonatal mortality: Causes of neonatal death based on verbal autopsy.

21. Timing of neonatal mortality: Death of a liveborn during the first 28 days of life. Neonatal deaths are classified by timing: <24 hours (i.e. first 24 hours of life), early neonatal mortality (i.e. first 7 days), or late neonatal mortality (i.e. between 7 and 28 days).

22. Cause of stillbirth: Causes of stillbirth based on verbal autopsy.

23. Timing of stillbirth: Delivery of a fetus showing no signs of life, as indicated by absence of breathing, heartbeat, pulsation of the umbilical cord, or definite movements of voluntary muscles. Death occurring prior to delivery of a fetus at ≥20 weeks of gestation (or >350 g weight, if gestational age is unavailable). . Antepartum stillbirth, defined as heart rate not detected ≥12 hrs prior to delivery and/or signs of maceration. Intrapartum stillbirth, defined as heart rate detected <12 hrs prior to delivery and/or no signs of maceration.

24. Hyperbilirubinemia: Defined as the presence of excess bilirubin during the first week of life (assessed at birth, 3 days, and 7 days of life). Outcome based on: continuous bilirubin by age (hours), proportion >75th percentile, and proportion >95th percentile.

25. Neonatal sepsis: Defined as inflammatory response and organ dysfunction following presence of a severe infection from delivery to 28 days as suspected (by a clinician) or

For peer review only

proven (with culture). Further classified as early onset (i.e. delivery to 72 hours) or late onset (72 hours to 28 days).

26. Possible severe bacterial infection: Presence of any of the following clinical signs or symptoms as defined by the WHO IMCI criteria, which are consistent with possible severe bacterial infection, at the time of delivery to 59 days: not able to feed at all or not feeding well, convulsions, severe chest indrawing, high body temperature (≥38°C), low body temperature (less than 35.5°C), no movement/only when stimulated, and/or fast breathing (≥60 BPM) in infants less than 7 days old.

27. Postnatal weight trajectory: Continuous weight change from birth through one month of age. Trajectory is determined by percent body weight loss by 72 hours, time to regain birth weight, and continuous body weight.

28. Infant growth: Proportion of infants more than 2 standard deviations below the WHO Children Health Reference Growth Standard for: weight for age (underweight), length for age (stunting), length for weight (wasting), and/or head circumference for age (microcephaly).

Tertiary Outcomes

29. Placental disorders: Placental abruption is defined as the complete or partial separation of the placenta from the inner wall of the uterus before delivery. Signs include vaginal bleeding (painful or painless), pain during abdominal palpitations, and/or fetal distress. Diagnosis made via ultrasound. Placenta previa is defined at the partial or complete covering of the cervix by the placenta. Signs include vaginal bleeding without pain in the second or third trimester. Diagnosis made via ultrasound.

30. Uterine rupture: Uterine rupture is defined as the spontaneous tearing of the uterus prior to delivery that may result in the fetus being expelled into the peritoneal cavity. Symptoms and signs of uterine rupture include fetal bradycardia, variable decelerations, evidence of hypovolemia, loss of fetal station (detected during cervical examination), severe or constant abdominal pain, and cessation of uterine contractions, and/or recession of the presenting part.

31. Prolonged labor: Defined as labor lasting ≥24 hours.

32. Unplanned surgery: The occurrence of medically indicated or emergent surgical procedure during labor and delivery. Hysterectomy is defined as the removal of the uterus (i.e. partial) or of the uterus and the cervix (i.e. total hysterectomy). Cesarean section is defined as a surgical procedure used to deliver a baby through incisions in the abdomen and uterus.

33. Hemorrhage:

    a. *Antepartum hemorrhage*: Bleeding from or into the genital tract, occurring from ≥24 weeks gestation and prior to delivery.

For peer review only

b. *Postpartum hemorrhage*: Defined as estimated blood loss of 500 mL or more within 24 hours after birth or clinician diagnosis.

c. *Severe postpartum hemorrhage*: perceived abnormal bleeding (1000 ml or more) or any bleeding with hypotension or blood transfusion.

34. Perinatal birth asphyxia: Clinician-reported failure to breathe spontaneously in the first minute after delivery.

For peer review only

BMJ Open

Protocol for the Pregnancy Risk, Infant Surveillance, and Measurement Alliance (PRISMA) Maternal and Newborn Health Study: A multi-site, prospective, open cohort study of pregnancy and postpartum health outcomes in South Asia and sub-Saharan Africa

Journal:	*BMJ Open*
Manuscript ID	bmjopen-2025-104512.R2
Article Type:	Protocol
Date Submitted by the Author:	31-Oct-2025
Complete List of Authors:	Maternal and Newborn Health Study Investigators, PRISMA; The George Washington University, Department of Global Health<br>Smith, Emily; The George Washington University, Department of Global Health
**Primary Subject Heading**:	Obstetrics and gynaecology
Secondary Subject Heading:	Paediatrics, Global health, Epidemiology, Infectious diseases, Nutrition and metabolism
Keywords:	Pregnancy, Postpartum Period, Maternal medicine < OBSTETRICS, Protocols & guidelines < HEALTH SERVICES ADMINISTRATION & MANAGEMENT, Fetal medicine < OBSTETRICS

SCHOLARONE™
Manuscripts

TITLE

Protocol for the Pregnancy Risk, Infant Surveillance, and Measurement Alliance (PRISMA) Maternal and Newborn Health Study: A multi-site, prospective, open cohort study of pregnancy and postpartum health outcomes in South Asia and sub-Saharan Africa

CORRESPONDING AUTHOR

Emily R. Smith
The George Washington University Milken Institute School of Public Health
950 New Hampshire Ave NW #2, Washington, DC 20037
emilysmith@gwu.edu

AUTHORS

Pregnancy Risk, Infant Surveillance, and Measurement Alliance (PRISMA) Investigators

For peer review only

ABSTRACT

Introduction: Maternal and child mortality has markedly decreased worldwide over the past few decades. Despite this success, the decline remains unequal across countries and is overall insufficient to meet the Sustainable Development Goals. South Asia and sub-Saharan Africa bear most of the burden of maternal and child morbidity and mortality. Major gaps persist in our understanding of the causes, timing, diagnostic thresholds, and risk factors for adverse outcomes in these regions. Addressing these gaps requires new ways to prevent and treat disease, from novel diagnostics to precision public health strategies, all of which rely on high-quality clinical data from diverse populations. The Pregnancy Risk, Infant Surveillance, and Measurement Alliance (PRISMA) Maternal and Newborn Health Study aims to estimate population-level prevalence of morbidities and mortality and to assess biological, clinical, and sociodemographic risk among mother-infant pairs in India, Pakistan, Kenya, Ghana, and Zambia.

Methods and analysis: This study is a prospective, open cohort study with a planned recruitment of about 6,000 women annually across six research sites in five countries. Participants are pregnant women enrolled less than 20 weeks gestation, as determined by ultrasound, identified through active house-to-house and facility-based surveillance. Robust clinical data will be collected at 12 scheduled study visits during antenatal care, labor and delivery, and through one year postpartum. A total of 34 outcomes will be captured. The primary analysis will estimate the burden of adverse outcomes and examine associated risk factors to inform future intervention strategies. Data will also be used to develop normative values for pregnant and postpartum women, as well as predictive models to assess pregnancy risk.

Ethics and dissemination: PRISMA received institutional and national ethical approvals. Findings will be published in peer-reviewed open-access journals and disseminated at national and international forums to inform clinical guidelines and public health practice.

For peer review only - http://bmjopen.bmj.com/site/about/guidelines.xhtml

Strengths and limitations:

- Harmonized, multidimensional data collection on social, biological, clinical, and environmental factors related to health and disease.
- Large, community-based sample of an estimated 18,000 pregnant women and 15,300 live births over the first three years of the study.
- Use of combined cutting-edge machine learning algorithms and predictive modeling approaches to better understand and predict risks.
- Morbidity and mortality estimates may not be fully generalizable to other settings.

For peer review only

For peer review only - http://bmjopen.bmj.com/site/about/guidelines.xhtml

INTRODUCTION

Globally, the maternal mortality rate has declined by more than 43% since 1990 [1]. Despite this success, progress remains unequal between regions, with low- and middle-income countries (LMICs) bearing excess burden [2]. Southern Asia and sub-Saharan Africa account for 86% of all maternal deaths and have some of the world's highest maternal mortality rates at 134 (80% uncertainty interval (UI) 118 to 155) and 545 (UI 477 to 654) per 100,000 live births, for each region respectively [3]. The World Health Organization estimates that between 2003 and 2009 the top causes of death in both regions were hemorrhage, hypertensive disorders, and sepsis [4]. These three causes were responsible for over half of all maternal deaths. Indirect causes—such as HIV, malaria, tuberculosis, iron deficiency anemia, or diabetes mellitus—accounted for another 27.5% of maternal deaths [4]. However, due to challenges in event reporting and linking hospital records to national health surveillance systems, comprehensive data on the causes of maternal deaths and near-misses are lacking [5–8].

Pregnancy-related and pre-existing conditions are a growing concern, not only given their potential to be life-threatening, but also due to the lasting effects they have on maternal and infant health and quality of life [9,10]. It is well evidenced that maternal morbidities put women at risk of future complications and impact an estimated 4% to 8% of all deliveries [11]. However, the true burden of maternal morbidities remains unclear, as these conditions frequently go undiagnosed or undocumented, most existing research focuses on a single sequela, and few large clinical cohorts have been conducted in LMICs. For example, a review of 59 studies (only four of which were conducted in Southeast Asia and sub-Saharan Africa) found that hypertensive disorders of pregnancy more than doubled the risk of ischemic heart disease and heart failure later in life [12]. Similarly, gestational diabetes and maternal anemia have been identified as risk factors for long-term cardiovascular diseases [13,14]. Another systematic review of 24 studies in high-income countries found that postpartum hemorrhage was linked to persistent physical, psychological, and

For peer review only - http://bmjopen.bmj.com/site/about/guidelines.xhtml

psychosocial problems, including mastitis, post-traumatic stress disorder, depression, and delayed mother-infant bonding [15]. The narrow clinical and geographic focus of available research limits our understanding of the full range of risks women face during and after pregnancy, and how this varies across health settings. Multidimensional research that assesses biological, clinical, demographic, and social determinants of health is likely to improve risk prediction, thereby preventing maternal morbidities and reducing their enduring repercussions.

Infant health and development also suffer the consequences of maternal morbidities [10,16]. Data from a World Health Organization Global Survey of 172,461 singleton deliveries in 22 LMICs found that preeclampsia and pre-gestational diabetes significantly increased odds of provider-initiated and spontaneous preterm birth [17]. Preeclampsia and gestational diabetes have been connected to heightened risk of life-long adverse outcomes among offspring, including increased susceptibility to hypertension, kidney disease, nutritional deficiencies, cardiometabolic events, obesity, attention deficit hyperactivity disorder, and autism spectrum disorder [18–21]. Maternal infections and poor nutrition during pregnancy have been linked to suboptimal cognitive development for children [22,23]. To provide newborns with the best start to a healthy life, improvements in antenatal care are essential to appropriately manage pre-existing conditions and screen for risk factors. Ultimately, addressing maternal morbidities not only benefits women's health but further supports the long-term well-being of their children.

While early identification of morbidities and risk for adverse outcomes in the antenatal and postnatal periods is essential, correct diagnoses and risk prediction are hindered by the lack of empirical data underlying clinical guidance and risk stratification algorithms. Often these are predominantly derived from data collected among non-pregnant adults in limited geographies, most commonly the United States and China, due to concentrated investments in healthcare research and extensive electronic health systems [24–27]. Data disparities limit the generalizability and predictive accuracy when applied to peripartum women [25]. The rise of

For peer review only

For peer review only - http://bmjopen.bmj.com/site/about/guidelines.xhtml

machine learning has further highlighted the issue, as generated predictive algorithms typically rely on datasets from high-income settings, restricting their applicability to LMICs [24]. High quality, representative data is required to inform clinical guidance and risk prediction tools for maternal morbidities; better data is needed to inform normative ranges for perinatal depressive symptoms, blood pressure, biomarkers for hypertensive disorders of pregnancy, and hemoglobin levels [28–30]. Addressing these evidence gaps by generating multidimensional global datasets for use in clinical benchmarking is critical to enabling accurate, equitable care for all women.

Study rationale and objectives

Robust data on pregnancy risks—including medical history, clinical symptoms and diagnoses, behavioral and lifestyle factors, social and economic characteristics, environmental exposures, and nutritional status—are essential to developing strategies to effectively manage pregnancy risks and improve outcomes in resource-constrained environments [31,32]. Population-based, multidimensional, longitudinal data of mother-infant pairs is severely lacking, particularly from LMICs. This hinders our ability to estimate the prevalence of morbidities and mortality, and to attribute their various risk factors among pregnant women and infants during the antenatal and postnatal periods [31]. We further lack the detailed, individual patient data needed to develop advanced diagnostic tools and risk prediction algorithms, as well as adequate baseline data needed to effectively monitor for adverse events in the context of introducing new devices, drugs, and vaccines. Existing datasets have significant limitations in study designs or the completeness and accuracy of data collected. Often, data are self-reported, cross-sectional, and most datasets are unable to be pooled in a harmonized way. These limitations make it difficult to achieve the robust datasets needed for analysis and for prioritizing investments in research, development, and policy or guideline changes.

We designed the Pregnancy Risk, Infant Surveillance, and Measurement Alliance (PRISMA) Maternal and Newborn Health Study in response to these critical gaps. At a high level, the study

For peer review only - http://bmjopen.bmj.com/site/about/guidelines.xhtml

aims to evaluate pregnancy risk factors and their associations with adverse outcomes, including stillbirth, infant mortality and morbidity, and maternal mortality and morbidity. The resulting harmonized dataset will be used to estimate population-level disease and outcome burden in two South Asian and three sub-Saharan African countries, which will be evaluated to identify any overlooked health concerns in each study area. Our data will inform subsequent research to understand priority issues and to test innovative strategies that optimize pregnancy outcomes, including defining more representative and gestational-age-specific reference values for select biomarkers. The primary objectives are as follows: (1) To provide population-based prevalence estimates of key maternal and infant health outcomes, which may inform the development of future diagnostics and interventions; (2) To improve the global understanding of key risk factors for morbidity and mortality among pregnant women, their fetuses, and mother-infant pairs for up to one year postpartum; and (3) To collect data to enable the application of novel analytical techniques to create risk prediction tools.

METHODS AND ANALYSIS

Study design

The PRISMA Maternal and Newborn Health Study is an international, multisite, prospective, open-cohort study. Leveraging population-based and facility-based pregnancy surveillance systems, we will identify, screen, and enroll pregnant participants prior to 20 weeks gestation as confirmed by ultrasound. Enrollment began in September 2022 in Pakistan, November 2022 in Kenya, December 2022 in Ghana and Kenya, June 2023 in South India, and December 2023 in North India. Detailed clinical data will be collected to characterize the health status of women and their fetuses and infants, including ultrasound imaging, laboratory testing, physical examination, validated health surveys, medical history by maternal recall, and facility record abstraction. Data collection will continue during the antepartum, intrapartum, and postpartum periods up to one year following delivery. Participants will be assessed at scheduled visits at <20, 20, 28, 32, and 36

For peer review only - http://bmjopen.bmj.com/site/about/guidelines.xhtml

weeks gestation, at labor and delivery, at three days postpartum, and one, four, six, 26, and 52 weeks postpartum. Liveborn infants will be similarly assessed at birth, day three of life, and one, four, six, 26, and 52 weeks old. Any additional visits or hospitalizations, for example due to complications, will also be documented. In the event of a miscarriage or stillbirth, participants will be followed up through six weeks and one year, respectively. Verbal autopsies will be conducted in the case of maternal, fetal, neonatal, or infant death and analyzed by InterVA computer software to ensure a harmonized approach to cause of death determination. The study protocol will be implemented uniformly across sites to ensure that all laboratory and diagnostics tests, primary health outcomes, and related risk factors are measured correctly and consistently.

Study outcomes

We aim to capture data on 34 adverse health outcomes (**Table 1**), which are defined in **Supplement 1**. Outcomes were selected based on high-priority research gaps within study contexts and determined via an interactive protocol development process with input from study investigators and other subject matter experts. Feasibility was also considered when selecting the measurement method. Clinical definitions, measurements, and constructed variables for each health outcome are standardized across study sites.

Table 1. Study outcomes by population and level

Outcome Level	Population	
	**Gravida/Puerperium**	**Fetus/Infant**
Primary	(1) Maternal mortality<br>(2) Composite severe maternal outcomes<br>(3) Maternal anemia	(4) Stillbirth<br>(5) Neonatal mortality<br>(6) Low birthweight<br>(7) Preterm birth<br>(8) Small-for-gestational-age
Secondary	(9) Late maternal mortality<br>(10) Preeclampsia<br>(11) Gestational hypertension<br>(12) Postpartum hypertension<br>(13) Medical indication for preterm delivery	(18) Fetal death<br>(19) Infant mortality<br>(20) Cause of neonatal mortality<br>(21) Timing of neonatal mortality<br>(22) Cause of stillbirth

For peer review only

	(14) Preterm premature rupture of membranes<br>(15) Gestational diabetes<br>(16) Maternal infection and sepsis<br>(17) Perinatal depression	(23) Timing of stillbirth<br>(24) Hyperbilirubinemia<br>(25) Neonatal sepsis<br>(26) Possible severe bacterial infection<br>(27) Postnatal weight trajectory<br>(28) Infant growth
Tertiary	(29) Placental disorders<br>(30) Uterine rupture<br>(31) Prolonged labor<br>(32) Unplanned surgery<br>(33) Antepartum and postpartum hemorrhage	(34) Perinatal birth asphyxia

Study settings

Data collection will occur in six study areas: Kisumu and Siaya, Kenya; Kintampo, Ghana; Lusaka, Zambia; Vellore, India; Hodal, India; and Rehri Goth and Ibrahim Hyderi, Karachi, Pakistan. Protocol implementation is jointly carried out by clinical teams at participating health facilities and study staff from partnering research institutions (i.e. the Kenya Medical Research Institute, Kintampo Health Research Centre, University of North Carolina Global Projects Zambia, Christian Medical College Vellore, Society for Applied Studies, and Aga Khan University). Clinical assessments and subsequent findings conducted by study staff will be integrated with clinical care. Researchers at the George Washington University are responsible for protocol harmonization and central data coordination. Consortium institutions and investigators were appointed by the Gates Foundation based on existing research infrastructure and expertise. Each country team identified and mapped geographic areas in which to conduct the study. Estimates of the study area population size are described in **Table 2**.

Table 2. Estimates of study area population size, pregnancies, and live births

Study Area	No. Households	No. Women of Reproductive Age	Total Population Size	Anticipated No. Pregnancies (Live Births)[1]
Kintampo, Ghana	34,640	45,767	177,200	3,000 (2,250)
Vellore, India	54, 435	47,443	234,624	2,700 (2,300)
Hodal, India	36,321	35,499	196,678	2,000 (1,700)

For peer review only - http://bmjopen.bmj.com/site/about/guidelines.xhtml

Kisumu, Kenya	44,932	47,826	171,698	3,000 (2,550)
Karachi, Pakistan	23,170	34,682	227,549	6,000 (5,100)
Lusaka, Zambia	31,165	34,942	145,263	2,250 (1,914)
**All sites**	**224,663**	**246,159**	**1,153,012**	**18,550 (15,814)**

[1]*Anticipated number of pregnancies based off expected annual enrollment over three years; expected 85% of pregnancies to result in a live birth*

Eligibility criteria

Women who meet the following criteria may be eligible for enrollment: lives within the study catchment area with no plans to permanently relocate during pregnancy or within one year postpartum; meets minimum age requirement for consenting in country of residence (Ghana: 15 years of age; Kenya: 18 years of age or emancipated minors; Pakistan: 15 years of age or emancipated minors; Zambia: 15 years of age; India: 18 years of age); and viable intrauterine pregnancy less than 20 weeks gestation determined by ultrasound. Women who have more than one pregnancy during the study period are eligible for re-enrollment. Multiple gestations (i.e., two, three, or more fetuses) will also be eligible.

Pregnancy surveillance

Household-based and facility-based surveillance systems will be used to identify women of reproductive age with suspected pregnancies. Routine health and demographic surveillance systems, either existing or established for the purpose of this study, will be leveraged to visit homes in the study catchment area at least once every six months. Women identified at these visits will be invited to provide basic socio-demographic information as a part of the population-based surveillance and screened for study eligibility. Women that receive antenatal care at participating health facilities will also be identified and screened. Combined, these women make up the sampling frame for the study cohort.

For peer review only

For peer review only - http://bmjopen.bmj.com/site/about/guidelines.xhtml

Participant identification and enrollment

A multiphase process of pre-screening, consenting, and screening will be used to enroll participants (**Figure 1**). Identified women of reproductive age will be prescreened for potential eligibility by community fieldworkers based on the following criteria: reported symptoms or clinical signs of pregnancy; estimated gestational age per last menstrual period <25 weeks; lives within the study catchment area; and meets minimum age requirement in each study site. Any woman potentially eligible after pre-screening will be invited to provide informed consent or assent in her preferred language. Certified sonographers will then conduct an ultrasound to verify that the woman has an intrauterine pregnancy and is prior to 20 weeks gestation. Women who are beyond the gestational age threshold or with a non-viable pregnancy (e.g., ectopic) are not eligible to participate and will be referred.

Figure 1. Overview of surveillance and identification of potentially eligible women

Study follow-up procedures

Follow-up is planned to occur through one year postpartum for participants with liveborn and stillborn infants, and through six weeks for participants with spontaneous or induced abortions. Data will be collected at 12 study visits scheduled according to gestational age. This includes five antenatal study visits at <20 weeks (enrollment), 20 weeks, 28 weeks, 32 weeks, and 36 weeks gestation. Data will also be collected for labor and delivery within 24 hours for facility births and 72 hours for home births. Deliveries will be observed by study staff, when possible, otherwise data will be abstracted from medical records and by maternal or provider recall. After delivery, there will be six postnatal visits at three days, one week, four weeks, six weeks, 26 weeks, and 52 weeks postpartum for both mothers and infants. For each scheduled study visit there is an accepted visit window. If the accepted window has passed and no visit occurred, the visit will be considered missed, forms submitted with any available information, and the subsequent visit scheduled. In addition to scheduled visits, any unscheduled follow-ups or hospital visits during

For peer review only - http://bmjopen.bmj.com/site/about/guidelines.xhtml

the study period will be documented.

Harmonized data collection

Trained study staff will use harmonized measurement procedures and data collection tools developed for the purpose of this study [33]. Each tool corresponds to a scheduled or ad hoc study visit conducted either at a study facility or at home and contains information on approximately 4,000 core variables. For maternal participants, the scope of data collected during antenatal care includes sociodemographic characteristics, health behaviors, environmental exposures, medical and obstetric history, anthropometry, vital and clinical status, current physical and mental health state, medications and treatments, vaccination status, laboratory tests, ultrasounds, and diagnostic assessments (**Table 3**).

Table 3. Type of data collected at scheduled antenatal study visits

Type of Data Collected	Scheduled Study Timepoint[1] [✓ required; O optional]				
	<20 weeks	20 weeks	28 weeks	32 weeks	36 weeks
Prescreening	✓				
Ultrasound exam	✓	O	O	✓	O
Study enrollment	✓				
Sociodemographics	✓				
Clinical status	✓	✓	✓	✓	✓
Anthropometry	✓	✓	✓	✓	✓
Vital signs	✓	✓	✓	✓	✓
Lab tests	✓	✓	✓	✓	✓
Depression screen[2]		✓		✓	

For peer review only

Quality of life[3]	✓	
Quality of care		O

[1]Additional ad hoc forms are administered in the event of hospitalization, severe adverse events, protocol deviations, and death (verbal autopsy)
[2]Depressive symptoms measured by an adapted version of the Edinburgh Postnatal Depression Scale
[3]Measured by the 12-Item Short Form Health Survey (SF-12) and World Health Organization Disability Assessment Schedule (WHODAS 2.0)

As participants may deliver outside of the study facility, data capturing clinical status and events during labor and delivery will be collected via a combination of observation, retrospective self-report or report by birth attendant, and/or abstraction from medical records. Most maternal assessments will then repeat during scheduled study visits in the postpartum period (**Table 4**). For infant participants, data collected during postnatal care visits includes anthropometry, signs and symptoms of infection, clinical and vital signs, vaccination status, medications and treatments, and cognitive development assessments (**Table 4**).

Table 4. Type of data collected at scheduled delivery and postnatal study visits

**Type of Data Collected**	**Scheduled Study Timepoint[1]** [✓ required; O optional; ⧫ only if in study facility]						
	**Delivery**	**Postpartum**					
		**<1 week**	**1 week**	**4 weeks**	**6 weeks**	**26 weeks**	**52 weeks**
**Maternal**							
Clinical status	✓	✓	✓	✓	✓	✓	✓
Anthropometry	⧫				✓	✓	✓
Vital signs	✓	✓	✓	✓	✓	✓	✓
Lab tests	O	✓	✓	✓	✓	✓	✓
Depression[2]					✓		
Quality of life[3]				✓			

For peer review only - http://bmjopen.bmj.com/site/about/guidelines.xhtml

Delivery outcome	✓						
Quality of care	O						O
Study close-out					**		✓
**Infant**							
Birth outcome	✓						
Apgar score	✓						
Anthropometry	⧫	✓	✓	✓	✓	✓	✓
Jaundice screen[4]	⧫	✓	✓				
Vital signs[5]		✓	✓	✓	✓	✓	✓
Clinical status		✓	✓	✓	✓	✓	✓
Lab tests					✓	✓	✓
Vaccination		✓	✓	✓	✓	✓	✓
Cognitive development[6]							✓
Stimulation[7]							O
Study close-out							✓

✓Required

ᴼOptional

⧫Only collected if delivery occurred in study facility

***Close-out occurs at 6 weeks in the case of spontaneous or induced abortion*

[1]Additional ad hoc forms are administered in the event of hospitalization, severe adverse events, protocol deviations, and death (verbal autopsy)

[2]Depressive symptoms measured by an adapted version of the Edinburgh Postnatal Depression Scale

[3]Measured by the 12-Item Short Form Health Survey (SF-12) and World Health Organization Disability Assessment Schedule (WHODAS 2.0)

[4]Measured by Mennen Medical Bili-Care transcutaneous bilirubin device and Bili-ruler visual ichometer

[5]Heart rate and oxygen saturation

[6]Measured by the Global Scale for Early Development Short Form (version 1.0)

[7]Measured by the Family Care Indicators assessment

For peer review only

Laboratory and diagnostic tests

Maternal and infant participants will undergo study-specific laboratory and diagnostic testing at scheduled visits (**Table 5**). Wherever possible and with consideration to participant burden, gold standard methods will be used. Hemoglobin levels will be assessed at every antenatal and postnatal visit and routine urinalysis done by dipstick at all antenatal visits. In addition to these assessments, each study contact point has specific requirements. Results will be provided in real time to participants for all ultrasound exams, rapid diagnostic tests, and as available for blood tests. Where specified, specimens may be stored for later batch testing, in which case results will still be communicated with participants and subsequent referrals made as appropriate. Soluble fms-like tyrosine kinase-1 and placental growth factor biomarkers will not be used in clinical decisionmaking, as these are under investigation for clinical utility.

During the enrollment visit, pregnancy viability and fetal biometry will be determined via ultrasound for gestational age dating and to verify study eligibility. Blood tests will be done for hemoglobin A1c levels to assess for diabetes. Additional blood specimens will be collected to test for Rh factor, micronutrient deficiencies, inflammatory markers, liver and renal function, soluble fms-like tyrosine kinase-1, and placental growth factor. Rapid diagnostic tests will be conducted for HIV, malaria, syphilis, and hepatitis B and C infection. If the syphilis rapid test is positive, it will be followed up with a nontreponemal test to check syphilis titers. If clinically indicated by the World Health Organization Four-Symptom Screen, a tuberculosis test will also be done [34].

At the 28-week antenatal visit, an oral glucose tolerance test will be done to screen for gestational diabetes among participants without previously diagnosed or overt diabetes (i.e., normal hemoglobin A1c levels at enrollment). Blood specimens will be stored for soluble fms-like tyrosine kinase-1 and placental growth factor biomarker testing. A repeat ultrasound to measure fetal biometry is recommended at 28 weeks and required at 32 weeks. At the 32-week antenatal visit, blood tests will also be repeated for micronutrient deficiencies, inflammatory markers, and thyroid

For peer review only

For peer review only - http://bmjopen.bmj.com/site/about/guidelines.xhtml

282 function. If the syphilis rapid test is negative at enrollment, it will be repeated. If the rapid test is
283 positive, it will be followed up with a nontreponemal test to check syphilis titers. At the 36-week
284 antenatal visit, the rapid diagnostic tests for HIV, malaria, syphilis, and tuberculosis infection are
285 repeated as clinically indicated or locally recommended.

286 At the six-week postnatal visit, an additional maternal blood specimen will be collected to test for
287 micronutrient deficiencies and inflammation. For participants who tested positive for gestational
288 diabetes during antenatal care, an oral glucose tolerance test will be done at the six-week visit
289 along with a hemoglobin A1c test at the final 52-week visit.

290 Further testing among a subsample or time-limited testing of specific infections or biomarkers will
291 be incorporated at the discretion of the PRISMA consortium to better understand novel findings
292 or previously undetected health concerns. Beginning in November 2023, sites asynchronously
293 rolled out vaginal swab testing for chlamydia and gonorrhea at the enrollment and 32-week
294 antenatal care visits, which will continue for one year of data collection. Among a subsample of
295 1,000 participants per site, testing for differential diagnosis of febrile illnesses—including hepatitis
296 E, Zika, chikungunya, dengue, and leptospirosis infection—will be administered at enrollment, 32
297 weeks gestation, and six weeks postpartum. Depending on the identified burden of disease,
298 investigators may choose to continue testing.

299 ***Table 5. Maternal laboratory and diagnostic tests during antenatal and postnatal care***

Assessment	Scheduled Study Visit					
	Antenatal care (weeks)				Postnatal care (weeks)	
	<20	28	32	36	6	52
Hemoglobin levels[1]	✓	✓	✓	✓	✓	✓
Urinalysis[1]	✓	✓	✓	✓		
Rh factor	✓					

For peer review only - http://bmjopen.bmj.com/site/about/guidelines.xhtml

Hemoglobin A1c	✓					*
Oral glucose tolerance		✓[2]			*	
Micronutrient deficiencies[3]	✓		✓		✓	
Inflammation biomarkers[4]	✓		✓		✓	
Preeclampsia biomarkers[5]	✓	✓	✓			
Liver function	✓					
Renal function	✓					
Thyroid function			✓			
Syphilis infection	✓		✓	*		
Syphilis titers	*		*			
Chlamydia infection	+		+			
Gonorrhea infection	+		+			
HIV infection	✓			*		
Malaria infection	✓			*		
Tuberculosis infection	*			*		
Hepatitis B infection	✓					
Hepatitis C infection	✓					
Hepatitis E infection	+		+		+	
Zika infection	+		+		+	
Chikungunya infection	+		+		+	
Dengue infection	+		+		+	
Leptospirosis	+		+		+	

✓Required

**As clinically indicated*

+Testing completed for a subset of participants at each site, following which investigators will decide whether or not to continue with these tests based on the observed burden of disease

[1]Select visits not shown due to limited labs required: antenatal visit at 20 weeks (hemoglobin and urinalysis only) and postnatal visits at <1, 1, 4, and 26 weeks (hemoglobin only).

[2]Only among women without previously diagnosed or overt diabetes (i.e. normal HbA1c at enrollment)

For peer review only

[3]Vitamin B12 total cobalamin, folate, vitamin A retinol binding protein, iodine thyroglobulin, iron serum transferrin receptor, and iron ferritin
[4]C-reactive protein and alpha 1-acid glycoprotein
[5]Soluble fms-like tyrosine kinase-1 and placental growth factor

Infants similarly will undergo a limited set of laboratory and diagnostic tests at scheduled time points (**Table 6**). At birth, transcutaneous bilirubin assessment is done within 72 hours (24 hours preferred). Transcutaneous bilirubin assessments will be repeated at the three-to-five-day visit (<1 week) and the one-week visit. Hemoglobin levels will be assessed by HemoCue at the six-week, six-month, and one-year visits. Tests for malaria infection will be performed at six months and one year. Infants will be tested for HIV and hepatitis B tests only in the case of maternal infection.

Table 6. Infant laboratory and diagnostic tests at birth and during postnatal care

Assessment	Scheduled Study Visit						
	Birth	Postnatal (weeks)					
		<1	1	4	6	26	52
Bilirubin levels[1]	✓	✓	✓				
Hemoglobin levels					✓	✓	✓
Malaria infection						✓	✓
HIV infection					*	*	*
Hepatitis B virus							*

✓Required
**In the case of maternal infection*
[1]Bilirubin measured via transcutaneous bilirubinometer and Bili-ruler visual ichometer

Quality control and assurance

All procedures will adhere to Quality Assurance and Quality Control Standard Operating Procedures that were collaboratively developed for this study. Central training was conducted for designated study staff members from each site to ensure harmonized procedures for ultrasound examinations, verbal autopsies, and assessment for integrated management of childhood

For peer review only

For peer review only - http://bmjopen.bmj.com/site/about/guidelines.xhtml

329 illnesses. Personnel taking anthropometric measurements received study-specific training and
330 will participate in ongoing standardization exercises at regular intervals. Calibration checks of
331 circumference tapes, scales, stadiometers, and infantometers for anthropometric assessments
332 will be performed monthly. An external monitoring group has been contracted to ensure that the
333 rights and safety of study subjects are protected; to ensure the quality and integrity of study data;
334 and to ensure the study is conducted in compliance with the approved protocol and applicable
335 regulatory requirements.

336 A proportion of ultrasound images will undergo external quality assessment, and all fetal biometry
337 measures will undergo plausibility checks. Sonographers at all sites will be certified in study-
338 specific procedures and undergo annual refresher training to ensure compliance. We will use
339 Tricefy™ for secure cloud medical imaging storage and sharing of anonymized data. Anonymized
340 images will be randomly peer-reviewed and expert-reviewed; summary quality reports will then
341 be generated monthly.

342 Biospecimen collection and laboratory testing will be conducted according to Standard Operating
343 Procedures. Laboratories implementing micronutrient measurements on site will be enrolled in
344 the Centers for Disease Control Performance Verification Program for Serum Micronutrients. For
345 full blood count measurement, all participating laboratories will be enrolled in the United Kingdom
346 National External Quality Assessment Scheme for Hematology. For full blood count and all other
347 analytes, laboratories must also be enrolled in the College of American Pathologists' external
348 quality assurance program.

349 **Data quality monitoring**

350 To ensure the success of the study, a baseline phase was carried out in 2021 to pilot the
351 performance of study tools and procedures. Performance was evaluated using both qualitative

For peer review only

and quantitative methods. At the end of the pilot period, a harmonization process to standardize tools and data systems across study sites was implemented.

Obtaining high-quality data is central to the study; thus, data quality monitoring is a key component of standard operating procedures. Each country team will be responsible for the initial data cleaning, including running of routine range and consistency checks as well as periodic reviews of distributions and identification of outliers. Any inconsistencies within their database will be resolved in consultation with the clinical and field data collection team. A cleaned, limited dataset from all sites will be stored in a secure central data repository cloud server, shared at bi-weekly intervals. Data analysts on the coordination team will run additional data quality checks and produce both data queries and monitoring reports using pooled data on at least a bi-monthly basis. Data queries will identify issues related to the following: range checks, skip patterns, consistency logic, date and time concordance, submitted records to the database, uniqueness of a record (i.e., de-duplicating), outliers, digit preference, and missingness. Monitoring reports will include details such as rate of enrollment, protocol compliance, completeness of key clinical and lab assessments, and the distribution of key health outcomes. Data management teams will share codes for data checks via GitHub repositories.

Data management systems and security

Data collection instruments were developed by each country's team. All systems are encrypted to ensure no subject information is readily accessible. Signed consent forms and study data collection forms will be stored in a secure location with access limited to authorized research staff. A limited study dataset, which will not contain any protected health information or personally identifiable information beyond complete birth and death dates, will be maintained in a secure central repository. This dataset may be shared with current and future research partners or affiliates for analysis purposes through a data sharing agreement. Any partners given permission to access the data will be required to adhere to the Gates Foundation Global Access policies,

For peer review only

For peer review only - http://bmjopen.bmj.com/site/about/guidelines.xhtml

which require that any knowledge and information gained or any products developed must be made available and accessible at an affordable price to the people most in need living in LMICs.

Patient and public involvement

Patients and/or the public were involved to varying degrees at different study sites in the design of this research, including as participants on community advisory boards that were consulted. They were not directly involved in study recruitment. When selecting the primary and secondary outcomes, the prevalence and impact of each outcome within study site contexts was strongly considered. Additionally, the field data collection teams and study investigators considered the needs and feedback from community members at each step of the study protocol development process. For example, invasive clinical assessments were kept to a minimum to reduce unnecessary burden on participants. Local women were involved in the validation of multiple study tools, including the depression screening questionnaire, and shared their experiences of the assessments through cognitive debriefing interviews. Upon completion of the study, we intend to share the main findings with participants and community members via appropriate local dissemination methods. Finally, we will ensure that participants' contributions to this research are acknowledged in subsequent publications.

For peer review only

ETHICS AND DISSEMINATION

Ethical and safety considerations

This protocol, the informed consent documents, and any subsequent modifications will be reviewed and approved by the relevant institutional review board (IRB) and ethics review committee (ERC) responsible for oversight of the study at each site. Approvals were received from the following IRBs and ERCs in each country: Ghana (Ghana Health Service ERC: 019/09/20; Kintampo Health Research Centre Institutional Ethics Committee: KHRCIEC/2020-17); India (Office of Research, Christian Medical College, Vellore, India: IRB14553; Ethics Review Committee, Society for Applied Studies, New Delhi: SAS/ERC/ReMAPPStudy/2022; Kenya

For peer review only - http://bmjopen.bmj.com/site/about/guidelines.xhtml

(KEMRI Scientific and Ethics Review Unit: KEMRI/SERU/4166; Liverpool School of Tropical Medicine Ethics Committee: 23-020 Jaramogi Oginga Odinga Teaching and Referral Hospital (JOOTRH) Institutional Scientific Ethics Review Committee ISERC: ISERC/JOOTRH/549/2024); Pakistan (The Aga Khan University ERC: No. 2023-9100-26333; Pakistan National Institutes of Health—Health Research Institute, National Bioethics Committee: 4-87/NBCR-1023/23/973); Zambia (University of North Carolina Chapel Hill Office of Human Research Ethics: Study No. 14-2113; University of Zambia Biomedical Research Ethics Committee: 016-04-14); and the United States (The George Washington University IRB: NCR224396; Columbia University IRB: IRB-AAAU7504; Harvard University IRB: IRB23-1093; The University of Alabama at Birmingham IRB: IRB-300013081). All pregnant women will be provided with information about the study and the option to opt-out of data collection. For women who are not of legal age for consent, assent together with parental or other legal consent must be obtained per local standards. Study participants will be referred to higher-level or specialty care according to site-specific standard operating procedures.

Dissemination plan

The data generated in the course of the study will be reviewed on an ongoing basis, according to priorities set by the investigators. Quarterly and annual reports will be shared with key stakeholders, as well as the funder (Gates Foundation). Interim findings with potential implications for informing public health policy or practice will be shared with major local and international stakeholders, including the World Health Organization, United States Centers for Disease Control, United States Agency for International Development, and Ministries of Health in each participating country. Local meetings will be organized to share the findings of this study with community members and study participants at each site. Abstracts from both country-specific and pooled global data analyses will be developed for dissemination through both local and international scientific conferences and publication in peer-reviewed journals.

For peer review only - http://bmjopen.bmj.com/site/about/guidelines.xhtml

AUTHORS' CONTRIBUTIONS

This study is the result of an international collaborative effort carried out by a large group of institutions and members of the PRISMA Consortium. Conceptualization: ERS, ZH, MIN, SM, NS, ASJ, BJW, VA, WKM, DG, AGC, SJB, VRM, MPK, MBS, WM, KPA, SN, CTA, and LML. Funding acquisition: ERS, CM, ZH, MIN, SM, VA, AGC, SJB, MPK, MBS, WM, KPA, SN, CTA, and LML. Methodology: ERS, CM, QP, JS, CRS, ZH, MIN, FJ, SM, NS, ASJ, BJW, VA, WKM, DG, AGC, SJB, VRM, MPK, MBS, WM, BF, KPA, SN, and CTA. Project administration: SGB, JM, AH, NY, AK, FS, KF, NS, ASJ, JW, ZA, GO, HO, SV, JS, DJ, JA, FM, HM, BF, BV, MS, IAA, VA, EO-L, and SG. Resources: CM, AH, LC, AD, MD, RV, KO, BV, HM, VA, and DAG. Supervision: ERS, CM, QP, JS, CRS, ZH, MIN, FJ, AH, SM, RT, BJW, VA, WKM, DG, KO, AGC, SJB, VRM, MPK, MBS, WM, BF, BV, KPA, SN, and CTA. Writing—original draft: ERS, SGB, and CRS. Writing—review & editing: ERS, CM, QP, JS, SGB, JM, CRS, ZH, MIN, FJ, AH, NY, AK, FS, KF, LC, AD, MD, RV, SM, NS, ASJ, RT, BJW, VA, WKM, JW, DG, ZA, GO, HO, KO, AGC, SJB, VRM, BV, SV, JS, DJ, JA, MPK, MBS, WM, FM, BF, BV, MS, KPA, SN, CTA, IAA, VA, DAG, EO-L, SG, and LML. ERS will act as the guarantor.

FUNDING STATEMENT

This work is supported by the Bill and Melinda Gates Foundation grant numbers [INV-047400 to KPA, CTA, and SN; INV-057219 to VA; INV-043092 to SB; INV-005776 to VPT; INV-057220 to ZH; INV-016221 to MPK; INV-057222 to WM; INV-041999 to ERS; INV-060797 to CM; and INV-057223 to SM] and the National Institutes of Health Fogarty International Center [K01TW012426 NIH/FIC to MBS].

ACKNOWLEDGEMENTS

The Maternal and Newborn Health Study is a research project of the Pregnancy Risk, Infant Surveillance, and Measurement Alliance (PRISMA) Consortium. PRISMA members are grateful to the extensive network of individuals and organizations who contributed to the design of this

For peer review only

study, including but not limited to biostatisticians, laboratory technicians, obstetricians, community health workers, field data collectors, and research partners at the Institute for Health Metrics and Evaluation, Centers for Disease Control and Prevention, and the World Health Organization. The authors also acknowledge the efforts of study participants and community members, without whom this would not be possible.

COMPETING INTERESTS STATEMENT

The authors have no competing interests to declare.

COLLABORATOR STATEMENT

The Pregnancy Risk, Infant Surveillance, and Measurement Alliance (PRISMA) Investigators include: Emily R. Smith, Christopher Mores, Qing Pan, Jennifer Seager, Sasha G. Baumann, Jaime Marquis, Christopher R. Sudfeld, Zahra Hoodbhoy, Muhammad Imran Nisar, Fyezah Jehan, Aneeta Hotwani, Nida Yazdani, Amna Khan, Farzana Shaheen, Kinza Farooqui, Leena Chatterjee, Arjun Dang, Manavi Dang, R Venketeshwar, Sarmila Mazumder, Neeraj Sharma, Arun Singh Jadaun, Rupa Talukdar, Blair J. Wylie, Victor Akelo, Winnie K. Mwebia, Joyce Were, Dickson Gethi, Zacchaeus Abaja, Gregory Ouma, Harun Owuor, Kephas Otieno, Anne George Cherian, Santosh Joseph Benjamin, Venkata Raghava Mohan, Balakrishnan Vijayalekshmi, Sunitha Varghese, Jasmine Sugirtha, Daniel Jebakumar, James A, Margaret P. Kasaro, M. Bridget Spelke, Wilbroad Mutale, Felistas Mbewe, Humphrey Mwape, Bethany Freeman, Bellington Vwalika, Mutale Sampa, Kwaku Poku Asante, Sam Newton, Charlotte Tawiah Agyemang, Irene Apewe Adjei, Veronica Agyemang, Dennis Adu-Gyasi, Eliezer Odei-Lartey, Stephaney Gyaase, and Laura M. Lamberti.

For peer review only

For peer review only - http://bmjopen.bmj.com/site/about/guidelines.xhtml

REFERENCES

1. Alkema L, Chou D, Hogan D, Zhang S, Moller AB, Gemmill A, et al. Global, regional, and national levels and trends in maternal mortality between 1990 and 2015, with scenario-based projections to 2030: a systematic analysis by the UN Maternal Mortality Estimation Inter-Agency Group. Lancet. 2016 Jan 30;387(10017):462–74.

2. Who. Trends in maternal mortality 2000 to 2017: estimates by WHO, UNICEF, UNFPA, World Bank Group and the United Nations Population Division. World Health Organization Geneva, Switzerland; 2019.

3. WHO, UNICEF, UNFPA, World Bank Group and UNDESA/Population Division. Trends in maternal mortality 2000 to 2020 [Internet]. World Health Organization; 2023. Report No.: ISBN 978-92-4-006875-9. Available from: https://iris.who.int/bitstream/handle/10665/366225/9789240068759-eng.pdf?sequence=1

4. Say L, Chou D, Gemmill A, Tunçalp Ö, Moller AB, Daniels J, et al. Global causes of maternal death: a WHO systematic analysis. Lancet Glob Health. 2014 Jun;2(6):e323–33.

5. Musarandega R, Nyakura M, Machekano R, Pattinson R, Munjanja SP. Causes of maternal mortality in Sub-Saharan Africa: A systematic review of studies published from 2015 to 2020. J Glob Health. 2021 Oct 9;11:04048.

6. Hussein J. Non-communicable diseases during pregnancy in low and middle income countries. Obstet Med. 2017 Mar;10(1):26–9.

7. Firoz T, Pineles B, Navrange N, Grimshaw A, Oladapo O, Chou D. Non-communicable diseases and maternal health: a scoping review. BMC Pregnancy Childbirth. 2022 Oct 22;22(1):787.

8. O'Neil S, Taylor S, Sivasankaran A. Data equity to advance health and health equity in low- and middle-income countries: A scoping review. Digit Health. 2021 Jan 22;7:20552076211061922.

9. Souza JP, Day LT, Rezende-Gomes AC, Zhang J, Mori R, Baguiya A, et al. A global analysis of the determinants of maternal health and transitions in maternal mortality. Lancet Glob Health. 2024 Feb 1;12(2):e306–16.

10. Geller SE, Koch AR, Garland CE, MacDonald EJ, Storey F, Lawton B. A global view of severe maternal morbidity: moving beyond maternal mortality. Reprod Health. 2018 Jun 22;15(Suppl 1):98.

11. Lori S. Ashford, Elizabeth Ransom, Nancy Yinger. Hidden Suffering: Disabilities From Pregnancy and Childbirth in Less Developed Countries [Internet]. PRB. 2002 [cited 2024 Dec 3]. Available from: https://www.prb.org/resources/hidden-suffering-disabilities-from-pregnancy-and-childbirth-in-less-developed-countries/

12. Sukmanee J, Liabsuetrakul T. Risk of future cardiovascular diseases in different years postpartum after hypertensive disorders of pregnancy: A systematic review and meta-analysis. Medicine (Baltimore). 2022 Jul 29;101(30):e29646.

For peer review only - http://bmjopen.bmj.com/site/about/guidelines.xhtml

13. Azulay CE, Pariente G, Shoham-Vardi I, Kessous R, Sergienko R, Sheiner E. Maternal anemia during pregnancy and subsequent risk for cardiovascular disease. J Matern Fetal Neonatal Med. 2015 Oct 13;28(15):1762–5.

14. Li J, Song C, Li C, Liu P, Sun Z, Yang X. Increased risk of cardiovascular disease in women with prior gestational diabetes: A systematic review and meta-analysis. Diabetes Res Clin Pract. 2018 Jun 1;140:324–38.

15. Latt SM, Alderdice F, Elkington M, Awng Shar M, Kurinczuk JJ, Rowe R. Primary postpartum haemorrhage and longer-term physical, psychological, and psychosocial health outcomes for women and their partners in high income countries: A mixed-methods systematic review. PLoS One. 2023 Jun 14;18(6):e0274041.

16. Lawn JE, Ohuma EO, Bradley E, Idueta LS, Hazel E, Okwaraji YB, et al. Small babies, big risks: global estimates of prevalence and mortality for vulnerable newborns to accelerate change and improve counting. Lancet. 2023 May 20;401(10389):1707–19.

17. Vogel JP, Lee ACC, Souza JP. Maternal morbidity and preterm birth in 22 low- and middle-income countries: a secondary analysis of the WHO Global Survey dataset. BMC Pregnancy Childbirth. 2014 Jan 31;14(1):56.

18. Korzeniewski SJ, Sutton E, Escudero C, Roberts JM. The Global Pregnancy Collaboration (CoLab) symposium on short- and long-term outcomes in offspring whose mothers had preeclampsia: A scoping review of clinical evidence. Front Med (Lausanne). 2022 Aug 30;9:984291.

19. Turbeville HR, Sasser JM. Preeclampsia beyond pregnancy: long-term consequences for mother and child. Am J Physiol Renal Physiol. 2020 Jun 1;318(6):F1315–26.

20. Fraser A, Lawlor DA. Long-term health outcomes in offspring born to women with diabetes in pregnancy. Curr Diab Rep. 2014 Mar 25;14(5):489.

21. Feig DS, Artani A, Asaf A, Li P, Booth GL, Shah BR. Long-term neurobehavioral and metabolic outcomes in offspring of mothers with diabetes during pregnancy: A large, population-based cohort study in Ontario, Canada. Diabetes Care. 2024 Sep 1;47(9):1568–75.

22. Hao X, Lu J, Yan S, Tao F, Huang K. Maternal pre-pregnancy body mass index, gestational weight gain and children's cognitive development: A Birth Cohort study. Nutrients [Internet]. 2022 Nov 1;14. Available from: https://www.mdpi.com/2072-6643/14/21/4613

23. Kwok J, Hall HA, Murray AL, Lombardo MV, Auyeung B. Maternal infections during pregnancy and child cognitive outcomes. BMC Pregnancy Childbirth. 2022 Nov 17;22(1):848.

24. Celi LA, Cellini J, Charpignon ML, Dee EC, Dernoncourt F, Eber R, et al. Sources of bias in artificial intelligence that perpetuate healthcare disparities-A global review. PLOS Digit Health. 2022 Mar;1(3):e0000022.

25. Hasan BS, Rasheed MA, Wahid A, Kumar RK, Zuhlke L. Generating evidence from contextual clinical research in Low- to Middle Income Countries: A roadmap based on theory of change. Front Pediatr. 2021 Dec 9;9:764239.

For peer review only - http://bmjopen.bmj.com/site/about/guidelines.xhtml

26. Bhakuni H, Abimbola S. Epistemic injustice in academic global health. Lancet Glob Health. 2021 Oct;9(10):e1465–70.

27. Acharya KP, Pathak S. Applied research in low-income countries: Why and how? Front Res Metr Anal. 2019 Nov 14;4:3.

28. Ruhighira JJ, Mashili FL, Tungu AM, Mamuya S. Spirometry profiles among pregnant and non-pregnant African women: a cross-sectional study. BMC Womens Health. 2022 Dec 2;22(1):483.

29. Goldstein RF, Abell SK, Ranasinha S, Misso ML, Boyle JA, Harrison CL, et al. Gestational weight gain across continents and ethnicity: systematic review and meta-analysis of maternal and infant outcomes in more than one million women. BMC Med. 2018 Aug 31;16(1):153.

30. Bouthoorn SH, Gaillard R, Steegers EAP, Hofman A, Jaddoe VWV, van Lenthe FJ, et al. Ethnic differences in blood pressure and hypertensive complications during pregnancy: the Generation R study. Hypertension. 2012 Jul;60(1):198–205.

31. AMANHI (Alliance for Maternal and Newborn Health Improvement), Baqui A, Ahmed P, Dasgupta SK, Begum N, Rahman M, et al. Development and validation of a simplified algorithm for neonatal gestational age assessment - protocol for the Alliance for Maternal Newborn Health Improvement (AMANHI) prospective cohort study. J Glob Health. 2017 Dec;7(2):021201.

32. Goldenberg RL, Saleem S, Ali S, Moore JL, Lokangako A, Tshefu A, et al. Maternal near miss in low-resource areas. Int J Gynaecol Obstet. 2017 Sep;138(3):347–55.

33. Smith ER, Mores C, Tawiah C, Asante KP, Mazumder S, Cherian AG, et al. PRISMA Maternal & Newborn Health Study [Internet]. Open Science Foundation; 2024 [cited 2024 Nov 15]. Available from: https://osf.io/qckyt

34. World Health Organization. WHO operational handbook on tuberculosis [Internet]. Genève, Switzerland: World Health Organization; 2021. Available from: https://iris.who.int/bitstream/handle/10665/340256/9789240022614-eng.pdf

For peer review only

For peer review only - http://bmjopen.bmj.com/site/about/guidelines.xhtml

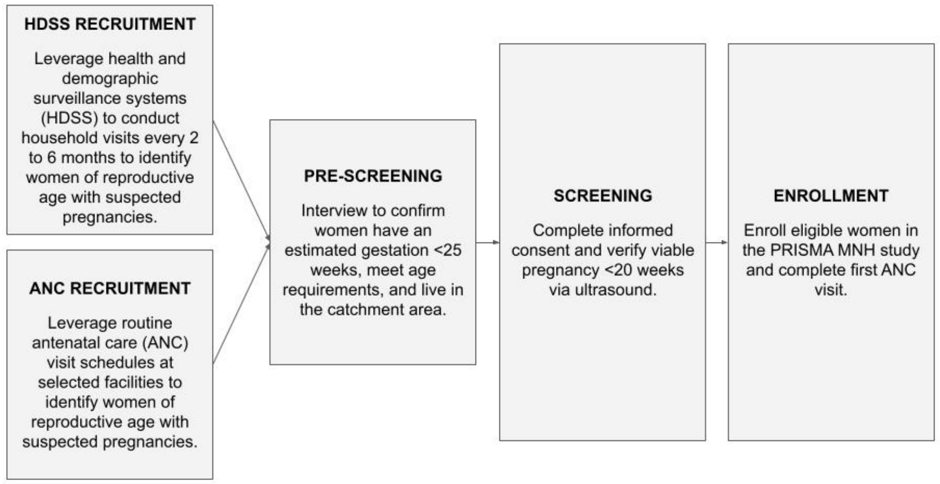

338x190mm (72 x 72 DPI)

SUPPLEMENT 1. OUTCOME DEFINITIONS

Primary Outcomes

1. Maternal mortality: Death from any cause related to or aggravated by pregnancy or its management (excluding accidental or incidental causes) during pregnancy and childbirth or within 42 days of termination of pregnancy, irrespective of the duration and site of the pregnancy.

2. Composite severe maternal outcomes: Composite outcome comprising maternal deaths, late maternal deaths, and maternal near-misses, each defined as the following:

    a. *Maternal death*: Death from any cause related to or aggravated by pregnancy or its management (excluding accidental or incidental causes) during pregnancy and childbirth or within 42 days of termination of pregnancy, irrespective of the duration and site of the pregnancy.

    b. *Late maternal death*: Death from any cause related to or aggravated by pregnancy or its management (excluding accidental or incidental causes), irrespective of the duration and site of the pregnancy, from 42 days postpartum up to one year.

    c. *Maternal near-miss*: Woman who nearly died but survived a life-threatening condition that occurred during pregnancy, childbirth or within 42 days of termination of pregnancy. Consideration as a near-miss case is irrespective of gestational age and includes women who have abortions or stillbirths. Criteria as follows:

        i. Organ dysfunction
        ii. Laparotomy (excluding cesarean section, including hysterectomy)
        iii. Referral to intensive care unit
        iv. Blood transfusion (any units) combined with severe anemia, severe postpartum hemorrhage, antepartum hemorrhage, or placental abruption
        v. Severe postpartum hemorrhage (≥1000 mL estimated blood loss or procedure including hysterectomy and blood transfusion)
        vi. Preeclampsia with severe features/eclampsia
        vii. Uterine rupture
        viii. Sepsis

3. Maternal anemia: Low hemoglobin levels throughout pregnancy and labor and delivery, classified as mild (10-10.9 g/dL), moderate (7-9.9 g/dL), or severe (<7 g/dL). Low hemoglobin levels in the postpartum period, classified as mild (11-11.9 g/dL), moderate (8-10.9 g/dL), or severe (<8 g/dL).

4. Stillbirth: Delivery of a fetus showing no signs of life, as indicated by absence of breathing, heartbeat, pulsation of the umbilical cord, or definite movements of voluntary muscles. Defined as death prior to delivery of a fetus at ≥20 weeks of gestation (or

For peer review only - http://bmjopen.bmj.com/site/about/guidelines.xhtml

>350g weight, if gestational age is unavailable). Additionally, we will analyze time-specific definitions:

a. Early stillbirth: 20-27 weeks,
b. Late stillbirth: 28-36 weeks
c. Term stillbirth: ≥37 weeks
d. WHO-defined stillbirth: ≥28 weeks.

5. Neonatal mortality: Death of a liveborn baby during the first 28 days of life from any cause.

6. Low birthweight: Birth weight <2500 g as assessed at delivery or within 72 hours for home births. Very low birth weight if <1500 g.

7. Preterm birth: Delivery prior to 37 completed weeks of gestation of a liveborn infant. Further classified as: extremely preterm (<28 weeks), very preterm (28-32 weeks), and moderate to late preterm (32-37 weeks). Gestational age at birth will be determined by the best obstetric estimate: last menstrual period, ultrasound, and the American College of Obstetricians and Gynecologists algorithm. Preterm delivery is separately defined as delivery prior to 37 completed weeks of gestation (live or stillbirth).

8. Small for gestational age: Birth weight of an infant of lower than the sex-specific 3rd and 10th percentile for each week of gestational age per INTERGROWTH standards. Combined gestational age information and birthweight will be used to further categorize into preterm-small-for-gestational-age (SGA), preterm-appropriate-for-gestational-age (AGA), term-SGA, and term-AGA.

Secondary Outcomes

9. Late maternal mortality: Death from any cause related to or aggravated by pregnancy or its management (excluding accidental or incidental causes), irrespective of the duration and site of the pregnancy, from 42 days postpartum up to one year.

10. Preeclampsia: Hypertensive disorder of pregnancy defined for those without preexisting hypertension as gestational hypertension AND gestational proteinuria OR clinical diagnosis of preeclampsia OR development of severe features (even in the absence of proteinuria). Defined for those with chronic hypertension as incident gestational proteinuria OR clinical diagnosis of preeclampsia OR development of severe features. Further defined as early-onset preeclampsia if <34 weeks gestation and late-onset if ≥34 weeks. Sub definitions as follows:

    a. *Chronic (preexisting) hypertension*: ≥140/90 mm Hg before pregnancy or <20th week of gestation or use of antihypertensive medication before pregnancy OR history of hypertension OR reported taking antihypertensive medications.

For peer review only

b. *Gestational hypertension*: A systolic blood pressure ≥140 mm Hg on two occasions at least 1 hour apart or diastolic blood pressure ≥90 mm Hg on two occasions at least 1 hour apart during pregnancy.

c. *Gestational proteinuria*: 24-hour urine collection >300 mg protein OR single voided urine protein/creatinine ratio ≥0.3 OR dipstick reading of 1+ or 2+ (use only if other quantitative methods are not available).

d. *Severe features* (indicated by presence of one or more of the following):

i. *Severe gestational hypertension*: during pregnancy defined as systolic blood pressure >160 mmHg or a diastolic blood pressure >110 mmHg on two occasions at least 1 minute apart after 20 weeks gestation; or at the time of delivery defined as one systolic blood pressure >160 mmHg or a diastolic blood pressure >110 mmHg among participants without chronic hypertension >160/110 (even in absence of proteinuria)

ii. *Hepatic abnormality*: impaired liver function not accounted for by another diagnosis and characterized by serum transaminase concentration ≥2 times the upper limit of the normal range

iii. *Severe persistent right upper quadrant or epigastric pain*: unresponsive to medication and not accounted for by an alternative diagnosis

iv. *New onset neurological symptoms*: such as headache (unresponsive to medication and not accounted for by alternative diagnoses) or visual symptoms

v. *Thrombocytopenia*: <100,000 platelets/microL

vi. *Renal insufficiency*: defined as serum creatinine >1.1 mg/dL or a doubling of the serum creatinine concentration in the absence of other renal disease

vii. *Pulmonary edema and/or eclampsia*: clinical report of new-onset tonic-clonic, focal, or multifocal seizures in the absence of other causative conditions such as epilepsy, cerebral arterial ischemia and infarction, intracranial hemorrhage, or drug use

viii. *Evidence of end organ dysfunction*: by laboratory criteria

ix. *Clinical diagnosis of HELLP syndrome*: based upon the presence of ALL of the laboratory abnormalities (hemolysis, elevated liver enzymes, and low platelet count)

1. Hemolysis (established by at least two of the following): peripheral smear with schistocytes and burr cells, serum bilirubin ≥1.2 mg/dL (20.52 umol/L), low serum haptoglobin (≤25 mg/dL) or lactate dehydrogenase (LDH) ≥2 times the upper level of normal (based on

For peer review only

laboratory-specific reference ranges), severe anemia (unrelated to blood loss), and

2. Elevated blood concentrations of liver transaminases (aspartate aminotransferase (AST) or alanine aminotransferase (ALT) to ≥2 times normal concentration (based on laboratory-specific reference ranges)

3. Low platelets or thrombocytopenia <100 x 109/L)

11. Gestational hypertension: A systolic blood pressure ≥140 mm Hg on two occasions at least 1 hour apart or diastolic blood pressure≥90 mm Hg on two occasions at least 1 hour apart or severe gestational hypertension in pregnancy (not at time of delivery) defined as systolic blood pressure > 160 mmHg or a diastolic blood pressure >110 mmHg on two occasions at least 1 minute apart after 20 weeks gestation or severe gestational hypertension at the time of delivery defined as one systolic blood pressure > 160 mmHg or a diastolic blood pressure >110 mmHg among participants without chronic hypertension. Chronic hypertension defined by a clinical diagnosis of chronic hypertension (with or without need for medication) or systolic blood pressure >140 mm Hg on two occasions at least1 hour apart or diastolic blood pressure >90 mm Hg on two occasions at least 1 hour apart before 20 weeks' gestation OR medication used OR prior clinical diagnosis. Medications for hypertension include magnesium sulfate, hydralazine, methyldopa (aldomet), atenolol, nifedipine, betamethasone, labetalol, and dexamethasone.

12. Postpartum hypertension: Systolic blood pressure ≥140 mm Hg or diastolic blood pressure ≥90 mm Hg on two occasions at least 1 hour apart in the postpartum period.

13. Preterm birth indication: Delivery prior to 37 completed weeks of gestation of a birth (live or stillbirth), classified into spontaneous preterm or provider-initiated preterm. Spontaneous preterm is defined as delivery <37 weeks that occurs either secondary to preterm labor or preterm premature rupture of membranes. Provider-initiated preterm defined as a medical or obstetric complication or other reason that the health care provider initiates delivery at <37 completed weeks gestation.

14. Preterm premature rupture of membranes: Rupture of membranes before the onset of labor occurring before <37 weeks of gestation or clinical diagnosis of premature rupture of membranes.

15. Gestational diabetes: Diabetes first diagnosed during pregnancy and characterized by insulin resistance and high blood sugar. Clinical diagnosis or diagnosis by 75g oral glucose tolerance test (OGTT) around 28 weeks based on the International Association of the Diabetes and Pregnancy Study Groups thresholds: fasting > 5.1 mmol/L or 1-hr OGTT >10.0 mmol/L or 2-hr OGTT >8.5 mmol/L.

16. Maternal infection and sepsis: Defined as organ dysfunction (per WHO near-miss definition) resulting from infection during pregnancy, delivery, and over 42 days

For peer review only

postpartum. Categories of infection are defined based on severity, as defined by the Global Maternal Sepsis Study:

a. *Infection-related severe maternal outcomes*: women presenting with WHO near-miss criteria to define organ system dysfunction and maternal death

b. *Infections with complications*: women with an invasive procedure to treat the source of infection (vacuum aspiration, dilatation and curettage, wound debridement, drainage, laparotomy and lavage, other surgery), or admission to ICU, or transfer to another facility

c. *Less severe infections*: all other infections.

17. Perinatal depression: Defined as a clinical mood disorder during pregnancy and up to 42 days postpartum. Symptoms screened using the Edinburgh Postnatal Depression Scale. Threshold for defining depression will be country specific based on local validation studies: Kenya ≥13; Ghana: ≥11; Pakistan ≥14; India (Vellore): ≥8; India (Hodal) ≥10; Zambia ≥10.

18. Fetal death: A product of human conception, irrespective of the duration of the pregnancy, which, after expulsion or extraction, does not breath or show any other evidence of life such as beating of the heart, pulsation of the umbilical cord, or definite movement of voluntary muscles, whether or not the umbilical cord has been cut or the placenta is attached.

19. Infant mortality: Death of a neonate or an infant from delivery up to one year of life.

20. Cause of neonatal mortality: Causes of neonatal death based on verbal autopsy.

21. Timing of neonatal mortality: Death of a liveborn during the first 28 days of life. Neonatal deaths are classified by timing: <24 hours (i.e. first 24 hours of life), early neonatal mortality (i.e. first 7 days), or late neonatal mortality (i.e. between 7 and 28 days).

22. Cause of stillbirth: Causes of stillbirth based on verbal autopsy.

23. Timing of stillbirth: Delivery of a fetus showing no signs of life, as indicated by absence of breathing, heartbeat, pulsation of the umbilical cord, or definite movements of voluntary muscles. Death occurring prior to delivery of a fetus at ≥20 weeks of gestation (or >350 g weight, if gestational age is unavailable). . Antepartum stillbirth, defined as heart rate not detected ≥12 hrs prior to delivery and/or signs of maceration. Intrapartum stillbirth, defined as heart rate detected <12 hrs prior to delivery and/or no signs of maceration.

24. Hyperbilirubinemia: Defined as the presence of excess bilirubin during the first week of life (assessed at birth, 3 days, and 7 days of life). Outcome based on: continuous bilirubin by age (hours), proportion >75th percentile, and proportion >95th percentile.

25. Neonatal sepsis: Defined as inflammatory response and organ dysfunction following presence of a severe infection from delivery to 28 days as suspected (by a clinician) or

For peer review only

proven (with culture). Further classified as early onset (i.e. delivery to 72 hours) or late onset (72 hours to 28 days).

26. Possible severe bacterial infection: Presence of any of the following clinical signs or symptoms as defined by the WHO IMCI criteria, which are consistent with possible severe bacterial infection, at the time of delivery to 59 days: not able to feed at all or not feeding well, convulsions, severe chest indrawing, high body temperature (≥38°C), low body temperature (less than 35.5°C), no movement/only when stimulated, and/or fast breathing (≥60 BPM) in infants less than 7 days old.

27. Postnatal weight trajectory: Continuous weight change from birth through one month of age. Trajectory is determined by percent body weight loss by 72 hours, time to regain birth weight, and continuous body weight.

28. Infant growth: Proportion of infants more than 2 standard deviations below the WHO Children Health Reference Growth Standard for: weight for age (underweight), length for age (stunting), length for weight (wasting), and/or head circumference for age (microcephaly).

Tertiary Outcomes

29. Placental disorders: Placental abruption is defined as the complete or partial separation of the placenta from the inner wall of the uterus before delivery. Signs include vaginal bleeding (painful or painless), pain during abdominal palpitations, and/or fetal distress. Diagnosis made via ultrasound. Placenta previa is defined at the partial or complete covering of the cervix by the placenta. Signs include vaginal bleeding without pain in the second or third trimester. Diagnosis made via ultrasound.

30. Uterine rupture: Uterine rupture is defined as the spontaneous tearing of the uterus prior to delivery that may result in the fetus being expelled into the peritoneal cavity. Symptoms and signs of uterine rupture include fetal bradycardia, variable decelerations, evidence of hypovolemia, loss of fetal station (detected during cervical examination), severe or constant abdominal pain, and cessation of uterine contractions, and/or recession of the presenting part.

31. Prolonged labor: Defined as labor lasting ≥24 hours.

32. Unplanned surgery: The occurrence of medically indicated or emergent surgical procedure during labor and delivery. Hysterectomy is defined as the removal of the uterus (i.e. partial) or of the uterus and the cervix (i.e. total hysterectomy). Cesarean section is defined as a surgical procedure used to deliver a baby through incisions in the abdomen and uterus.

33. Hemorrhage:

    a. *Antepartum hemorrhage*: Bleeding from or into the genital tract, occurring from ≥24 weeks gestation and prior to delivery.

For peer review only

b. *Postpartum hemorrhage*: Defined as estimated blood loss of 500 mL or more within 24 hours after birth or clinician diagnosis.

c. *Severe postpartum hemorrhage*: perceived abnormal bleeding (1000 ml or more) or any bleeding with hypotension or blood transfusion.

34. Perinatal birth asphyxia: Clinician-reported failure to breathe spontaneously in the first minute after delivery.

For peer review only
